# Exosomal miR-146a-5p and miR-155-5p promote CXCL12/CXCR7-induced metastasis of colorectal cancer by crosstalk with cancer-associated fibroblasts

**DOI:** 10.1038/s41419-022-04825-6

**Published:** 2022-04-20

**Authors:** Dong Wang, Xiaohui Wang, Yujia Song, Mahan Si, Yuqi Sun, Xiaohui Liu, Shuxiang Cui, Xianjun Qu, Xinfeng Yu

**Affiliations:** 1 Department of Pharmacology, School of Basic Medical Sciences, Capital Medical University, Beijing, China; 2 Department of General Surgery, Xuanwu Hospital, Capital Medical University, Beijing, China; 3 Department of Toxicology and Sanitary Chemistry, School of Public Health, Capital Medical University, Beijing, China

**Keywords:** Cancer microenvironment, Colon cancer

## Abstract

C-X-C motif chemokine receptor 7 (CXCR7) is a newly discovered atypical chemokine receptor that binds to C-X-C motif chemokine ligand 12 (CXCL12) with higher affinity than CXCR4 and is associated with the metastasis of colorectal cancer (CRC). Cancer-associated fibroblasts (CAFs) have been known to promote tumor progression. However, whether CAFs are involved in CXCR7-mediated metastasis of CRC remains elusive. We found a significant positive correlation between CXCR7 expression and CAF activation markers in colonic tissues from clinical specimens and in *villin-CXCR7* transgenic mice. RNA sequencing revealed a coordinated increase in the levels of miR-146a-5p and miR-155-5p in CXCR7-overexpressing CRC cells and their exosomes. Importantly, these CRC cell-derived miR-146a-5p and miR-155-5p could be uptaken by CAFs via exosomes and promote the activation of CAFs through JAK2–STAT3/NF-κB signaling by targeting suppressor of cytokine signaling 1 (SOCS1) and zinc finger and BTB domain containing 2 (ZBTB2). Reciprocally, activated CAFs further potently enhanced the invasive capacity of CRC cells. Mechanistically, CAFs transfected with miR-146a-5p and miR-155-5p exhibited a robust increase in the levels of inflammatory cytokines interleukin-6, tumor necrosis factor-α, transforming growth factor-β, and CXCL12, which trigger the epithelial–mesenchymal transition and pro-metastatic switch of CRC cells. More importantly, the activation of CAFs by miR-146a-5p and miR-155-5p facilitated tumor formation and lung metastasis of CRC in vivo using tumor xenograft models. Our work provides novel insights into CXCR7-mediated CRC metastasis from tumor–stroma interaction and serum exosomal miR-146a-5p and miR-155-5p could serve as potential biomarkers and therapeutic targets for inhibiting CRC metastasis.

## Introduction

C-X-C motif chemokine receptor 7 (CXCR7), also known as atypical chemokine receptor 3 (ACKR3), is a seven-transmembrane G-protein coupled receptor (GPCR). It is a newly discovered chemokine receptor for C-X-C motif chemokine ligand 12 (CXCL12) and is highly expressed in multiple cancers and plays a crucial role in tumor growth and metastasis [1]. It has been reported that CXCL12/CXCR7 promotes lung metastasis of colorectal cancer (CRC) [2, 3]; however, the underlying mechanism remains poorly understood.

Cancer-associated fibroblasts (CAFs) are the main stromal cells in the tumor microenvironment (TME) and play crucial roles in tumor development and metastasis. CAFs are abundant fibroblasts that acquire α-smooth muscle actin (α-SMA)-positive, activated myofibroblast phenotype [4]. They originate predominantly from tissue-resident fibroblasts that are activated in response to signals from cancer cells and the TME. Although there is significant phenotypic heterogeneity within the CAF population, CAFs promote malignant cell growth and metastasis through the remodeling of extracellular matrix (ECM) and secretion of angiogenic factors [5]. CAFs have been proposed to promote the expression of the inflammatory cytokines and downstream activation of nuclear factor-κB (NF-κB), SMADs, and JAK–STAT3 signaling pathways [6, 7].

Exosomes, a class of extracellular vesicles—50–150 nm in diameter, mediate the bidirectional communication between tumors and CAFs by transporting cargo, including proteins, lipids, and nucleic acids [8]. Cancer cell-derived exosomal miRNAs have been shown to play a dominant role in educating the stromal cells, particularly converting fibroblasts into CAFs, which in turn contributes to metastasis in many tumors [9, 10]. Therefore, CAFs have been used as therapeutic targets to inhibit metastasis. However, it remains as a big challenge to elucidate the signaling molecules mediating intercellular communication between CAFs and tumor cells that are critical for cancer metastasis.

In the present study, we hypothesized that activation of CXCL12/CXCR7 could promote the release of exosomal miRNAs in CRC cells, leading to the activation of CAFs by regulating the target genes. In turn, activated CAFs further facilitate distant metastasis by secreting cytokines. Our results revealed the potential clinical utility of exosomes as novel carriers of miRNAs for inhibiting metastasis of CRC upon activation of the CXCL12/CXCR7 axis by exerting effects on CAFs, providing potential therapeutic strategies and drug targets.

## Materials and methods

### Cell culture and cell transfection

Human CRC cell lines HCT116 and SW620 were purchased from the American Type Culture Collection (ATCC) and were cultured in RPMI 1640 medium supplemented with 10% fetal bovine serum (FBS). Human embryonic lung cell MRC-5 was purchased from KeyGen Biotech. Company (Nanjing, China) and cultured in Dulbecco’s modified Eagle’s medium (DMEM) supplemented with 10% FBS (Gibco) and non-essential amino acids (KGY0091, KeyGen). The cells were incubated at 37 °C in a humid atmosphere (5% CO_2_) and were authenticated by short tandem repeats (STR) profiling.

The CRC cells were infected with lentivirus expressing CXCR7 and luciferase (LV-CXCR7-Luc) and vector control HBLV-CMV-MCS-3flag-EF1-LUC-T2A-PURO (Hanbio Biotechnology, Shanghai, China). Silencing of CXCR7 was performed by using CXCR7 siRNAs (GenePharma, China). The CXCR7 siRNA sequence-1 and -2 were 5′-CGCUCUCCUUCAUUUACAUUU-3′ and 5′-GCCGUUCCCUUCUCCAUUAUC-3′, respectively. To determine the roles of miR-146a-5p and miR-155-5p, MRC-5 cells were transfected with 100 nM of these miRNAs mimics or inhibitors (GenePharma, China) using lipofectamine 2000 transfection reagents (Invitrogen, USA) according to the manufacturer’s instructions.

### Isolation of CAFs from CRC tisssues

Mouse CAFs were isolated from colonic tumors of C57BL/6J mice according to the methods described previously [11]. Briefly, colonic cancer tissues were minced and dissociated in DMEM containing 0.1 mg/ml collagenase II (Worthington Biochem) for 2 h at 37 °C in a thermomixer. The homogenate passed through 100 µm cell strainers to remove the undigested tissue, the filtered solution was centrifuged and the pellet was resuspended in serum-free DMEM followed by purification using differential time adherent method. The CAFs were cultured in DMEM containing 10% FBS for further study.

### Clinical specimens

Human CRC tissue specimens and adjacent normal mucosa were obtained from CRC patients who underwent surgery in Xuanwu Hospital of Capital Medical University (Beijing, China) and were confirmed by pathological analysis (*n* = 24). The clinicopathological features are shown in Table S1. The tissues were stored in RNA*later* solution (Qiagen, USA) at −80 °C. Human serum samples were collected from CRC patients (*n* = 17) including metastatic (*n* = 7) and non-metastatic (*n* = 10) CRC patients, as well as healthy donors (*n* = 13). The blood samples were centrifuged at 3000*g* for 10 min to extract the serum. All procedures were conducted with the approval of the Institutional Review Board of Xuanwu Hospital of Capital Medical University (2020SY007). The informed consents were obtained from all patients. The study methodologies conformed to the standards set by the Declaration of Helsinki.

### Isolation and identification of exosomes

For exosomes isolation, the HCT116 cells infected with vector control and LV-CXCR7-Luc were washed twice with phosphate-buffered saline (PBS) and replenished with fresh FBS-free RPMI 1640 medium. Culture media were collected after 24 h for exosomes isolation by ultracentrifugation at 100,000*g* for 70 min (Beckman Coulter, USA) according to the methods described previously [12]. The serum exosomes were isolated by a precipitation method using Exoquick exosome precipitation solution (System Biosciences, USA) according to the manufacturer’s instructions as described previously [13].

The morphology and size of exosomes were observed using transmission electron microscopy (JEM-2100 JEOL, Tokyo, Japan) as described previously [13]. The exosomes were identified by determining the expression of specific markers CD9, CD81, and CD63 for exosomes in contrast with the endoplasmic reticulum (ER) marker Calnexin in cell lysates.

### Exosomes labeling and tracing

Exosomes were fluorescently labeled using PKH26 membrane dye (Sigma-Aldrich, USA) according to the manufacturer’s instructions. Then the labeled exosomes were washed with PBS and then incubated with the recipient cells MRC-5 for 48 h to determine the uptake of exosomes.

For the tracing of exosomal miRNAs, the HCT116 cells were transfected with FAM-labeled miR-146a-5p or miR-155-5p and co-cultured with MRC-5 cells for 48 h. The transfer of the FAM-labeled miRNAs was observed by confocal microscopy TCS SP5 (Leica, Germany). The nucleus was stained with DAPI.

### miRNA sequencing

Total RNA and exosomal RNA were extracted from HCT116 cells infected with vector control and lenti-CXCR7-Luc using a mirVana miRNA Isolation Kit (Invitrogen, USA). Cellular miRNA and exosomal miRNA sequencing were performed on a BGISEQ-500 platform by Beijing Genomics Institute (BGI, Wuhan, China). Raw reads were filtered using SOAPnuke software and mapped to Genome Reference Consortium human Build 38 (GRCh38.p12) using HISAT software. Differentially expressed genes (fold change ≥2 with *p* < 0.05) were analyzed using DESeq2. The raw data of miRNA sequencing have been submitted to Sequence Read Archive (SRA) data with BioProject accession number PRJNA784641.

### Immunohistochemistry (IHC)

IHC staining of colorectal tissues was performed on 5-µm-thick formalin-fixed and paraffin-embedded (FFPE) tissue sections according to the standard procedures as described previously [14]. The sections were stained using antibodies against CXCR7 (ab72100) (Abcam, USA), α-SMA (55135-1-AP), Vimentin (10366-1-AP), and Ki67 (27309-1-AP) (Proteintech, USA) according to the manufacturers’ instructions. The IHC results were taken with a digital slide scanning system (Pannoramic Scan, 3DHISTECH Ltd) and semiquantified of mean density (IOD/area) by image-pro plus 6 software (IPP, USA).

### Reverse transcription PCR (RT-PCR) analysis

Reverse transcription of miRNA was performed by a miScript reverse transcription kit (Qiagen, USA) according to the manufacturer’s protocol. miScript SYBR Green PCR kit (Qiagen, USA) and miR-146a-5p and miR-155-5p specific primers were used to determine the expression of mature miRNAs. RNU6B was used as an internal control. Cel-miR-39 was used to normalize for technical variation between exosomes samples as described previously [15].

To determine mRNA expression, we performed reverse transcription using ReverTra Ace qPCR RT kit and SYBR Green Realtime PCR kit (TOYOBO, Japan) according to the manufacturer’s instructions on 7500 Fast Real-Time PCR System (Applied Biosystems, USA) as described previously [14]. The sequences of all indicated primers are listed in Table S2.

### Western blotting

Cells or exosomes were lysed in RIPA buffer (Beyotime, China) supplemented with 1 mM PMSF and complete protease inhibitor mixture (Roche Pharmaceuticals, Switzerland) on ice. The total protein was quantified using a Pierce BCA protein assay Kit (Thermo Scientific, USA). An equal amount of protein lysate was loaded onto SDS-PAGE gels and transferred to PVDF membranes (Millipore, USA). The membranes were blocked and incubated overnight at 4 °C with primary antibodies against CD9 (ab92726), CD81 (sc-166029), CD63 (sc-5275), Calnexin (sc-80645) (Santa Cruz Biotechnology, USA), CXCR7 (ab72100), Fibroblast activation protein (FAP) (ab53066) (Abcam, USA), E-cadherin (3195), N-cadherin (13116), Vimentin (5741), Snail (3879), p-STAT3 (9145), STAT3 (12640), p-JAK2(3771), JAK2 (3230), p-NF-κB (3033), NF-κB (8242) (Cell Signaling Technology, USA), α-SMA (55135-1-AP), β-actin (66009-1-Ig) and GAPDH (60004-1-Ig) (Proteintech, USA) followed by HRP-conjugated secondary antibodies. Immunoreactive products were visualized using Fluorchem FC3 system (ProteinSimple, USA) by chemiluminescence (Millipore, USA) and quantified by densitometry using AlphaView software. Densitometric analyses of the bands were normalized with β-actin or GAPDH that function as loading controls.

### Luciferase reporter assay

The 3′UTR of *ZBTB2* gene containing miR-146a-5p-binding site was synthesized and cloned into GV272 control vectors (GeneChem, China) to make the luciferase constructs. The luciferase activity was determined by a dual-luciferase reporter assay system (Promega, USA). Briefly, HCT116 cells were co-transfected with wild type or mutant ZBTB2-3′UTR-Luc firefly luciferase constructs and miR-146a-5p mimics using lipofectamine 2000 reagent. The pRL-SV40 plasmid was co-transfected and the renilla luciferase activity was used for normalization. All assays were performed in triplicate and each experiment was repeated three times.

### Transwell assay

The CRC cells (HCT116 and SW620) were suspended in serum-free medium and seeded into the transwell top chambers with inserts of 8 μm pore size (Corning, 3422). Conditioned medium (CM) derived from MRC-5 cells transfected with 100 nM miR-146a-5p and miR-155-5p mimics were added to the bottom chamber. After 24 h, the cells that had migrated through the membrane and adhered to the lower surface of the membrane were stained with 0.2% crystal violet, and the percentage of migrated cells normalized to the total cells were observed and analyzed under a light microscope.

### Animal models

*Villin-CXCR7* transgenic mice (*CXCR7*^*+/−*^) overexpressing CXCR7 in intestinal epithelial cells were generated by Cyagen Biosciences Inc. (Guangzhou, China) and genotyping was performed as described previously [16]. The establishment of mouse models of colitis-associated CRC by treatment of azoxymethane (AOM)/dextran sodium sulfate (DSS) was conducted as previously described [12]. In brief, 8 weeks old C57BL/6 mice (*n* = 5 per group) were treated by a single intraperitoneal injection of AOM (10 mg/kg, Sigma-Aldrich) and subsequent oral administration of 1% DSS (MP Biomedicals) in drinking water ad libitum for seven consecutive days and then returned to normal drinking for 14 days intervals. Three cycles of DSS are performed to establish colitis-associated cancer.

Six-week-old female athymic *Balb/c-nu/nu* mice were purchased from Charles River Laboratories (Beijing, China) and maintained in a specific pathogen-free environment. The nude mice were grouped by randomization (*n* = 3 for each group). To examine the tumor-promoting effects of MRC-5 cells overexpressing miR-146a-5p and miR-155-5p on CRC cells, HCT116 cells mixed with MRC-5 cells transfected with miR-146a-5p and miR-155-5p (3:1) were injected subcutaneously into the armpits of nude mice. The tumor growth was measured by calipers every 3 days and tumor volumes were calculated using the formula volume (mm^3^) = *L* × *W*^2^/2 (length *L*, mm; width *W*, mm).

For the lung metastasis assay, 1 × 10^6^ HCT116 cells treated for 48 h with the CM from MRC-5 cells overexpressing miR-146a-5p and miR-155-5p were injected into the tail veins of nude mice. After 4 weeks, the nude mice were sacrificed and the lungs were removed for HE staining. All animal experiments were approved by the Institutional Animal Care and Use Committee of Capital Medical University. The ethics number was AEEI-2021-194.

### Bioinformatics

Gene Expression Profiling Interactive Analysis (GEPIA) (http://GEPIA.cancer-pku.cn/index.html) was used to analyze the correlation of the expression of CXCR7 with α-SMA or FAP as well as the expression of CXCR7 at different pathological stages in CRC patients. The association of the expression of CXCR7 with overall survival was analyzed by GEPIA using TCGA datasets in CRC and gastrointestinal cancer. The patients were divided with high and low gene expression levels using the median cutoff and log-rank *p* value was shown.

### Statistical analysis

All values are reported as the mean ± SD from three independent experiments. Differences between treated and control groups were analyzed using the *t*-test or one-way analysis of variance. A two-tailed value of *p* < 0.05 was considered statistically significant. All statistical analyses were performed with the SPSS 20.0 statistical software.

## Results

### High expression of CXCR7 is associated with CAF activation in metastatic CRC

**Fig. 1:**
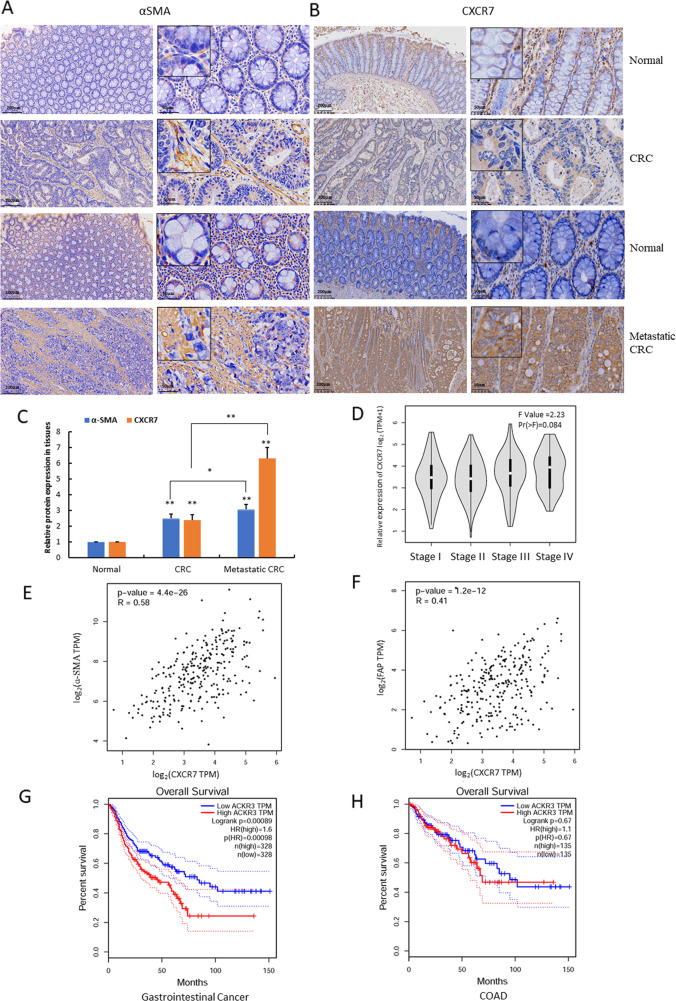
High expression of CXCR7 promotes CAF activation in metastatic CRC. **A**, **B** Expression of α-SMA and CXCR7 was analyzed by IHC in paired human CRC tissues (metastatic and non-metastatic CRC) and the corresponding adjacent normal colonic tissues (*n* = 24, scale bar = 200, 50 μm). **C** Relative expression of CXCR7 and α-SMA in different groups was semi-quantified of mean density (IOD/area) using image-pro plus 6 software. *p < 0.05, **p < 0.01. **D** Box plots showed the relative expression of CXCR7 at different pathological stages and were performed by Gene Expression Profiling Interactive Analysis (GEPIA). Relative expression of CXCR7 was shown as log_2_ (TPM + 1), TPM represents transcripts per million. **E**, **F** The correlation of expression of CXCR7 (log_2_ CXCR7 TPM) with expression of α-SMA (log_2_ ɑ-SMA TPM) and FAP (log_2_ FAP TPM) was determined in CRC tissues by GEPIA. TPM represents transcripts per million. **G**, **H** The association of the expression of CXCR7 (ACKR3) with overall survival was analyzed by GEPIA using TCGA datasets in gastrointestinal cancer patients (COAD plus STAD) and CRC patients (COAD). The patients were divided with high and low gene expression levels using the median cutoff and log-rank *p* value was shown. COAD colonic adenocarcinoma; STAD stomach adenocarcinoma.

To evaluate the pathological significance and clinical relevance of CXCR7 expression with CAF activation in CRC tissues, we determined the expression of CXCR7 and the representative CAF marker α-SMA by IHC staining. The results indicated that CXCR7 and α-SMA were overexpressed in CRC tissues compared to normal colonic tissues (Fig. 1A, B). More importantly, the expression levels of CXCR7 and α-SMA were significantly higher in metastatic CRC than in non-metastatic CRC, suggesting that activation of CAFs was possibly associated with the metastasis of CRC (Fig. 1C). Further gene expression profiling interactive analysis (GEPIA) indicated that the expression of CXCR7 is possibly associated with tumor stages although the difference was not statistically significant (*P* = 0.084) (Fig. 1D). Notably, the expression of CXCR7 was highly correlated with the expression of α-SMA and FAP (*R* = 0.58 and 0.41, respectively) (Fig. 1E, F). The results suggest that high expression of CXCR7 may be associated with activation of CAFs, which contributes to the metastasis of CRC.

Aberrant CXCR7 expression has been linked to metastasis and poor clinical outcomes [17, 18]. To explore the potential link between CXCR7 expression and patient prognosis, we used GEPIA and found that high expression of CXCR7 was significantly associated with poor overall survival of gastrointestinal cancer patients (Logrank *p* < 0.05) but not associated with overall survival in CRC patients alone (Fig. 1G, H), probably due to different sample size or the variation of CXCR7 expression between transcriptional and translational levels.

**Fig. 2:**
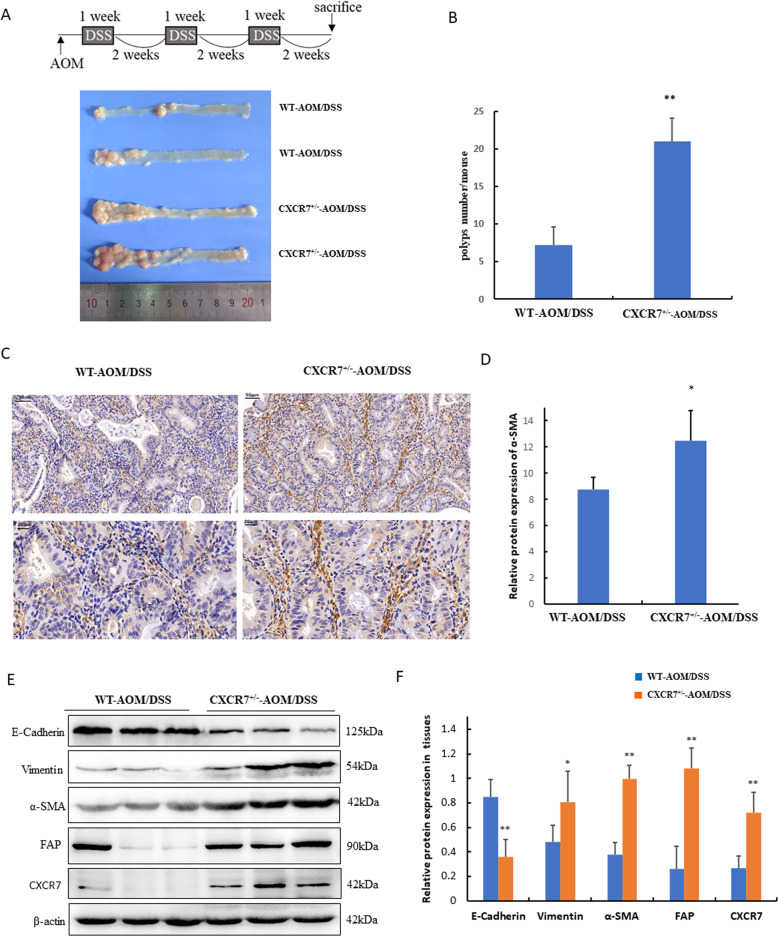
CXCR7 promotes CAF activation in transgenic mice. **A** The schematic regimen of colitis-associated cancer model by treatment with three cycles of AOM/DSS. Representative images of colons from AOM/DSS-treated WT (*n* = 8) and *Villin-CXCR7* (*CXCR7*^+/−^) transgenic mice (*n* = 8). **B** Total polyp counts per mouse were examined in different groups (*n* = 8). **p < 0.01 vs. WT mice. **C**, **D** IHC analysis of the expression of α-SMA in colonic tissues of AOM/DSS-induced WT and *Villin-CXCR7* (*CXCR7*^+/−^) transgenic mice and statistical analysis was performed. *p < 0.05 vs. WT mice (*n* = 6), scale bar = 50, 20 μm. **E**, **F** Western blot analysis of the expression of E-cadherin, Vimentin, α-SMA, FAP, and CXCR7 in colonic tissues of AOM/DSS-induced WT and *Villin-CXCR7* (*CXCR7*^+/−^) transgenic mice normalized to β-actin and statistical analyses were performed. *p < 0.05, **p < 0.01 vs. WT mice.

To further confirm the potential association between CXCR7 expression and activation of CAFs in vivo, we treated *villin-CXCR7* transgenic mice (*CXCR7*^*+/−*^) with AOM/DSS to establish the colitis-associated cancer model. PCR results showed the genotyping of the *villin-CXCR7* transgenic mice (Fig. S1). The results revealed that the number of colonic adenomas in *CXCR7*^+/−^ mice was significantly higher than that in WT mice (20.60 ± 3.61 vs. 10.20 ± 4.21, *n* = 5, *p* < 0.05) (Fig. 2A, B). Notably, IHC staining of CAF markers in colonic tissues indicated that α-SMA was predominantly located in fibroblasts of interstitial space and showed more intense staining in colonic tissues of *CXCR7*^+/-^ mice than in WT mice (Fig. 2C, D).

Epithelial–mesenchymal transition (EMT) plays a critical role in triggering cancer metastasis. To prove the role of CXCR7 in EMT of CRC and its potential link with activation of CAFs, we performed western blotting and found that high expression of CXCR7 remarkably promoted EMT as indicated by the significant decrease in E-cadherin expression and marked increase in Vimentin expression in colonic tissues of *CXCR7*^+/−^ mice. Concurrently, the expression of CAF activation markers α-SMA and FAP was also enhanced in these mice (Fig. 2E, F). These results implied that high expression of CXCR7 in CRC cells possibly promotes the activation of CAFs, which in turn contributes to EMT and metastasis of CRC.

### Activation of the CXCL12/CXCR7 axis upregulates miR-146a-5p and miR-155-5p in both the CRC cells and their exosomes

**Fig. 3:**
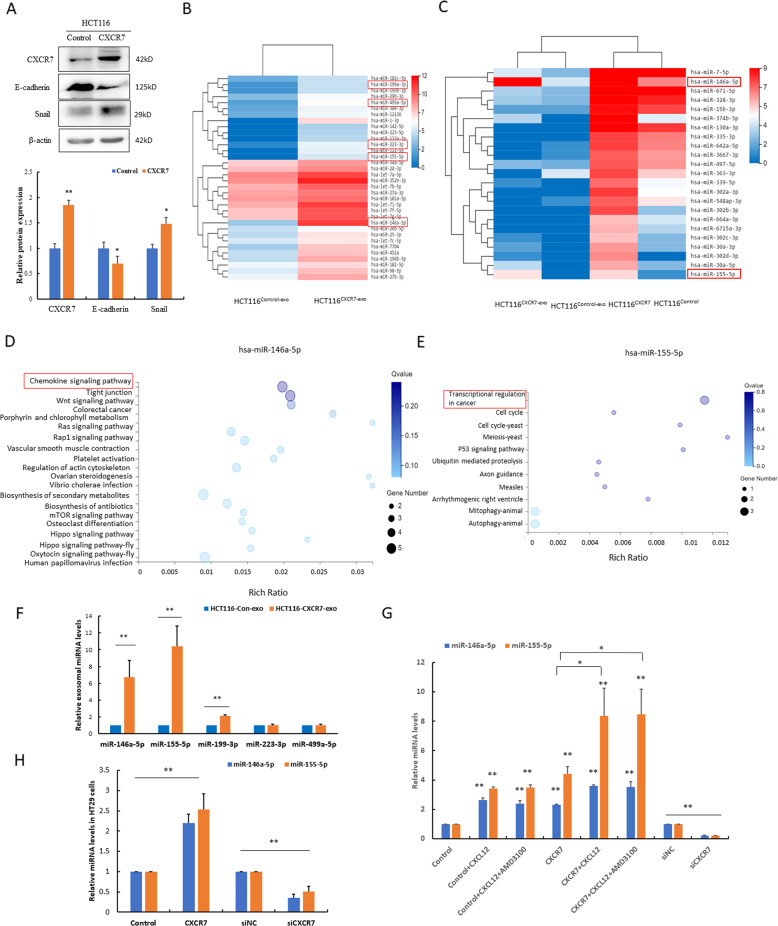
Activation of the CXCL12/CXCR7 axis upregulated miR-146a-5p and miR-155-5p in both the CRC cells and their exosomes. **A** Western blot analysis of the expression of CXCR7, E-cadherin, Snail in HCT116 cells infected with lenti-CXCR7 and control and statistical analysis was performed. *p < 0.05 vs. control, **p < 0.01 vs. control. **B**, **C** RNA sequencing was performed in both the exosomes and HCT116 cells infected with lenti-CXCR7 and control. Hierarchical clustering analysis of differentially expressing miRNAs was shown. The red box indicates the most upregulated miRNAs. **D**, **E** Enrichment bubble pattern shows the enrichment of predicted target genes of miR-146a-5p and miR-155-5p. *X*-axis represents rich ratio and *Y*-axis represents GO term. The size of bubble represents the gene number. *Q* value signifies statistical significance. **F** RT-qPCR analysis of the levels of miR-146a-5p, miR-155-5p, miR-199-3p, miR-223-3p, and miR-499a-5p in exosomes derived from HCT116^Control^ and HCT116^CXCR7^ cells. **p < 0.01 vs. control. **G**, **H** RT-qPCR analysis of the levels of miR-146a-5p and miR-155-5p in HCT116 and HT29 cells infected with siRNA or lenti-CXCR7 stimulated by CXCL12 (100 ng/ml) with or without AMD3100 as indicated. *p < 0.05, **p < 0.01.

Since CXCR7 promoted EMT in AOM/DSS-induced colitis-associated cancer in vivo, we next infected HCT116 cells with vector control and CXCR7 overexpression lentivirus to establish the stable cell line (named HCT116^Control^ and HCT116^CXCR7^, respectively). As expected, high expression of CXCR7 remarkably promoted EMT by downregulation of E-cadherin and upregulation of Snail (Fig. 3A). To corroborate that CXCR7-overexpressing CRC cells contribute to distant metastasis by promoting activation of CAFs in vivo, we injected luciferase-labeled HCT116^Control^ and HCT116^CXCR7^ cells into nude mice via the tail vein. The results indicated that high CXCR7 expression significantly enhanced metastasis by in vivo bioluminescence imaging (Fig. S2A, B). IHC analysis further confirmed the remarkable expression of α-SMA in metastatic carcinoma (Fig. S2C). To explore the mechanism underlying the high expression of CXCR7 in the activation of CAFs, we further analyzed the miRNA expression profiles in the exosomes derived from these cells (named HCT116^Control-exo^ and HCT116^CXCR7-exo^, respectively). RNA sequencing results indicated that miR-155-5p, miR-146a-5p, miR-199-3p, miR-499a-5p, and miR-223-3p were among the most upregulated miRNAs in the HCT116^CXCR7-exo^ group compared to the HCT116^Control-exo^ group (Table S3 and Fig. 3B). Notably, when we analyzed the expression profiles of both cellular and exosomal miRNAs, the levels of miR-146a-5p and miR-155-5p rank the top highest in HCT116^CXCR7^ cells and their exosomes compared with those of the controls (Fig. 3C and Table S4). Further GO functional analysis indicated that the enrichment of predicted target genes of miR-146a-5p and miR-155-5p was closely associated with the chemokine signaling pathway and transcriptional regulation in cancer (Fig. 3D, E).

To confirm the sequencing results, we performed RT-qPCR analysis and found that miR-155-5p, miR-146a-5p, and miR-199-3p were robustly increased in the HCT116^CXCR7-exo^ group compared to the HCT116^Control-exo^ group (Fig. 3F). Furthermore, the levels of miR-146a-5p and miR-155-5p were substantially upregulated in HCT116^CXCR7^ or stimulated by CXCL12. Since CXCL12 is a common ligand of CXCR4 and CXCR7, to exclude the effects of CXCR4 on the regulation of these miRNAs, we added the CXCR4 specific inhibitor AMD3100. Importantly, pretreatment with AMD3100 did not significantly reduce the levels of miR-146a-5p and miR-155-5p, suggesting that CXCL12 stimulation induces the expression of these miRNAs mainly through the activation of the CXCL12/CXCR7 axis. In contrast, silencing of CXCR7 by siRNAs potently attenuated the expression levels of miR-146a-5p and miR-155-5p in HCT116 cells (Fig. 3G). As expected, similar results were observed in HT29 cells (Fig. 3H).

### Tumor cell-derived miR-146a-5p and miR-155-5p are uptaken by CAFs via exosomes and promote activation of fibroblasts

**Fig. 4:**
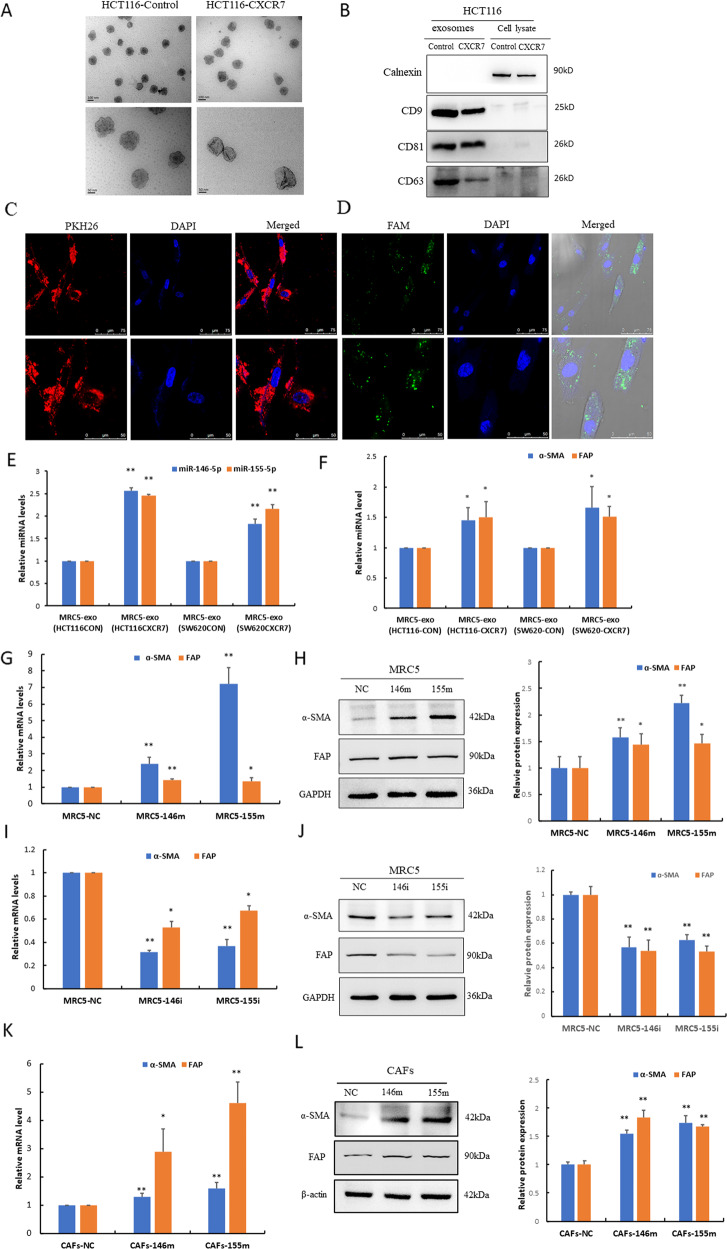
Tumor cell-derived miR-146a-5p and miR-155-5p were uptaken by CAFs via exosomes and activated fibroblasts. **A** Transmission electron micrograph of exosomes derived from HCT116^CXCR7^ and HCT116^Control^ cells (scale bar = 100, 50 nm). **B** Western blot analysis of exosomal markers CD9, CD63, CD81 in isolated exosomes, and calnexin expression in cell lysate as the control. **C** Internalization of PKH26-labeled exosomes by MRC-5 cells was examined by confocal microscopy after 48 h of co-culture (scale bar = 75 μm for top panel, scale bar = 50 μm for bottom panel). **D** Transfer of FAM-labeled miRNAs from HCT116 cells to MRC-5 cells was observed by confocal microscopy after 48 h of co-culture (scale bar = 75 μm for top panel, scale bar = 50 μm for bottom panel). **E**, **F** RT-qPCR analysis of the expression levels of miR-146a-5p and miR-155-5p, as well as the mRNA levels of α-SMA and FAP in MRC-5 cells treated with exosomes derived from HCT116^CXCR7^ and SW620^CXCR7^ cells and their corresponding controls. **G**–**J** RT-qPCR and western blot assays were performed to determine the levels of α-SMA and FAP mRNAs and proteins in MRC-5 cells transfected with miR-146a-5p and miR-155-5p mimics (146m, 155m) and inhibitors (146i, 155i). **K**, **L** RT-qPCR and western blot assays were performed to determine the levels of α-SMA and FAP expression in primary cultures of CAFs transfected with miR-146a-5p and miR-155-5p mimics. β-actin functions as a loading control. *p < 0.05 vs. control **p < 0.01 vs. control.

We hypothesized that tumor-derived exosomal miRNAs enhance CRC metastasis by driving EMT in HCT116^CXCR7^ cells through activation of the fibroblasts. To prove this, we first isolated exosomes from HCT116^Control^ and HCT116^CXCR7^ cells. These exosomes exhibited a bilayer cup-shaped morphology with 100–150 nm in diameter (Fig. 4A). Western blot analysis revealed that the exosomes were positive for exosome markers CD9, CD63, and CD81 but negative for the ER membrane marker calnexin (Fig. 4B).

To track the delivery of exosomes, we labeled the exosomes derived from HCT116 cells with the membrane dye PKH26. As a result, the labeled exosomes were delivered to MRC-5 cells after they were co-cultured for 48 h (Fig. 4C). To prove the direct transfer of miRNAs from CRC cells to fibroblasts, we co-cultured MRC-5 cells with HCT116 cells transfected with FAM-labeled miRNAs and found that the FAM-labeled miRNAs could be transferred from CRC cells to fibroblasts via exosomes (Fig. 4D).

We further determined the levels of mature miR-146a-5p and miR-155-5p in MRC-5 cells incubated with exosomes derived from CRC cells overexpressing CXCR7 and the corresponding controls. As expected, the levels of these miRNAs were markedly enhanced in fibroblasts treated with exosomes derived from HCT116^CXCR7^ or SW620^CXCR7^ cells compared to those from control cells (Fig. 4E). These results indicate that tumor-derived exosomal miR-146a-5p and miR-155-5p can be transferred to fibroblasts. To further illustrate the effects of exosomal miRNAs on the activation of fibroblasts, we performed RT-qPCR and found that the levels of α-SMA and FAP were prominently elevated in MRC-5 cells treated with exosomes derived from CXCR7-overexpressing CRC cells compared to those from control cells (Fig. 4F). To confirm whether the effect of exosomes on fibroblast activation was due to the transfer of miR-146a-5p and miR-155-5p, MRC-5 cells were directly transfected with these miRNA mimics. When compared with the negative control (NC), the expression of α-SMA and FAP was significantly enhanced in cells transfected with these miRNA mimics (Fig. 4G, H). In contrast, the expression of α-SMA and FAP was remarkably suppressed by transfection with these miRNA inhibitors (Fig. 4I, J). More importantly, we further recapitulated the activation of fibroblasts, as shown by the upregulation of α-SMA and FAP by transfection of miR-146a-5p and miR-155-5p mimics in primary cultures of CAFs (Fig. 4K, L). Immunofluorescence analysis of α-SMA was performed to identify primary CAFs (Fig. S3). These results indicate that tumor cell-derived miR-146a-5p and miR-155-5p could be uptaken by CAFs via exosomes and promote the activation of fibroblasts.

### Exosomal miR-146a-5p and miR-155-5p promote activation of CAFs through JAK2–STAT3/NF-κB signaling

**Fig. 5:**
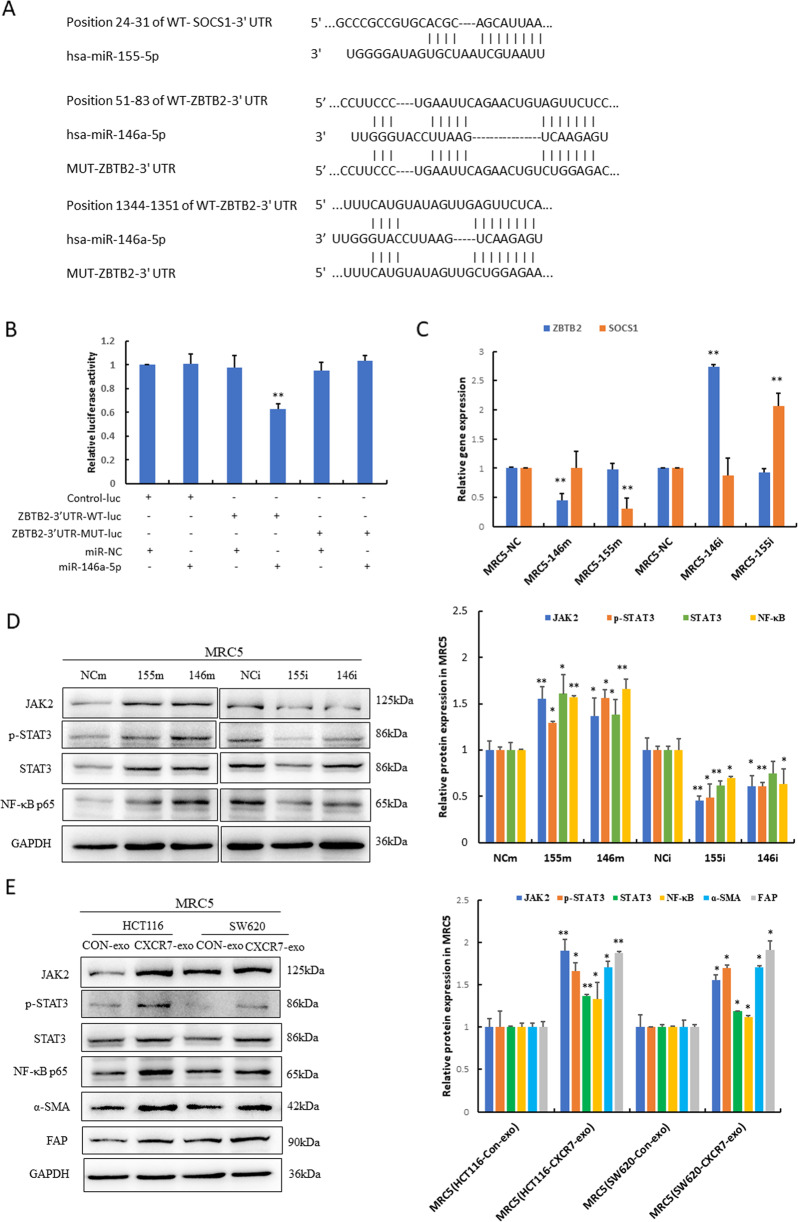
Exosomal miR-146a-5p and miR-155-5p promoted activation of CAFs through targeting ZBTB2 and SOCS1. **A** Schematic of wild type (WT) and mutant (MUT) binding sites of miR-155-5p and miR-146a-5p on 3′UTR of target gene SOCS1 and ZBTB2 respectively. **B** HCT116 cells were transfected with luciferase constructs and miR-146a-5p mimics. The comparison of luciferase activity of wild type (WT) and mutant (MUT)-ZBTB2-3′UTR constructs was performed 36 h after transfection. Data were normalized to renilla activity. **p < 0.01 vs. control group. Control-luc represents control luciferase plasmid, ZBTB-3′UTR-WT(MUT)-luc represents the wild type or mutant ZBTB2-3′UTR luciferase constructs. **C** RT-qPCR analysis of ZBTB2 and SOCS1 mRNA levels in MRC-5 cells transfected with miR-146a-5p and miR-155-5p mimics (146m, 155m) and inhibitors (146i,155i). **p < 0.01 vs. negative control (NC). **D**, **E** Western blot analysis of the expression of JAK2, p-STAT3/STAT3, NF-κB, α-SMA, and FAP in MRC-5 cells transfected with miR-155-5p and miR-146a-5p mimics (155m, 146m) and inhibitors (155i, 146i) or treated with exosomes from HCT116^CXCR7^ and SW620^CXCR7^ cells and the corresponding controls. The statistical analysis was performed. *p < 0.05 vs. control, **p < 0.01 vs. control.

To further explore the mechanism of exosomal miR-146a-5p and miR-155-5p in the activation of CAFs, we predicted the target genes of these miRNAs using Targetscan bioinformatics tools (Fig. 5A). As a result, suppressor of cytokine signaling 1 (SOCS1) and zinc finger and BTB domain containing 2 (ZBTB2) were predicted to be the target gene of miR-155-5p and miR-146a-5p, respectively. It has been reported that miR-155-5p exerts its oncogenic role by negatively regulating SOCS1 [19], which is a negative regulator of JAK–STAT3 signaling. ZBTB2 represses the transcription of RelA/p65 by inhibiting Sp1 binding to its promoter [20]. To prove that ZBTB2 is the target gene of miR-146a-5p, we performed dual-luciferase assay and the results revealed the direct binding of miR-146a-5p to the 3′-UTR of ZBTB2 to suppress the expression of ZBTB2 at the post-transcriptional level. However, miR-146a-5p did not affect the luciferase activity of the mutant 3′-UTR of ZBTB2 because the mutant seed sequence prevented miR-146a-5p from binding to the 3′-UTR of ZBTB2 (Fig. 5B).

Furthermore, we transfected these miRNAs mimics and inhibitors into MRC-5 cells. As a result, when compared with the negative control, the expression of ZBTB2 and SOCS1 was remarkably suppressed by transfection with miR-146a-5p and miR-155-5p mimics, respectively, which was substantially enhanced by the miRNA inhibitors. The results revealed that ZBTB2 and SOCS1 were potential targets of miR-146a-5p and miR-155-5p, respectively (Fig. 5C).

It is well established that JAK–STAT3/NF-κB inflammatory signaling participates in fibroblast activation, thereby contributing to the invasive/metastatic phenotype [21]. Since SOCS1 and ZBTB2 are the potential target genes of miR-155-5p and miR-146a-5p, it is likely that overexpression of these miRNAs consequently leads to the activation of JAK2–STAT3 signaling and transcriptional upregulation of NF-κB/p65. Our results showed that the levels of JAK2 and p-STAT3/STAT3 were strongly enhanced, and the expression of NF-κB/p65 was prominently increased in MRC-5 cells transfected with miR-155-5p and miR-146a-5p mimics, which were robustly impaired by these miRNA inhibitors (Fig. 5D). Consistently, exosomes derived from CXCR7-overexpressing CRC cells recapitulate the effects of overexpression of these miRNAs in MRC-5 cells. The findings revealed that activation of JAK2–STAT3 signaling and transcriptional upregulation of NF-κB/p65 were associated with the activation of CAFs (Fig. 5E).

### Activation of CAFs reciprocally promotes invasion and metastasis of CRC

**Fig. 6:**
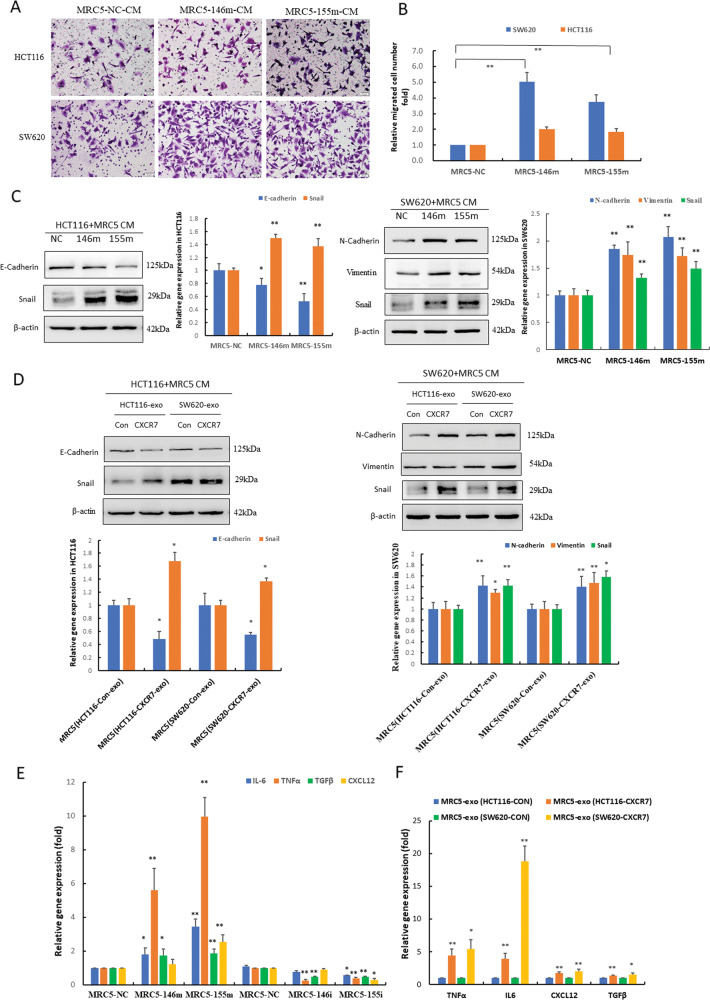
CAF activation reciprocally promoted invasion and metastasis of CRC. **A**, **B** Invasive capacity of CRC cells (HCT116 and SW620) co-cultured with CM from MRC-5 cells transfected with miR-146a-5p and miR-155-5p mimics (146m, 155m) was determined by transwell assay and statistical analysis was performed. **p < 0.01 vs. negative control (NC). **C** Western blot analysis of the expression levels of EMT-related proteins in HCT116 and SW620 cells treated with CM from MRC-5 cells transfected with miR-146a-5p and miR-155-5p mimics (146m, 155m). **D** Western blot analysis of the expression levels of EMT-related proteins in HCT116 and SW620 cells treated with CM from MRC-5 cells incubated with exosomes derived from CRC cells overexpressing CXCR7 and control. Densitometry and statistical analysis were performed. *p < 0.05, **p < 0.01 vs. control. **E** RT-qPCR analysis of IL-6, TNFα, TGFβ, and CXCL12 mRNA levels in MRC-5 cells transfected with miR-146a-5p and miR-155-5p mimics (146m, 155m) and inhibitors (146i, 155i). *p < 0.05, **p < 0.01 vs. negative control (NC). **F** RT-qPCR analysis of the mRNA levels of cytokines TNFα, IL-6, CXCL12, and TGFβ in MRC-5 cells treated with exosomes derived from HCT116^CXCR7^ and SW620^CXCR7^ cells and the corresponding controls. *p < 0.05, **p < 0.01 vs. control.

To explore whether fibroblasts activated by exosomal miR-146a-5p and miR-155-5p can promote the invasion and metastasis of CRC, we co-cultured CRC cells and miRNA-transfected fibroblasts. The results showed that CM from MRC-5 cells overexpressing miR-146a-5p and miR-155-5p significantly enhanced invasion and EMT of CRC cells compared with that in the negative control group by transwell and western blot assays (Fig. 6A–C). Notably, CM from MRC-5 cells treated with exosomes derived from CXCR7-overexpressing CRC cells also significantly promoted EMT of CRC cells (Fig. 6D).

Activation of CAFs plays an important role in matrix deposition and remodeling by releasing cytokines such as transforming growth factor-β (TGF-β), tumor necrosis factor α (TNF-α), interleukin (IL‑6), and CXCL12 [22]. To further investigate which cytokines secreted by CAFs promote EMT in CRC cells, we performed RT-qPCR analysis and found that TNF-α and IL-6 were the most enhanced cytokines in MRC-5 cells overexpressing miR-146a-5p and miR-155-5p. Consistently, TGF-β was also significantly increased, but to a less extent. Conversely, these cytokines were substantially reduced when MRC-5 cells were transfected with the miRNA inhibitors. Notably, CXCL12, secreted specifically by CAFs, was markedly enhanced in MRC-5 cells overexpressing miR-155-5p (Fig. 6E). Exosomes from CRC cells overexpressing CXCR7 recapitulated the effects of transfection with miR-146a-5p and miR-155-5p mimics, showing a pronounced increase in the levels of TNF-α and IL-6 as well as elevated TGF-β and CXCL12 to a lesser extent (Fig. 6F). Since JAK2–STAT3/NF-κB inflammatory signaling contributes to the activation of CAFs, we next explored whether blocking this inflammatory signaling could inhibit EMT of CRC cells by using STAT3 and NF-κB inhibitors. The results showed that both inhibitors Stattic and JSH-23 significantly suppressed EMT phenotype in SW620 cells treated with CM from MRC-5 cells overexpressing miR-146a-5p and miR-155-5p by differentially downregulating inflammatory cytokines (Fig. S4). The results provide support for the hypothesis that exosomal miR-146a-5p and miR-155-5p contributes to the activation of CAFs through JAK2–STAT3/NF-κB inflammatory signaling, which upregulated cytokines IL-6, TGF-β, TNF-ɑ, and CXCL12 that reciprocally promoted EMT of CRC cells.

### MiRNA-transfected CAFs promote lung metastasis of CRC in vivo

**Fig. 7:**
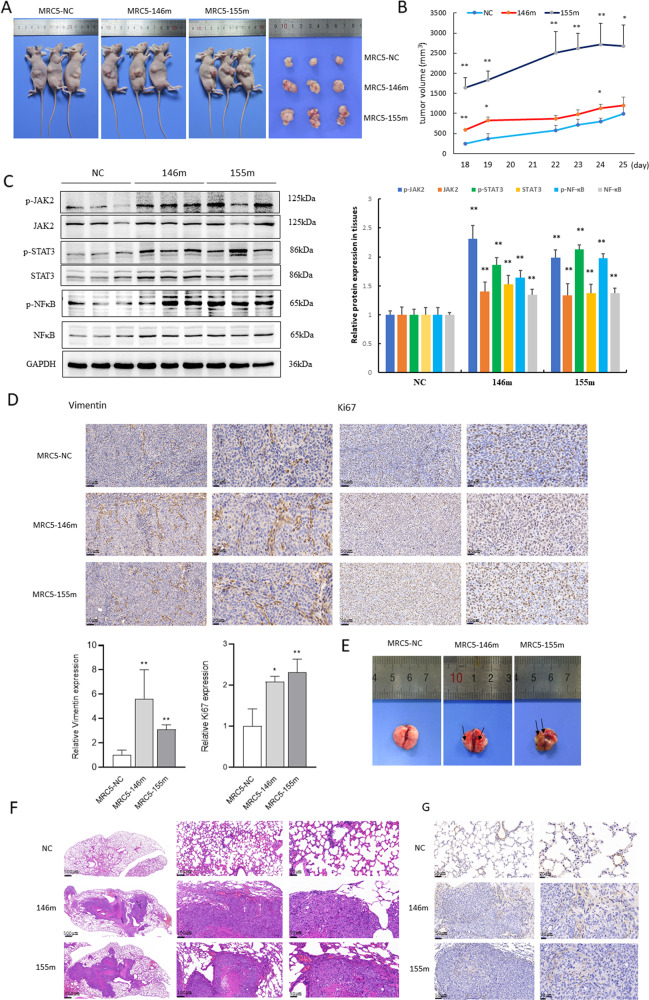
MiRNA-transfected CAFs promote tumor growth and lung metastasis of CRC in vivo. **A** Representative images show the xenograft tumors in flanks of nude mice by mixed subcutaneous injection of HCT116 cells and MRC-5 cells transfected with miR-146a-5p and miR-155-5p mimics and negative control (NC). **B** Tumor growth graphs indicated the tumor volumes at different time course (*n* = 3). *p < 0.05, **p < 0.01 vs. control. **C** Western blot analysis of the expression of p-JAK2/JAK2, p-STAT3/STAT3, p-NF-κB/NF-κB in subcutaneous xenograft tumors of nude mice as indicated and statistical analysis was performed. **p < 0.01 vs. NC. **D** IHC analysis of the expression of Vimentin and Ki67 in xenograft tumors from different groups (*n* = 3, scale bar = 50, 20 μm) and statistical analysis was performed. **E**, **F** Representative images show the lung metastasis model by injection via tail vein with HCT116 cells incubated with CM from MRC-5 cells transfected with miR-146a-5p, miR-155-5p, and control. Black arrows pointed out the metastatic nodules. HE staining of representative lung metastatic nodules were shown (*n* = 3, scale bar = 500, 100, 50 μm). **G** IHC analysis of the expression of α-SMA in lung metastatic tumors from different groups (*n* = 3, scale bar = 50, 20 μm).

We next examined whether miRNA-transfected CAFs educated CRC cells to facilitate tumor formation in vivo using tumor xenograft models in nude mice. Interestingly, we observed significantly larger tumors in mice co-injected with HCT116 and MRC-5 cells overexpressing miR-146a-5p and miR-155-5p than in the negative controls (Fig. 7A, B). To further prove these miRNAs could promote the activation of CAFs through JAK2–STAT3/NF-κB signaling in vivo, we performed western blot analysis and found marked upregulation of p-JAK2, p-STAT3, and p-NF-κB in both miR-146a-5p and miR-155-5p groups (Fig. 7C). Furthermore, IHC analysis revealed a robust increase in the expression of mesenchymal marker Vimentin and cell proliferation marker Ki67 in xenograft tumors educated with CAFs overexpressing miR-146a-5p and miR-155-5p (Fig. 7D).

**Fig. 8:**
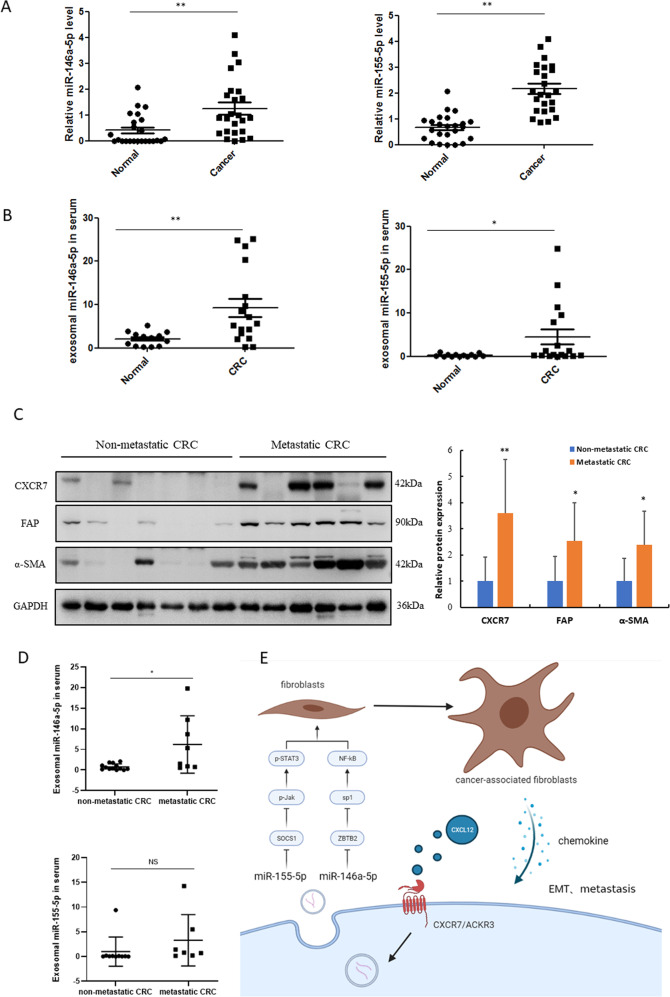
The level of miR-146a-5p and miR-155-5p was higher in CRC tissues and serum exosomes compared with that of normal counterparts. **A** Levels of miR-146a-5p and miR-155-5p were determined in colonic tissues of CRC patients and adjacent normal colon tissues (*n* = 24). **B** RT-qPCR analysis of exosomal miR-146a-5p and miR-155-5p in the serum of CRC patients (*n* = 17) and healthy volunteers (*n* = 13). *p < 0.05, **p < 0.01 vs. control. **C** Western blot analysis of expression levels of CXCR7, FAP, and α-SMA in CRC patients with (*n* = 7) and without (*n* = 10) distant metastasis. **D** RT-qPCR analysis of exosomal miR-146a-5p and miR-155-5p in the serum of non-metastatic (*n* = 10) and metastatic (*n* = 7) CRC patients. *p < 0.05. **E** The scheme depicts the intercellular communications between CXCR7-overexpressing CRC cells and activation of CAFs through exosomal miRNAs.

The potential function of CAFs in lung metastasis of CRC upon CXCL12/CXCR7 activation was further explored in vivo, and the results showed that the mice developed a larger number of lung nodules when injected with HCT116 cells educated by CM from MRC-5 overexpressing miR-146a-5p and miR-155-5p (Fig. 7E, F). IHC analysis indicated a significant upregulation of ɑ-SMA in CAFs in the lung metastatic tumors in nude mice (Fig. 7G). These results suggest that fibroblasts overexpressing miR-146a-5p and miR-155-5p promote lung metastasis of CRC, unveiling a novel mechanism for CXCL12/CXCR7-induced cancer metastasis. We also explored the clinical relevance of miR-146a-5p and miR-155-5p in CRC patients and found that the levels of these miRNAs were substantially higher in CRC tissues than in adjacent normal colonic tissues (Fig. 8A). Furthermore, the levels of serum exosomal miR-146a-5p and miR-155-5p in CRC patients were significantly higher than those in healthy controls (Fig. 8B). Importantly, the expression levels of CXCR7 and CAF activation markers FAP and α-SMA were much higher in metastatic CRC tissues (*n* = 7) than in non-metastatic CRC tissues (*n* = 10) (Fig. 8C). Notably, serum exosomal miR-146a-5p was also significantly higher in metastatic CRC tissues than in non-metastatic CRC tissues. However, we did not find significant difference in serum exosomal miR-155-5p between the two groups, possibly due to the small sample size (Fig. 8D). These results suggest that exosomal miR-146a-5p and miR-155-5p could function as potential biomarkers and contribute to CXCR7-mediated CRC metastasis by crosstalk with CAFs (Fig. 8E).

## Discussion

CXCR7, a newly identified atypical chemokine receptor, binds to CXCL12 with a higher affinity than CXCR4 [23]. Increasing evidence shows that CXCR7 is highly expressed in multiple tumors and is closely associated with cancer progression and metastasis [24]. Herein, we revealed a novel mechanism by which CXCR7 mediates CRC metastasis and found that exosomal miR-146a-5p and miR-155-5p play an important role in the crosstalk between CXCR7-overexpressing cancer cells and stromal CAFs, forming a feedback loop to facilitate CRC metastasis.

Importantly, pretreatment with AMD3100 did not significantly inhibit the upregulation of miR-146a-5p and miR-155-5p upon CXCL12 stimulation. AMD3100 is not only an inhibitor of CXCR4, but also a partial allosteric agonist of CXCR7 [25]. These findings indicate that the enhanced levels of exosomal miR-146a-5p and miR-155-5p contribute to CRC metastasis by crosstalk with CAFs, mainly through the activation of the CXCL12/CXCR7 axis. In clinical specimens of colonic tissues from both CRC patients and *villin-CXCR7* transgenic mice, we found a strong correlation between the expression of CXCR7 and the representative markers of activation of CAFs. Particularly in metastatic CRC, the expression levels of CXCR7 and α-SMA were significantly enhanced compared with those in non-metastatic tissues. These results indicate that activation of CAFs contributes to CXCL12/CXCR7-induced metastasis of CRC.

CAFs are activated fibroblasts in tumor tissues that contribute to malignant progression, invasion and metastasis by producing various types of cytokines [26]. Several agents can modulate the activation of CAFs in the TME, including inflammation signals, oxidative stress, DNA damage, and TGF-β signaling [27]. Inflammatory signaling proteins NF-κB and STAT3 are responsible for inducing and maintaining an inflammatory microenvironment and activation of CAFs through the secretion of pro-inflammatory cytokines [28]. Reciprocally, these inflammatory cytokines enhance tumor progression and metastasis [29]. In the present study, we demonstrated that miR-146a-5p and miR-155-5p promoted the phenotypic switch of CAFs by targeting ZBTB2 and SOCS1, consequently regulating JAK–STAT3 and NF-κB signaling to accelerate the formation of an inflammatory TME.

Exosomes have been shown to play a crucial role in mediating intercellular communication between cancer cells and stromal cells. The uptake of miR-21-containing exosomes into recipient fibroblasts promotes the activation of CAFs via PI3K signaling [30]. Exosomal miR-1247-3p derived from highly metastatic hepatocellular carcinoma cells activated CAFs, which in turn promoted cancer progression by secreting IL-6 and IL-8 [31]. In this study, we found that miR-146a-5p and miR-155-5p were significantly upregulated in exosomes derived from CXCR7-overexpressing CRC cells. Moreover, we proved that miR-146a-5p enhanced NF-κB expression by targeting ZBTB2. It has been reported that ZBTB2 interacts directly with transcriptional factor Sp1 and represses the NF-κB/p65 promoter by acting on the proximal GC-rich element; thus, ZBTB2 prominently repressed endogenous NF-κB/p65 expression [20]. Since SOCS1 was reported to be the target gene of miR-155-5p, and it was verified that miR-155-5p promoted the activation of CAFs via the SOCS1/JAK2/STAT3 signaling pathway. Consistently, it has been proposed that melanoma cell-secreted exosomal miR-155-5p can induce the proangiogenic switch of CAFs via the SOCS1/JAK2/STAT3 signaling pathway [32].

EMT is a biological process wherein adherent epithelial cells convert to a migratory mesenchymal phenotype, providing tumor cells with the ability to metastasize to distant organs. CRC cells exhibit great heterogeneity in the expression of EMT markers probably due to the different genetic backgrounds and metastatic capacities of CRC cells. CAFs mediate tumor-promoting inflammation and educate cancer cells into a metastatic phenotype. TNF-α and IL-6 are important inflammatory inducers of fibroblast activation in CRC through STAT3 activation, which drives CRC progression [33, 34]. CAFs release high levels of IL-6 and TNF-α in an autocrine fashion upon STAT3 activation when co-cultured with cancer cells, promoting self-renewal and metastatic potential of cancer cells [22]. TGF-β is a dominant effector that mediates the conversion of fibroblasts into CAFs. Increased TGF-β potently induces angiogenesis and promotes cancer cell invasion by stimulating EMT [35]. Interestingly, we also found CXCL12 was induced by overexpression of miR-155-5p in MRC-5 cells. CXCL12 is a chemokine that plays a key role in promoting tumor progression and orchestrating the recruitment of immune and stromal cells within the TME [36]. CXCL12 secreted by CAFs reciprocally promotes the EMT of CRC cells to form a positive feedback loop. It is well established that cancer cells can activate fibroblasts into CAFs, which in turn influences various cancer cell behaviors to promote cancer metastasis to distant organs.

In the present study, we found that activation of the CXCL12/CXCR7 axis enhanced exosomal miR-146a-5p and miR-155-5p to promote CAF activation, which alters the secretory phenotype of CAFs and promotes EMT and metastasis of CRC. Importantly, serum exosomal miR-146a-5p and miR-155-5p could serve as predictive biomarkers for CRC progression and metastasis. Thus, it is interesting to orchestrate CAF reprogramming via exosomal miRNAs as a promising therapeutic strategy for inhibiting CXCL12/CXCR7-induced metastasis of CRC.

## Supplementary information

Supplementary figures

Supplementary figure legends and methods

Supplementary tables

Data availability statement

author contribution statement

aj-checklist

Original Data File

## Data Availability

The data used in this study are available from the corresponding author upon reasonable request.
