# Supplementary figures and images for: Exosomal miR-146a-5p and miR-155-5p promote CXCL12/CXCR7-induced metastasis of colorectal cancer by crosstalk with cancer-associated fibroblasts
Source: Cell Death Dis. 2022 Apr 20;13(4):380. doi: 10.1038/s41419-022-04825-6 (PMC9021302; doi:10.1038/s41419-022-04825-6)

Fig. S1

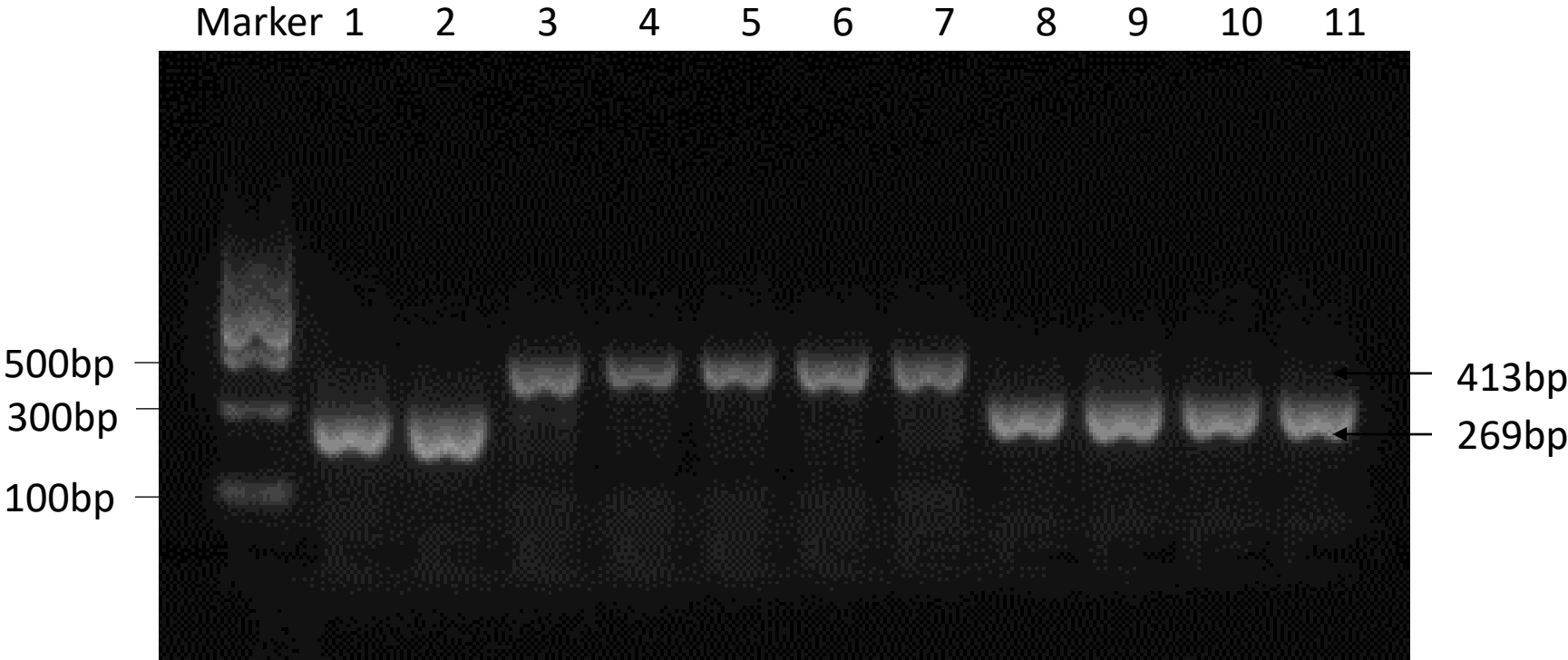

Fig. S2

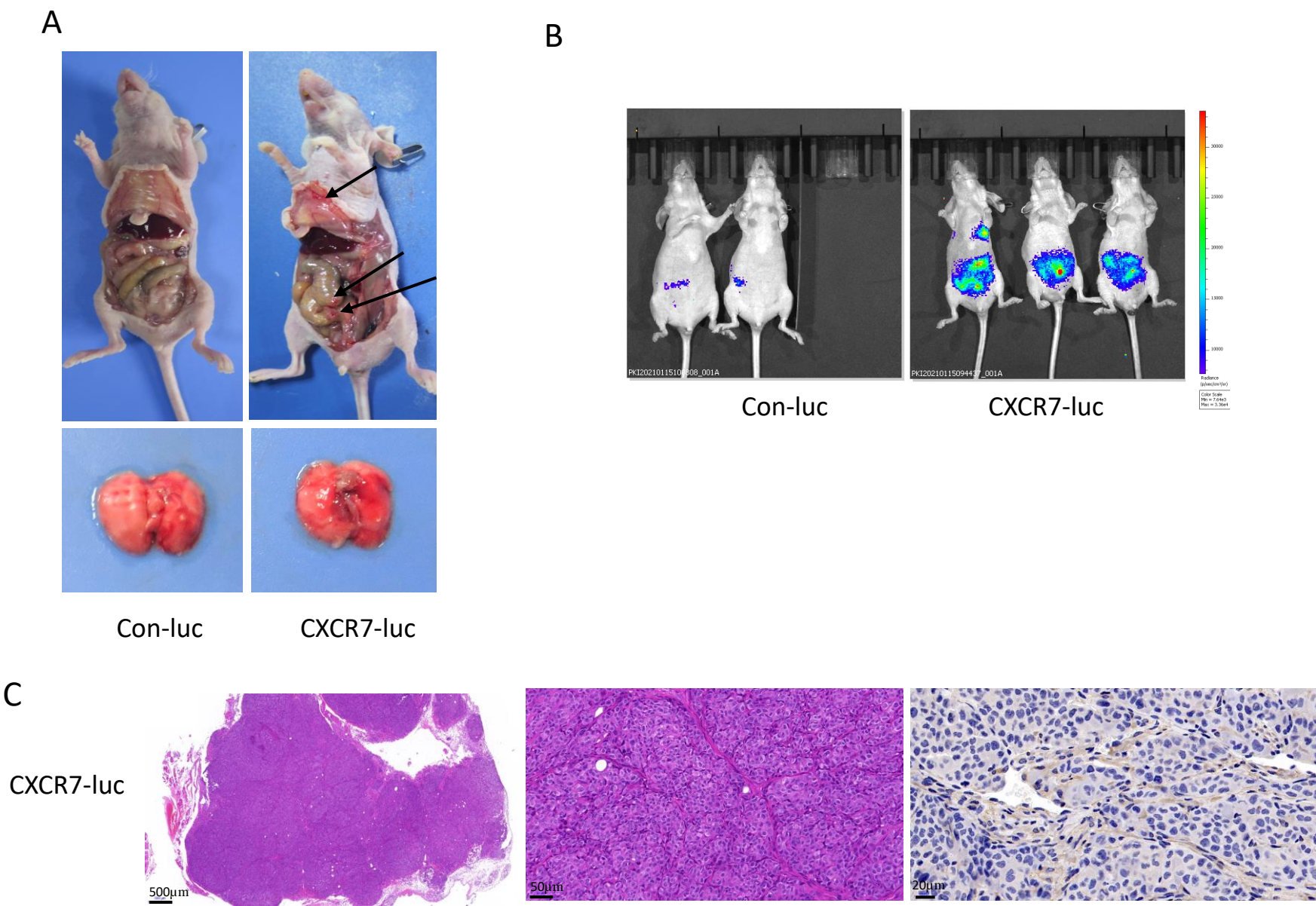

Fig. S3

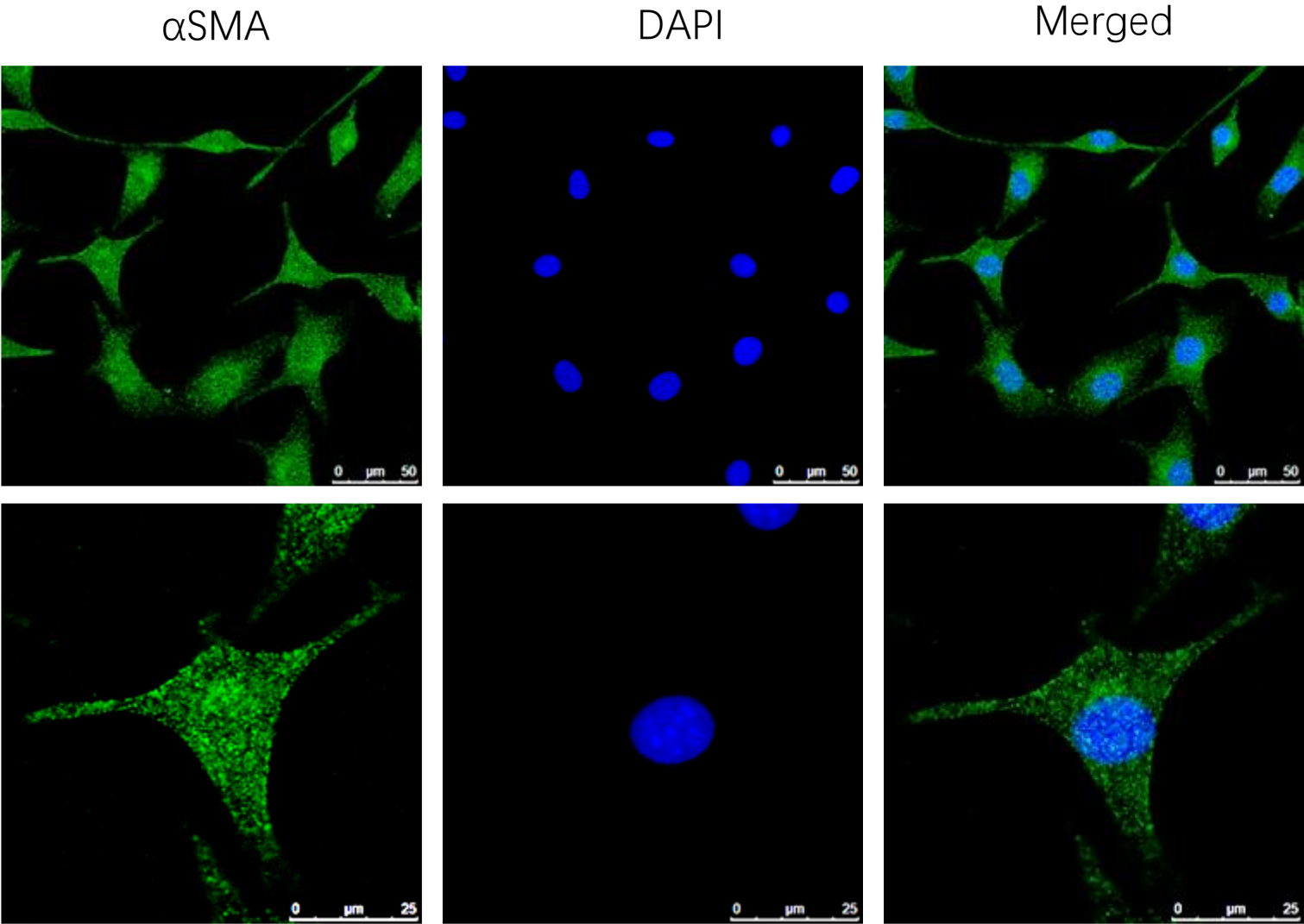

Fig. S4

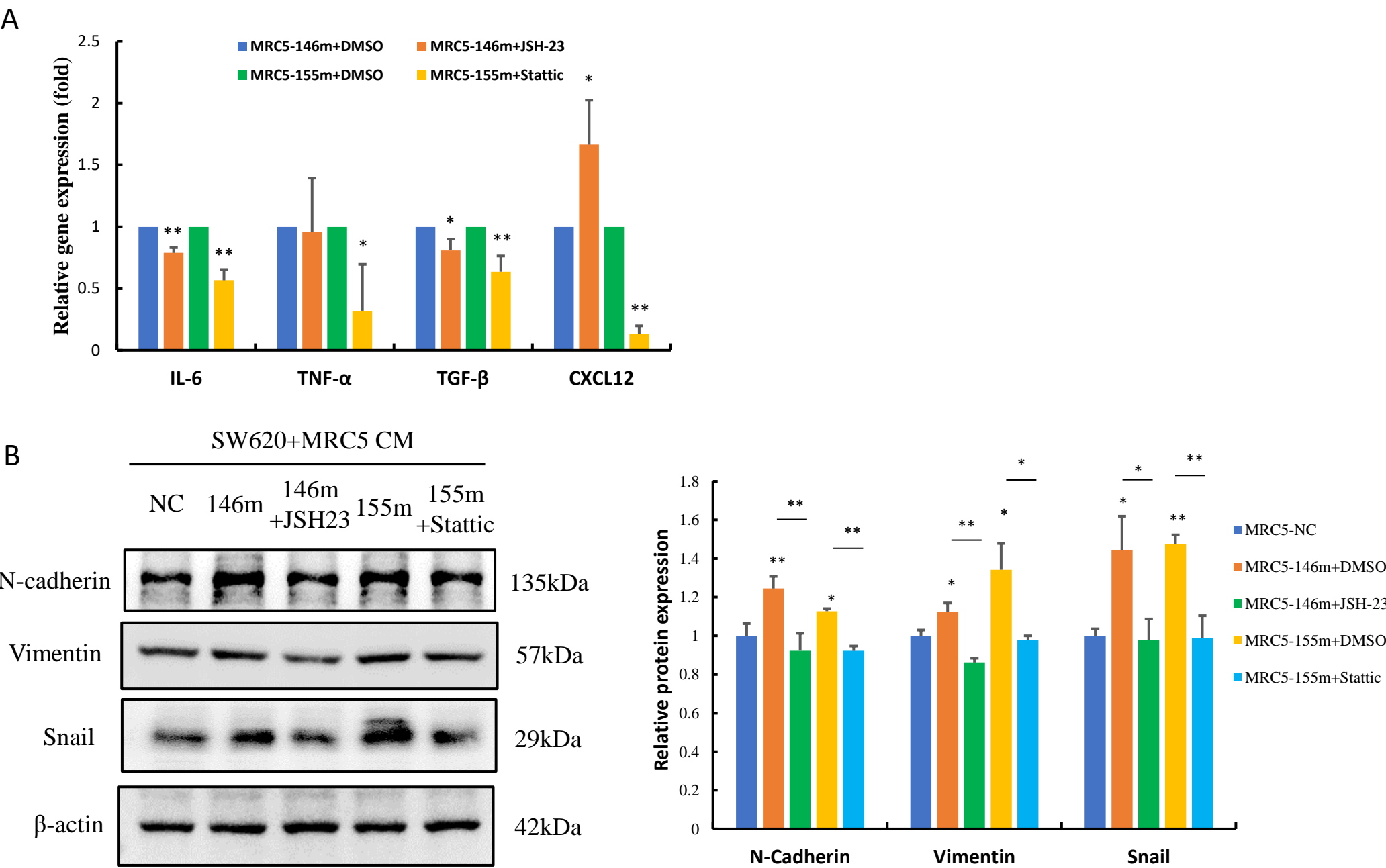

Supplement: Supplementary file 1 — Supplementary figures [file 41419_2022_4825_MOESM1_ESM.pdf]
