# Supplementary material for: Exosomal miR-146a-5p and miR-155-5p promote CXCL12/CXCR7-induced metastasis of colorectal cancer by crosstalk with cancer-associated fibroblasts
Source: Cell Death Dis. 2022 Apr 20;13(4):380. doi: 10.1038/s41419-022-04825-6 (PMC9021302; doi:10.1038/s41419-022-04825-6)
Supplement: Supplementary file 2 — Supplementary figure legends and methods [file 41419_2022_4825_MOESM2_ESM.docx]

**Supplementary Figure legends**

**Fig. S1.** PCR was performed to determine the genotype of C57BL/6J mice. Lane 1, 2, 8-11 were villin-CXCR7 transgenic mice. Lane 3-7 were wild type mice.

**Fig. S2.** (A) Representative images of lung and mesenteric tumors of nude mice injected with HCT116^Control-luc^ and HCT116^CXCR7-luc^ Cells *via* tail vein. The arrows point out the tumors. (B) Bioluminescence imaging of nude mice after injection with above cells for 3 weeks (n = 3). Note: one mouse in the control group died at early stage after injection perhaps due to embolism. (C) Representative images showing HE staining and IHC analysis of α-SMA in the metastatic tumor from nude mice injected with HCT116^CXCR7-luc^ Cells.

**Fig. S3.** Immunofluorescence was performed to identify the expression of α-SMA in CAFs. α-SMA was labeled with Alexa Fluor® 488 donkey anti-rabbit secondary antibodies, nuclei were stained with DAPI. Scale bars = 50, 25 µm.

**Fig. S4.** (A) RT-qPCR analysis of levels of IL-6, TNF-α, TGF-β, and CXCL12 mRNAs in MRC5 cells transfected with miR-146a-5p and miR-155-5p mimics and treated with or without JSH-23 (7.5 µM) and Stattic (7.5 µM) respectively for 48 h. (B) Western blot analysis of the expression levels of N-cadherin, Vimentin, and Snail in SW620 cells treated with CM from MRC-5 cells as indicated above. Statistical analysis was performed, * p < 0.05, **p < 0.01 vs. negative control (NC).

**Supplementary methods**

For the in vivo tumor metastasis studies, nude mice (5-6 weeks old) were injected with HCT116^Control^ and HCT116^CXCR7^ Cells *via* tail vein (1.5 × 10^6^ cells in 150 μl of PBS). Three weeks later, distant metastases were examined by bioluminescence imaging. To produce bioluminescence, the mice received an intraperitoneal injection of luciferin stock solution (150 mg/kg). All mice were immediately anaesthetized with 2% isoflurane and were imaged after 10 min. The images were captured using an IVIS Spectrum In Vivo Imaging System (PerkinElmer, USA).
