# Supplementary material for: Exosomal miR-146a-5p and miR-155-5p promote CXCL12/CXCR7-induced metastasis of colorectal cancer by crosstalk with cancer-associated fibroblasts
Source: Cell Death Dis. 2022 Apr 20;13(4):380. doi: 10.1038/s41419-022-04825-6 (PMC9021302; doi:10.1038/s41419-022-04825-6)
Supplement: Supplementary file 3 — Supplementary tables [file 41419_2022_4825_MOESM3_ESM.docx]

|  | Variables | Number | Percentage (%) |
| --- | --- | --- | --- |
| Age | <=70 | 12 | 50.0 |
|  | >70 | 12 | 50.0 |
| Gender | Male | 13 | 54.2 |
|  | Female | 11 | 45.8 |
| Location | Colon | 21 | 87.5 |
|  | Rectum | 3 | 12.5 |
| Depth of invasion | T_1-2_ | 4 | 16.7 |
|  | T_3-4_ | 20 | 83.3 |
| Lymph node metastasis | N_0_ | 10 | 41.7 |
|  | N_1-2_ | 14 | 58.3 |
| Distant metastasis | M_0_ | 17 | 70.8 |
|  | M_1_ | 7 | 29.2 |

Table S1. Demographic information of colorectal cancer patients

Table S2. The sequences of primers for RT-QPCR

| Gene | ﻿Primer Sequence (5′ to 3′) | Species |
| --- | --- | --- |
| α-SMA | F: CATCACCAACTGGGACGACATGGAA  R: GCATAGCCCTCATAGATGGGGACATTG | *Homo sapiens* |
| FAP | F: TGCCACCTCTGCTGTGC  R: GAAGCATTCACACTTTTCATGGT | *Homo sapiens* |
| ZBTB2 | F: GACCTCGCTGTCTCCAGAAC  R: GTGTGGATCTGGAGGTGTTC | *Homo sapiens* |
| SOCS1 | F: AGAGCTTCGACTGCCTCTTC  R: AGGGGAAGGAGCTCAGGTAG | *Homo sapiens* |
| IL-6 | F: ACTCACCTCTTCAGAACGAATTG  R: CCATCTTTGGAAGGTTCAGGTTG | *Homo sapiens* |
| TNF-α | F: CAGAGGGCCTGTACCTCATC  R: GGAAGACCCCTCCCAGATAG | *Homo sapiens* |
| CXCL12 | F: TCAGCCTGAGCTACAGATGC  R: CTTTAGCTTCGGGTCAATGC | *Homo sapiens* |
| TGFβ | F: aagtggacatcaacgggttc  R: tgcggaagtcaatgtacagc | *Homo sapiens* |
| α-SMA | F: gctgtccctctatgcctctg  R: gaaggaatagccacgctcag | Mus musculus |
| FAP | F: tcaactgtgatggcaagagc  R: taccacatcgcctggaaatc | Mus musculus |

Table S3. The upregulated miRNAs in exosomes from HCT116^CXCR7^ normalized to HCT116^Control^

| Gene ID | Type | log2 (CXCR7_exo/Control_exo) | *P* value (Control_exo/CXCR7_exo) |
| --- | --- | --- | --- |
| hsa-miR-1-3p | miRNA | 13.5265 | 0 |
| hsa-miR-122-5p | miRNA | 12.00141 | 0 |
| hsa-miR-155-5p | miRNA | 11.59805 | 0 |
| hsa-miR-133a-3p | miRNA | 10.89178 | 0 |
| hsa-miR-223-5p | miRNA | 10.73132 | 1.00E-05 |
| hsa-miR-142-5p | miRNA | 10.55075 | 2.00E-05 |
| hsa-miR-146a-5p | miRNA | 6.99058 | 0 |
| hsa-miR-223-3p | miRNA | 5.42626 | 0 |
| hsa-miR-499a-5p | miRNA | 4.45943 | 0 |
| hsa-miR-7704 | miRNA | 3.64829 | 0 |
| hsa-miR-199a-3p | miRNA | 3.58496 | 1.00E-05 |
| hsa-miR-199b-3p | miRNA | 3.58496 | 1.00E-05 |
| hsa-miR-30e-3p | miRNA | 3.52356 | 0 |
| hsa-miR-196b-5p | miRNA | 3.51211 | 0 |
| hsa-miR-12136 | miRNA | 3.3505 | 0 |
| hsa-miR-451a | miRNA | 2.93587 | 0 |
| hsa-miR-29b-3p | miRNA | 2.78136 | 0 |
| hsa-let-7g-5p | miRNA | 2.68113 | 0 |
| hsa-let-7f-5p | miRNA | 2.59483 | 0 |
| hsa-let-7i-5p | miRNA | 2.58103 | 0 |
| hsa-miR-3529-3p | miRNA | 2.54697 | 0 |
| hsa-miR-27b-3p | miRNA | 2.49881 | 0 |
| hsa-miR-98-5p | miRNA | 2.4517 | 0 |
| hsa-miR-181c-5p | miRNA | 2.32193 | 9.00E-05 |
| hsa-miR-25-3p | miRNA | 2.30812 | 0 |
| hsa-let-7b-5p | miRNA | 1.79475 | 0 |
| hsa-let-7c-5p | miRNA | 1.60039 | 0 |
| hsa-let-7a-5p | miRNA | 1.53089 | 0 |
| hsa-miR-182-5p | miRNA | 1.29768 | 5.00E-05 |
| hsa-miR-34a-5p | miRNA | 1.25798 | 0 |
| hsa-miR-24-3p | miRNA | 1.22456 | 0 |
| hsa-miR-26b-5p | miRNA | 1.22239 | 6.00E-05 |
| hsa-miR-27a-3p | miRNA | 1.0156 | 0 |
| hsa-miR-181a-5p | miRNA | 1.00431 | 0 |

Table S4. The upregulated miRNAs in exosomes from HCT116^CXCR7^ normalized to HCT116^Control^

| Gene ID | log2 (CXCR7/Control) | P value (CXCR7/Control) | log2 (CXCR7_exo/Control_exo) | P value (CXCR7_exo/CXCR7_exo) |
| --- | --- | --- | --- | --- |
| hsa-miR-155-5p | 5.12928 | 0 | 11.59805 | 0 |
| hsa-miR-146a-5p | 2.71172 | 0 | 6.99058 | 0 |
| hsa-miR-425-3p | 0.85452 | 0 | 3.90689 | 0.0014 |
| hsa-let-7a-3p | 0.69035 | 2.00E-05 | 3.08746 | 8.50E-04 |
| hsa-miR-451a | -2.36967 | 0 | 2.93587 | 0 |
| hsa-let-7g-5p | 0.40717 | 0 | 2.68113 | 0 |
| hsa-let-7f-5p | 0.46173 | 0 | 2.59483 | 0 |
| hsa-miR-3529-3p | -7.31386 | 0 | 2.54697 | 0 |
| hsa-miR-98-5p | 0.55227 | 0 | 2.4517 | 0 |
| hsa-miR-34a-5p | 0.48145 | 0 | 1.25798 | 0 |
| hsa-miR-24-3p | 0.45964 | 0 | 1.22456 | 0 |
| hsa-miR-26b-5p | 0.53963 | 0 | 1.22239 | 6.00E-05 |
| hsa-miR-27a-3p | -0.48563 | 0 | 1.0156 | 0 |
| hsa-miR-374c-3p | -16.43176 | 0 | 0.58496 | 5.90E-04 |
| hsa-miR-10a-5p | 0.59808 | 0 | -0.65486 | 8.20E-04 |
| hsa-miR-221-3p | 0.46765 | 0 | -1.07566 | 0 |
| hsa-miR-4488 | -1.64386 | 2.20E-04 | -1.16531 | 0 |
| hsa-miR-21-3p | -0.47392 | 0 | -1.51457 | 0.00193 |
| hsa-miR-19b-3p | 0.52033 | 0 | -1.77761 | 4.80E-04 |
| hsa-miR-4516 | -2.58496 | 1.00E-04 | -2.03684 | 0 |
| hsa-miR-128-3p | 0.47444 | 0 | -2.32193 | 1.00E-04 |
| hsa-miR-3184-3p | -2.52528 | 0 | -3.80735 | 9.00E-05 |
| hsa-miR-363-3p | 2.36923 | 0 | -10.3443 | 2.00E-05 |
