# Supplementary material for: Exosomal miR-146a-5p and miR-155-5p promote CXCL12/CXCR7-induced metastasis of colorectal cancer by crosstalk with cancer-associated fibroblasts
Source: Cell Death Dis. 2022 Apr 20;13(4):380. doi: 10.1038/s41419-022-04825-6 (PMC9021302; doi:10.1038/s41419-022-04825-6)
Supplement: Supplementary file 4 — Data availability statement [file 41419_2022_4825_MOESM4_ESM.docx]

**Data Availability**

The data used in this study are available from the corresponding author upon reasonable request.
