# Supplementary material for: Exosomal miR-146a-5p and miR-155-5p promote CXCL12/CXCR7-induced metastasis of colorectal cancer by crosstalk with cancer-associated fibroblasts
Source: Cell Death Dis. 2022 Apr 20;13(4):380. doi: 10.1038/s41419-022-04825-6 (PMC9021302; doi:10.1038/s41419-022-04825-6)
Supplement: Supplementary file 5 — author contribution statement [file 41419_2022_4825_MOESM5_ESM.docx]

**Authors’ contribution statement**

XY designed the research. DW, XW, Y-J S, MS, Y-Q S, XL performed the experiments and analyzed data. XY, DW drafted the manuscript, SC and XQ revised the manuscript. All authors read and approved the final manuscript.
