# Supplementary material for: Exosomal miR-146a-5p and miR-155-5p promote CXCL12/CXCR7-induced metastasis of colorectal cancer by crosstalk with cancer-associated fibroblasts
Source: Cell Death Dis. 2022 Apr 20;13(4):380. doi: 10.1038/s41419-022-04825-6 (PMC9021302; doi:10.1038/s41419-022-04825-6)
Supplement: Supplementary file 7 — Original Data File [file 41419_2022_4825_MOESM7_ESM.pdf]

Figure 2E

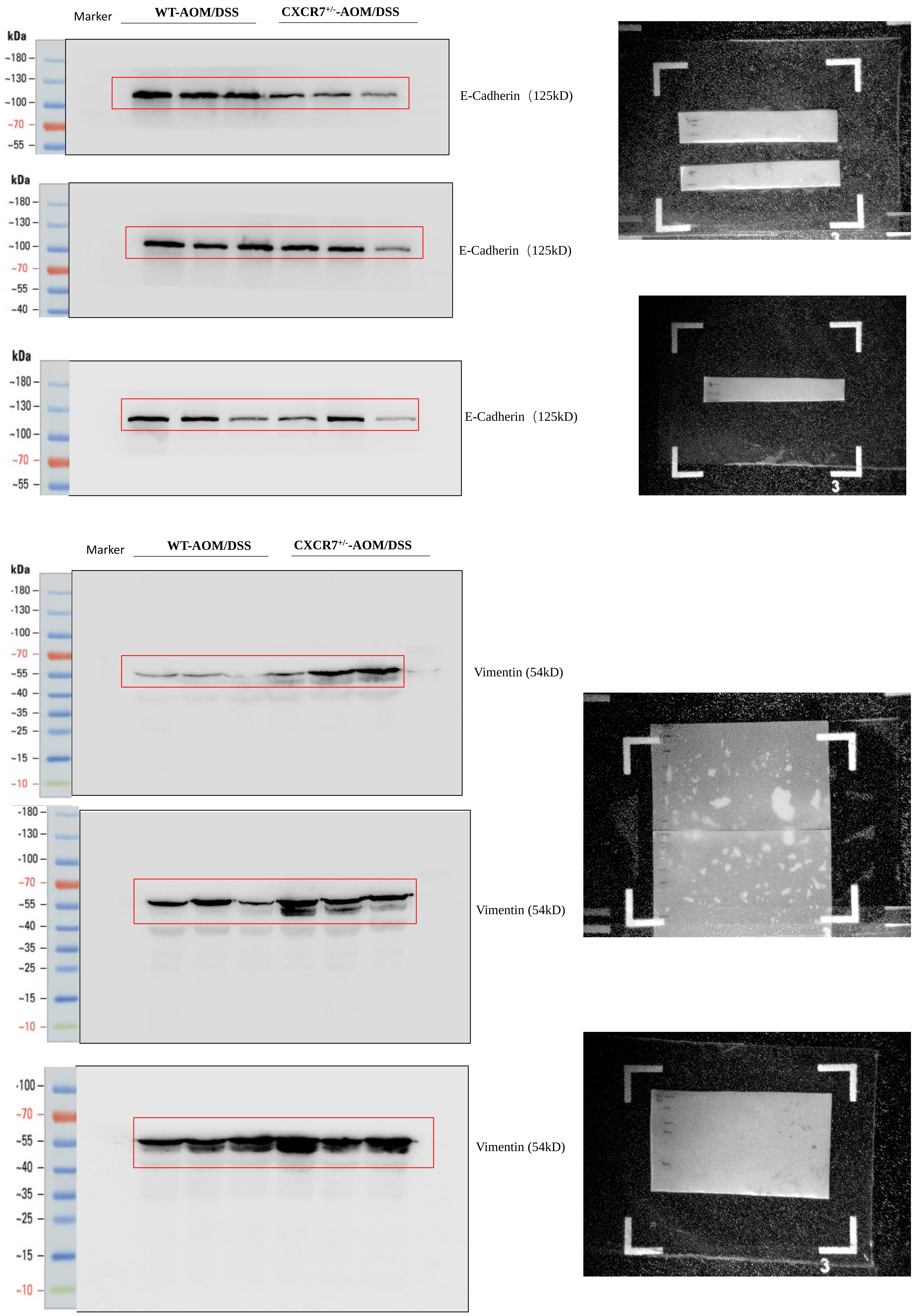

Marker
WT-AOM/DSS
CXCR7+/--AOM/DSS
kDa
~180
~130
~100
~70
~55
~40
E-Cadherin（125kD)
E-Cadherin（125kD)
E-Cadherin（125kD)
Marker
WT-AOM/DSS
CXCR7+/--AOM/DSS
kDa
~180
~130
~100
~70
~55
~40
~35
~25
~15
~10
Vimentin (54kD)
Vimentin (54kD)
Vimentin (54kD)
3

Figure 2E

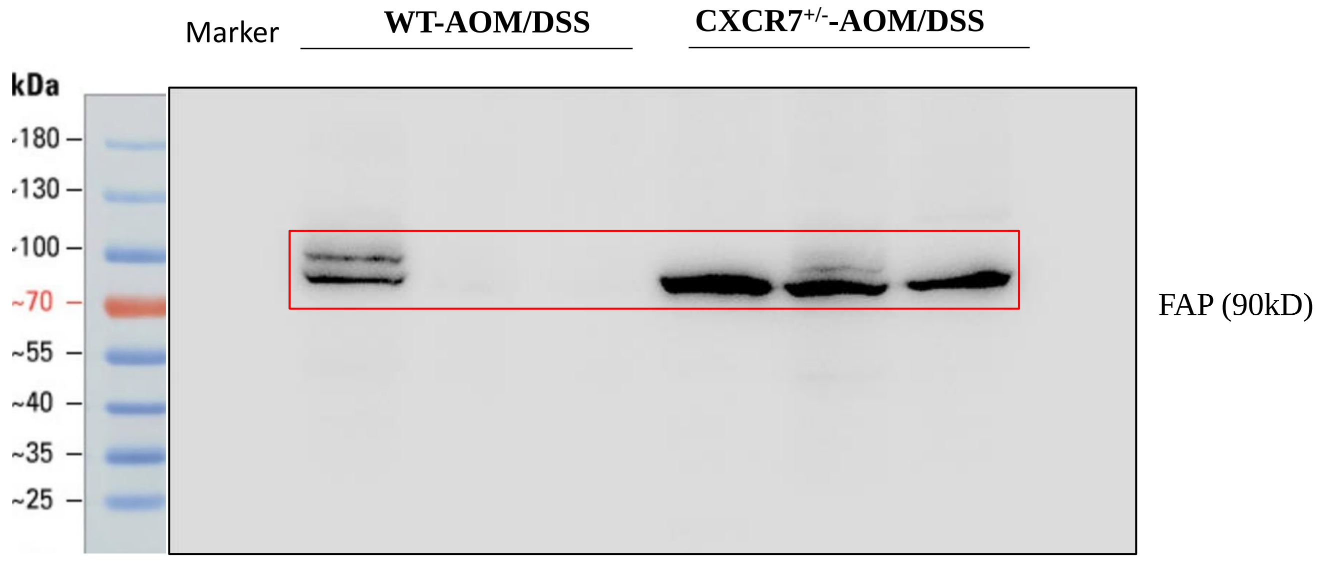
Marker
WT-AOM/DSS
CXCR7+/--AOM/DSS
kDa
180
130
100
70
55
40
35
25
FAP (90kD)

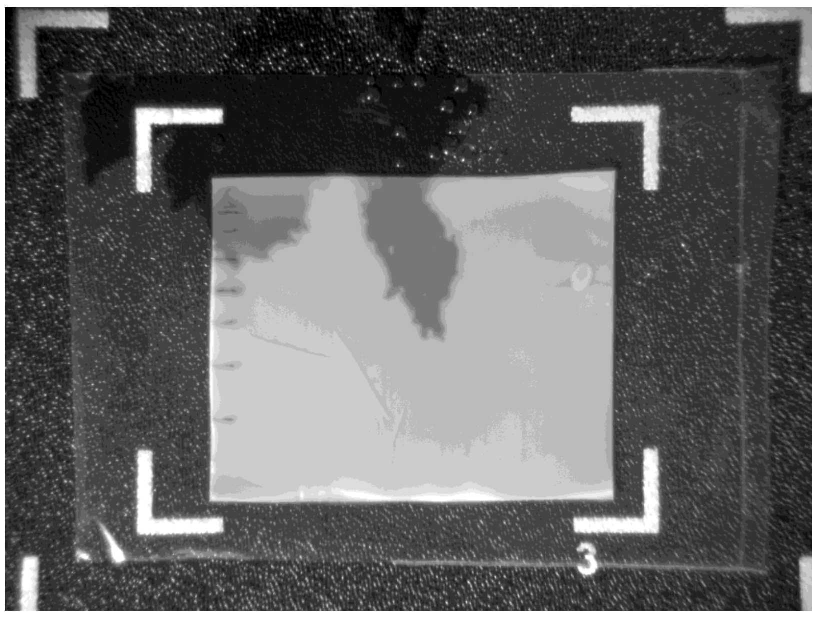
3

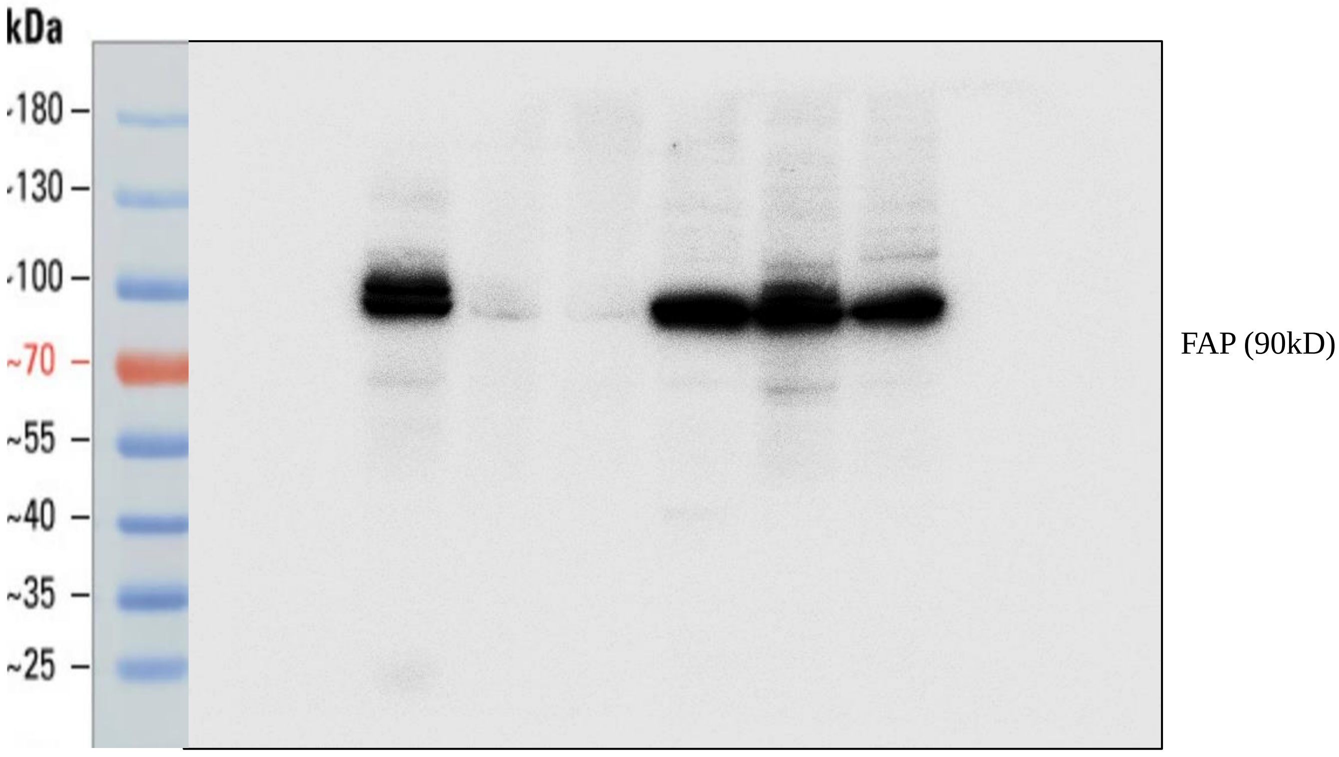
kDa
180
130
100
70
55
40
35
25
FAP (90kD)

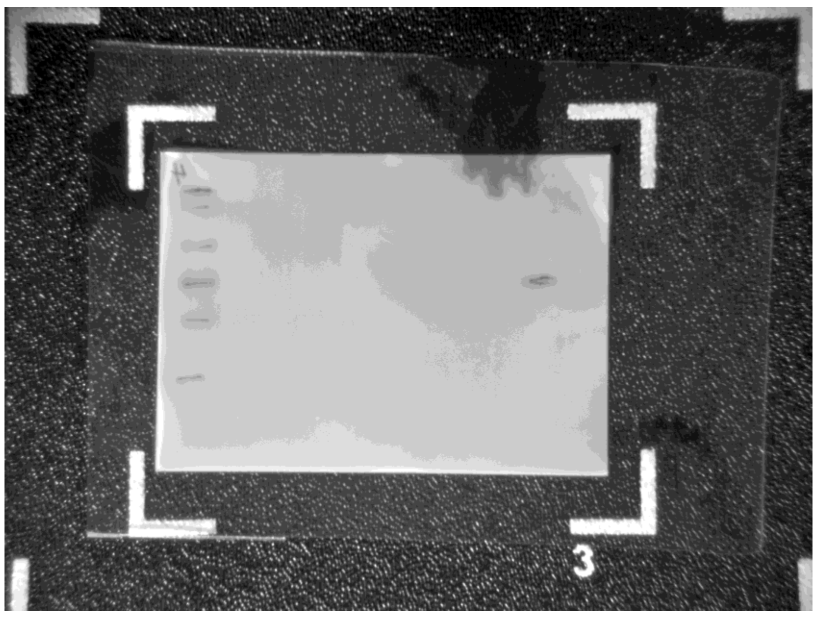
3

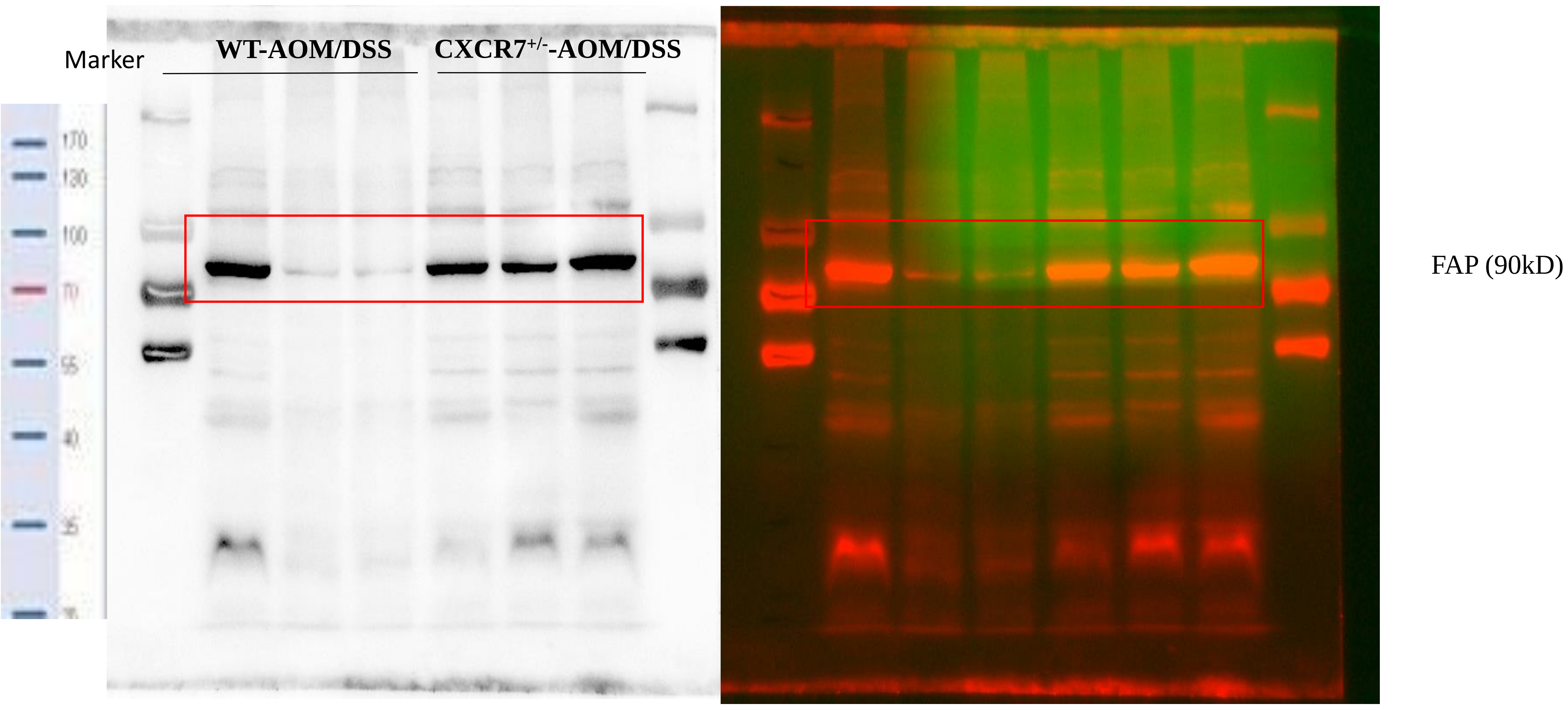
Marker
WT-AOM/DSS
CXCR7+/--AOM/DSS
FAP (90kD)

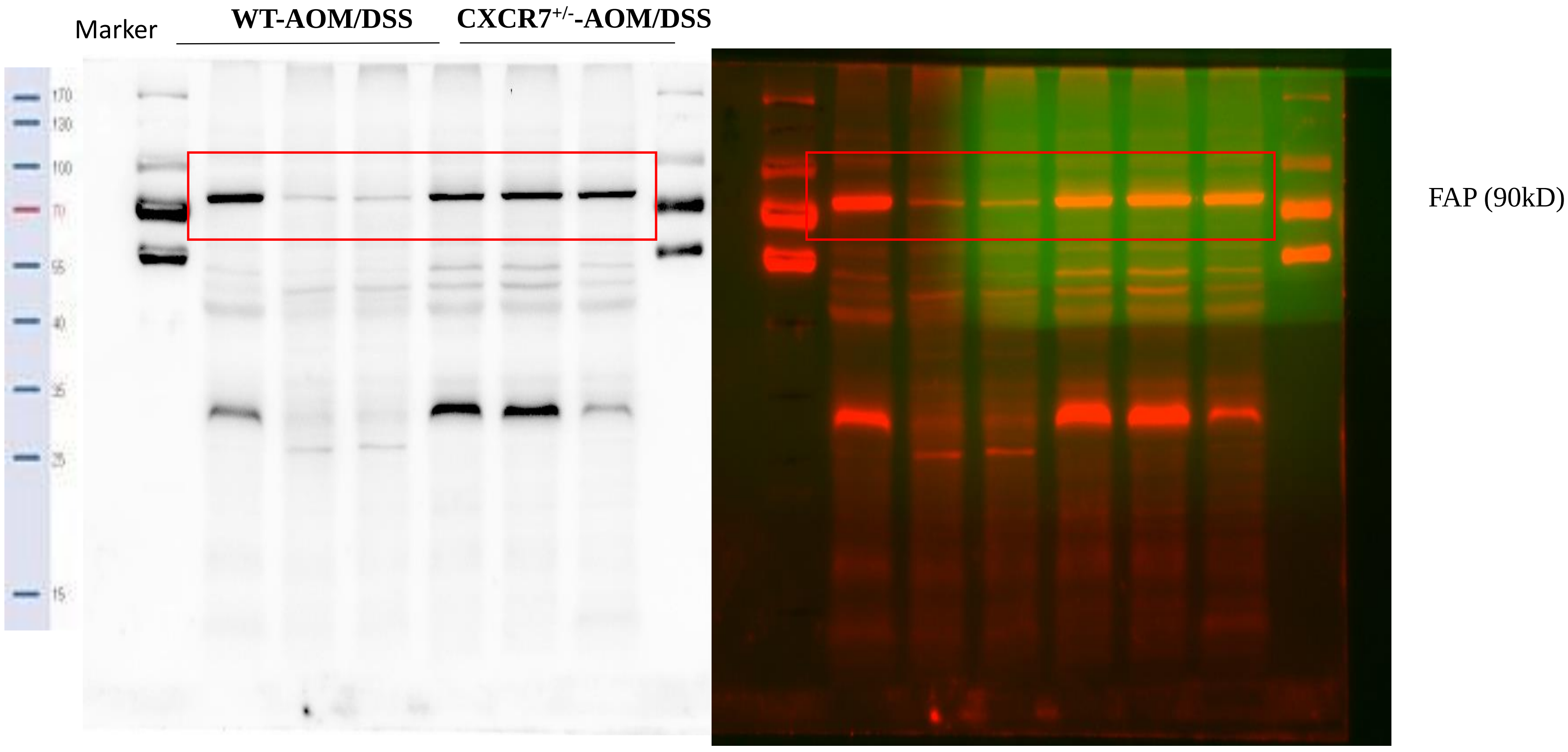
Marker
WT-AOM/DSS
CXCR7+/--AOM/DSS
FAP (90kD)

Figure 2E

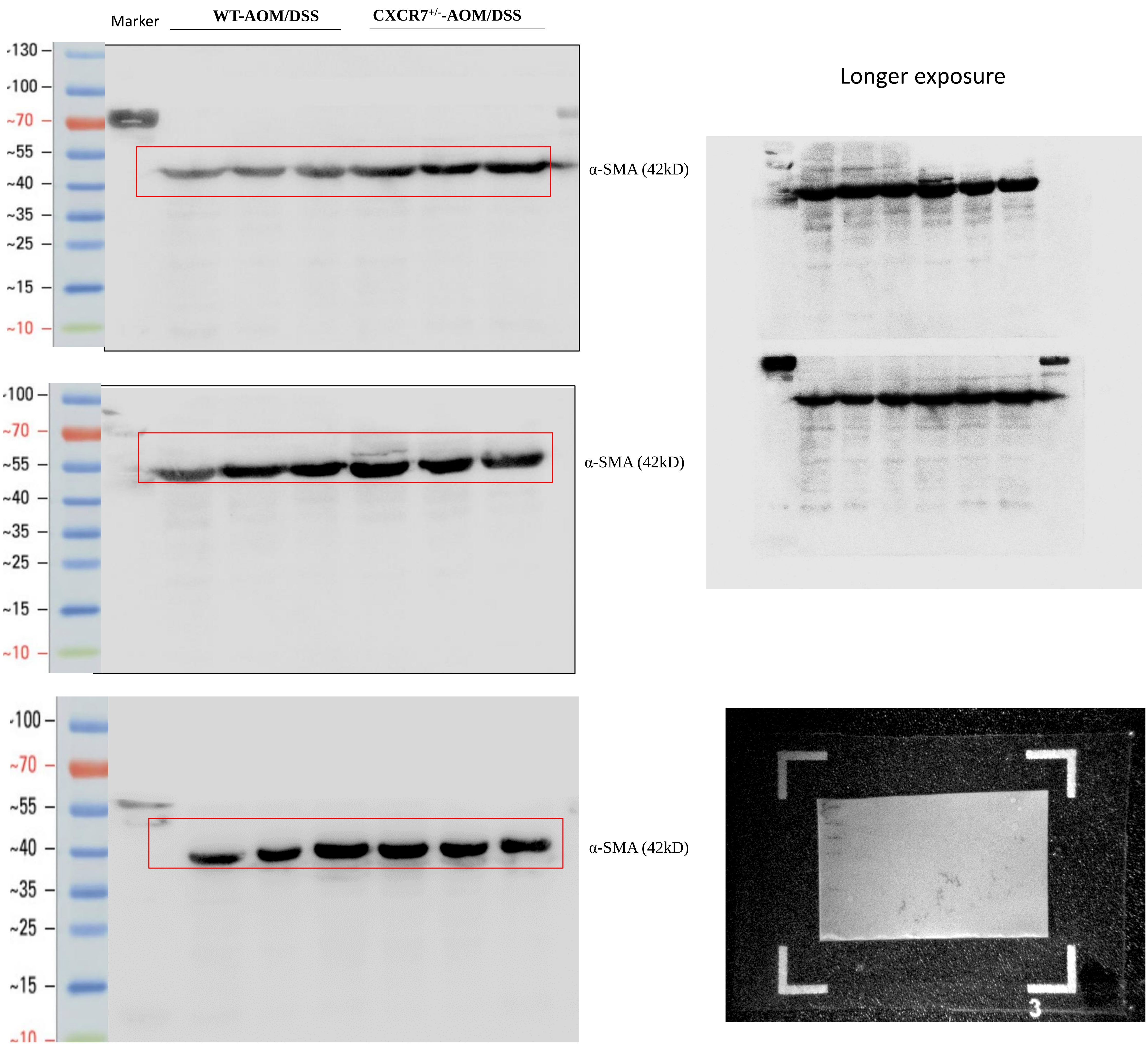

Marker
WT-AOM/DSS
CXCR7+/--AOM/DSS
·130
·100
~70
~55
~40
~35
~25
~15
~10
α-SMA (42kD)
·100
~70
~55
~40
~35
~25
~15
~10
α-SMA (42kD)
·100
~70
~55
~40
~35
~25
~15
α-SMA (42kD)
Longer exposure

Figure 2E

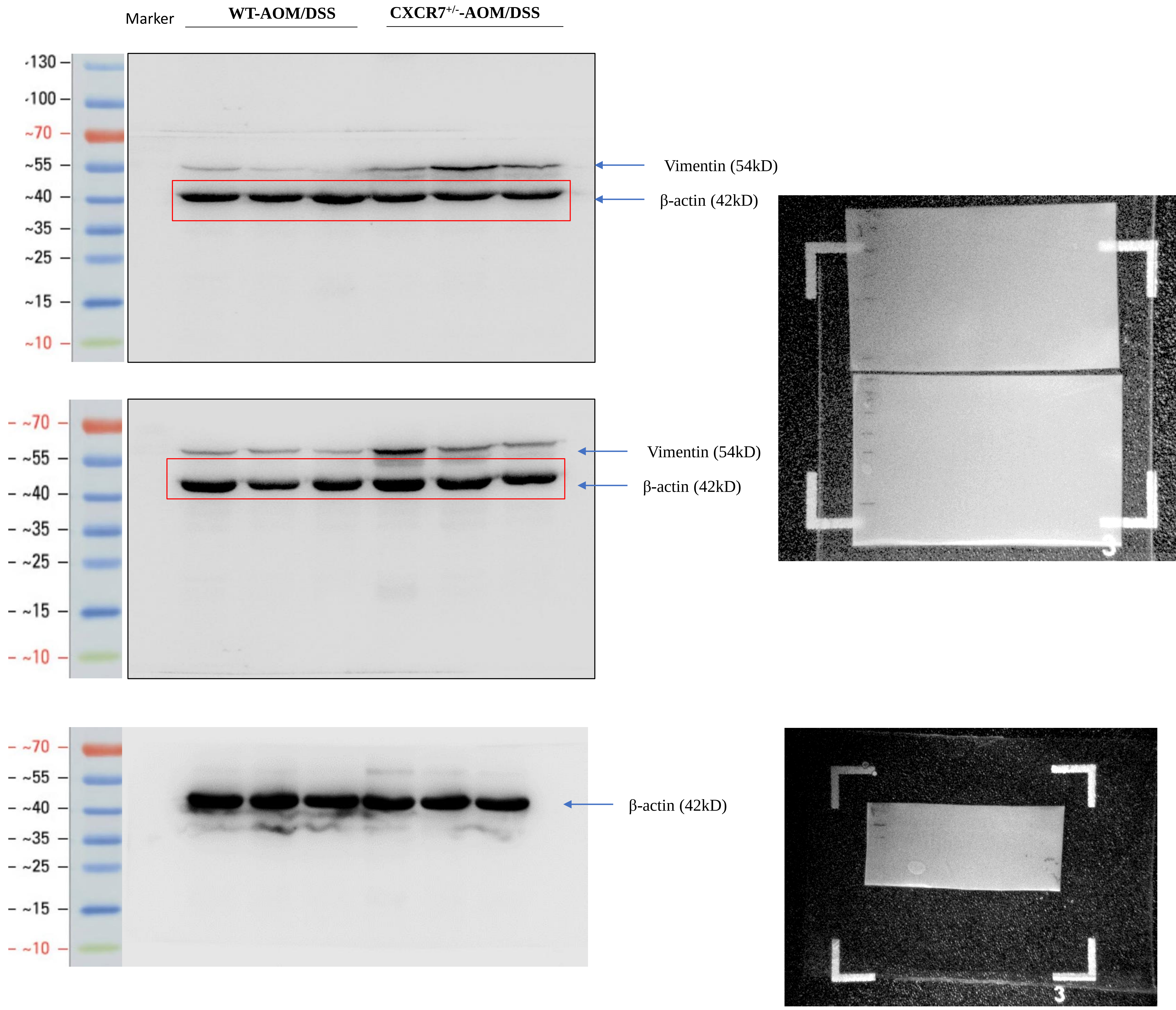

This membrane was first used to detect the band of vimentin, then used to detect internal control β-actin.

Figure 2E

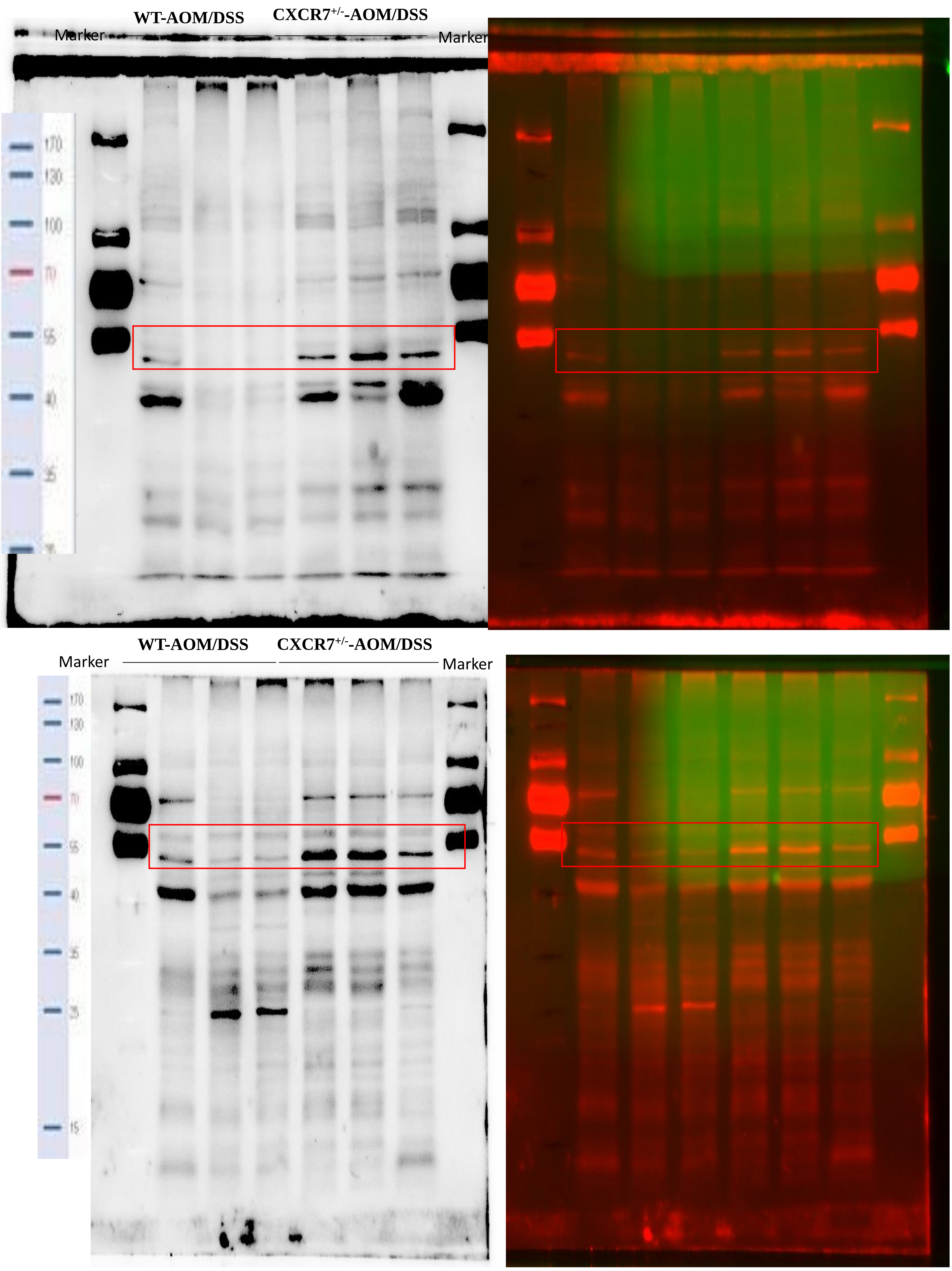

CXCR7(42kD)

CXCR7(42kD)

Figure 3A

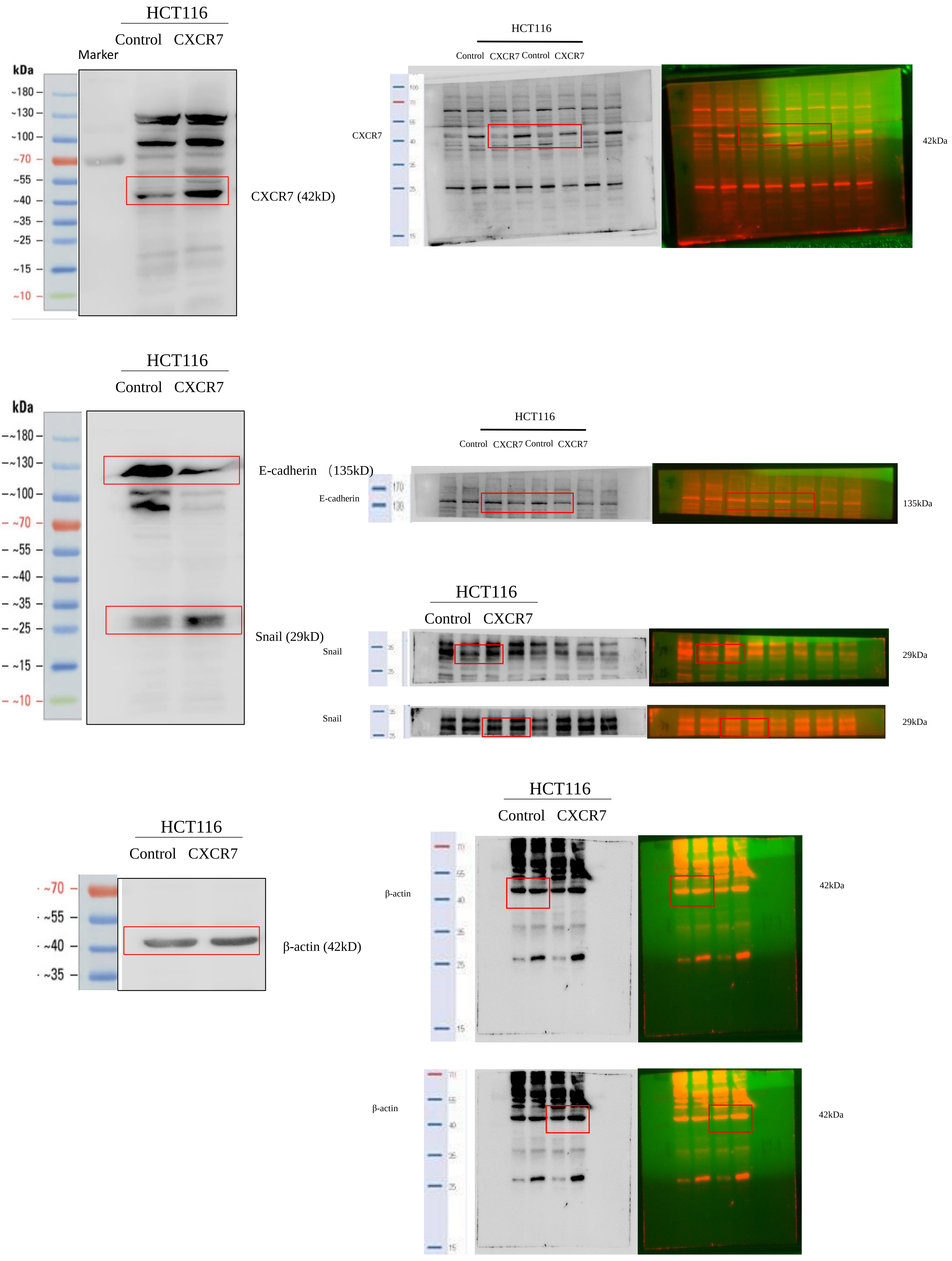

HCT116
Control CXCR7
Marker
kDa
~180
~130
~100
~70
~55
~40
~35
~25
~15
~10
CXCR7 (42kD)
HCT116
Control CXCR7 Control CXCR7
CXCR7
42kDa
HCT116
Control CXCR7
kDa
~180
~130
~100
~70
~55
~40
~35
~25
~15
~10
E-cadherin （135kD)
Snail (29kD)
HCT116
Control CXCR7 Control CXCR7
E-cadherin
135kDa
HCT116
Control CXCR7
Snail
29kDa
Snail
29kDa
HCT116
Control CXCR7
~70
~55
~40
~35
β-actin (42kD)
HCT116
Control CXCR7
β-actin
42kDa
β-actin
42kDa

Figure 4B

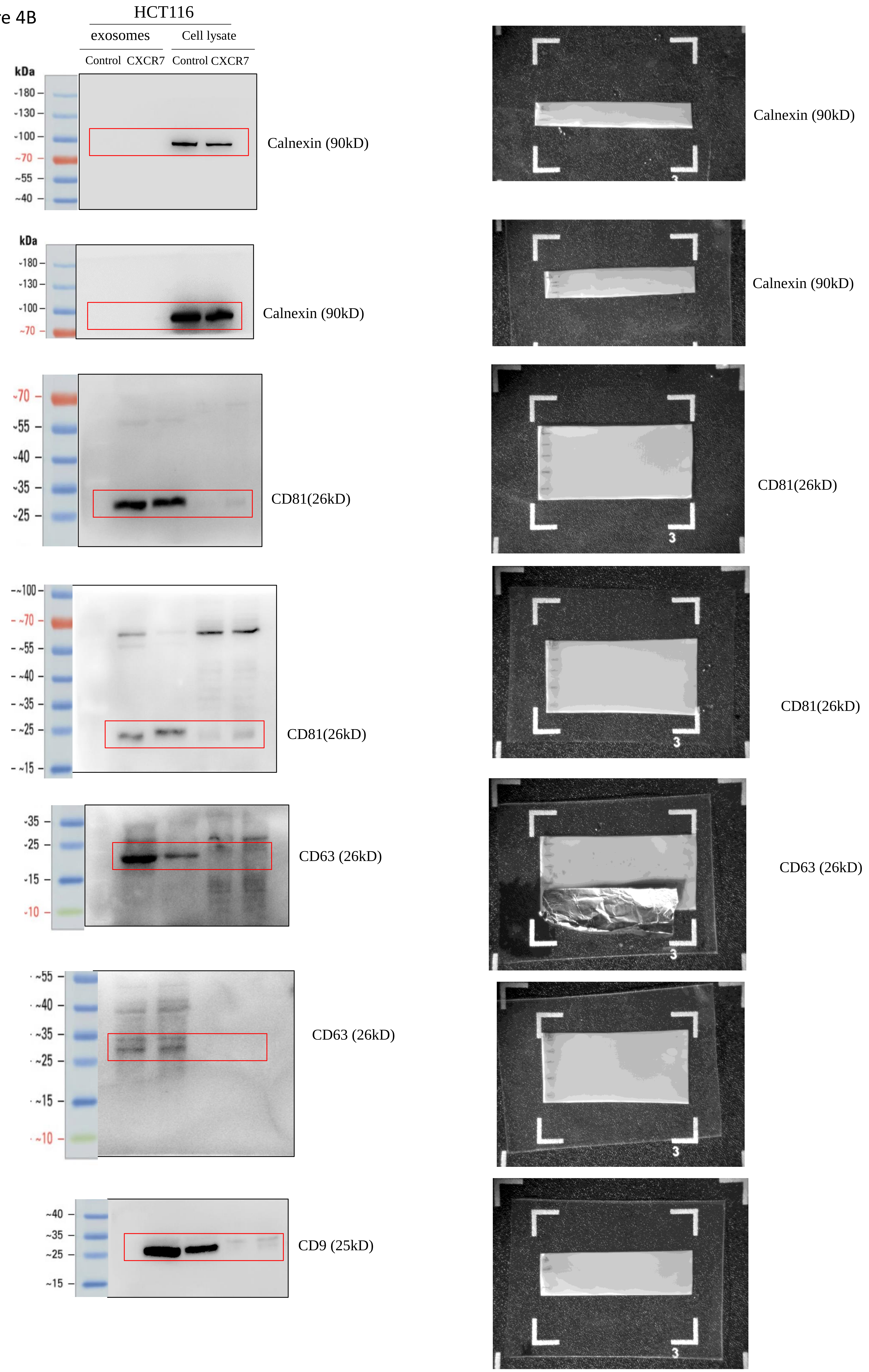
HCT116
exosomes
Cell lysate
Control CXCR7 Control CXCR7
kDa
180
130
100
70
55
40
Calnexin (90kD)
kDa
180
130
100
70
Calnexin (90kD)
70
55
40
35
25
CD81(26kD)
100
70
55
40
35
25
15
CD81(26kD)
35
25
15
10
CD63 (26kD)
55
40
35
25
15
10
CD63 (26kD)
40
35
25
15
CD9 (25kD)
Calnexin (90kD)
Calnexin (90kD)
CD81(26kD)
CD81(26kD)
CD63 (26kD)
CD9 (25kD)

Figure 4H

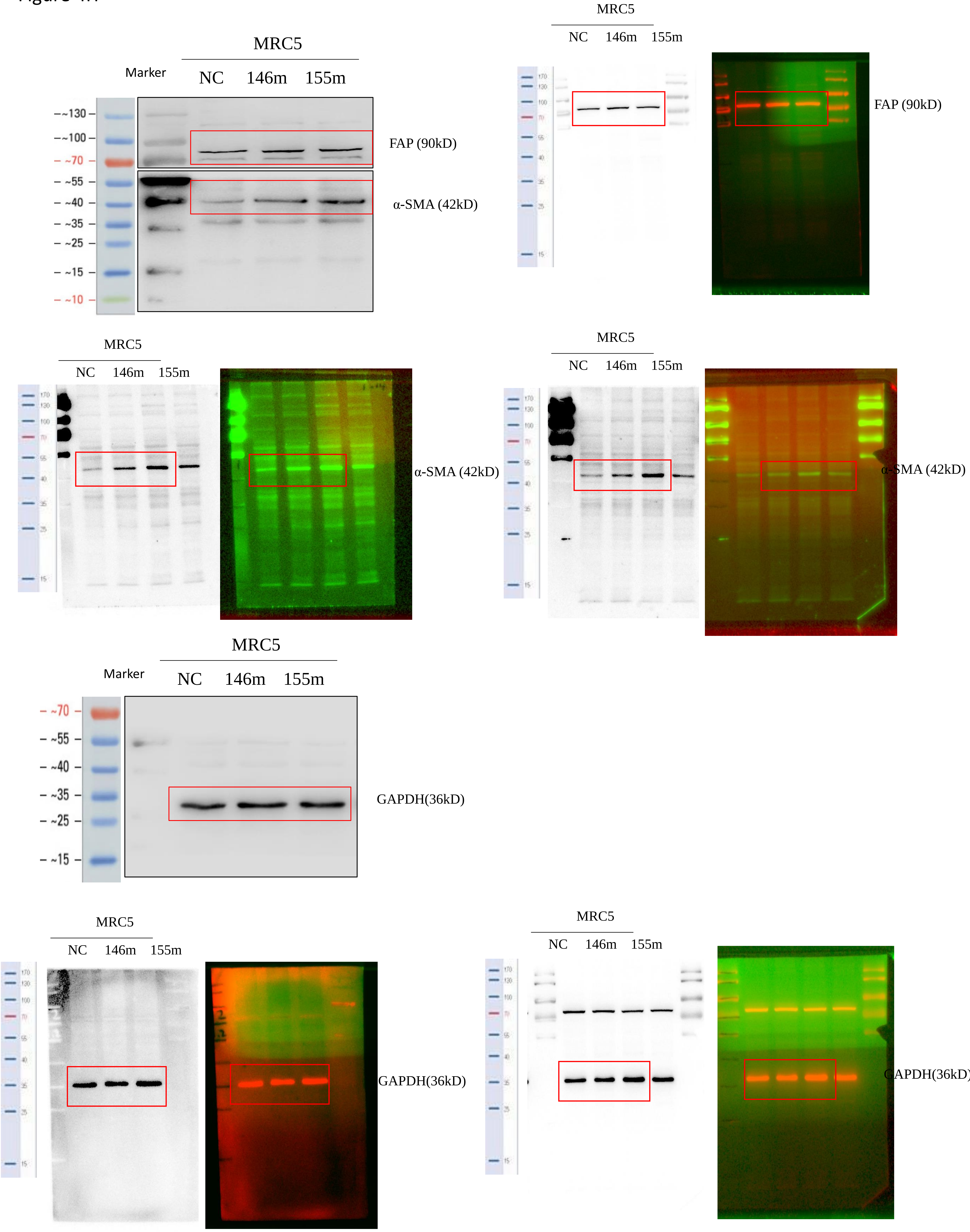
MRC5
Marker
NC
146m
155m
~130
~100
~70
~55
~40
~35
~25
~15
~10
FAP (90kD)
α-SMA (42kD)
GAPDH(36kD)

Figure 4J

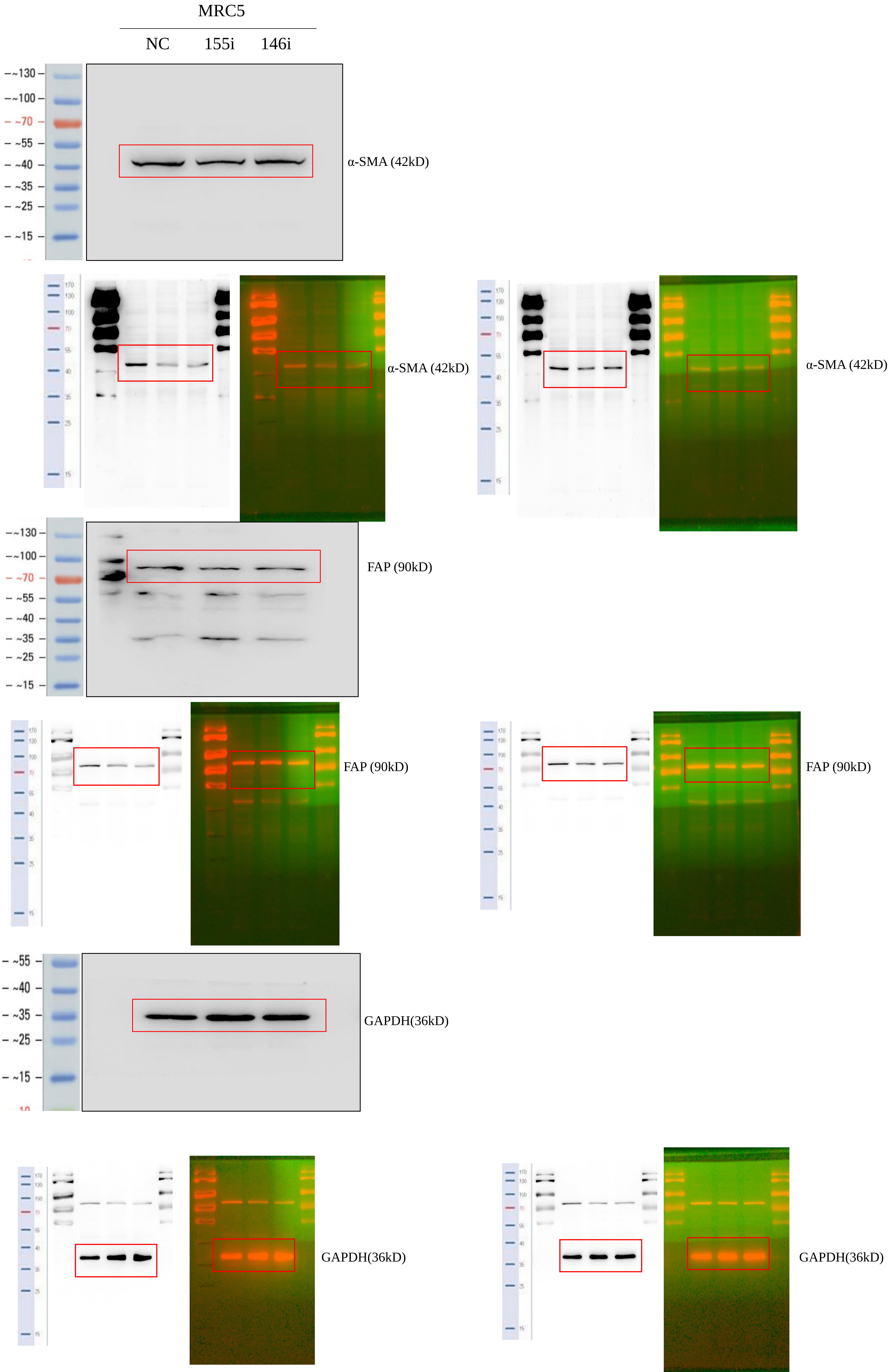
MRC5
NC
155i
146i
~130
~100
~70
~55
~40
~35
~25
~15
α-SMA (42kD)
α-SMA (42kD)
α-SMA (42kD)
FAP (90kD)
FAP (90kD)
FAP (90kD)
GAPDH(36kD)
GAPDH(36kD)
GAPDH(36kD)

Figure 4L

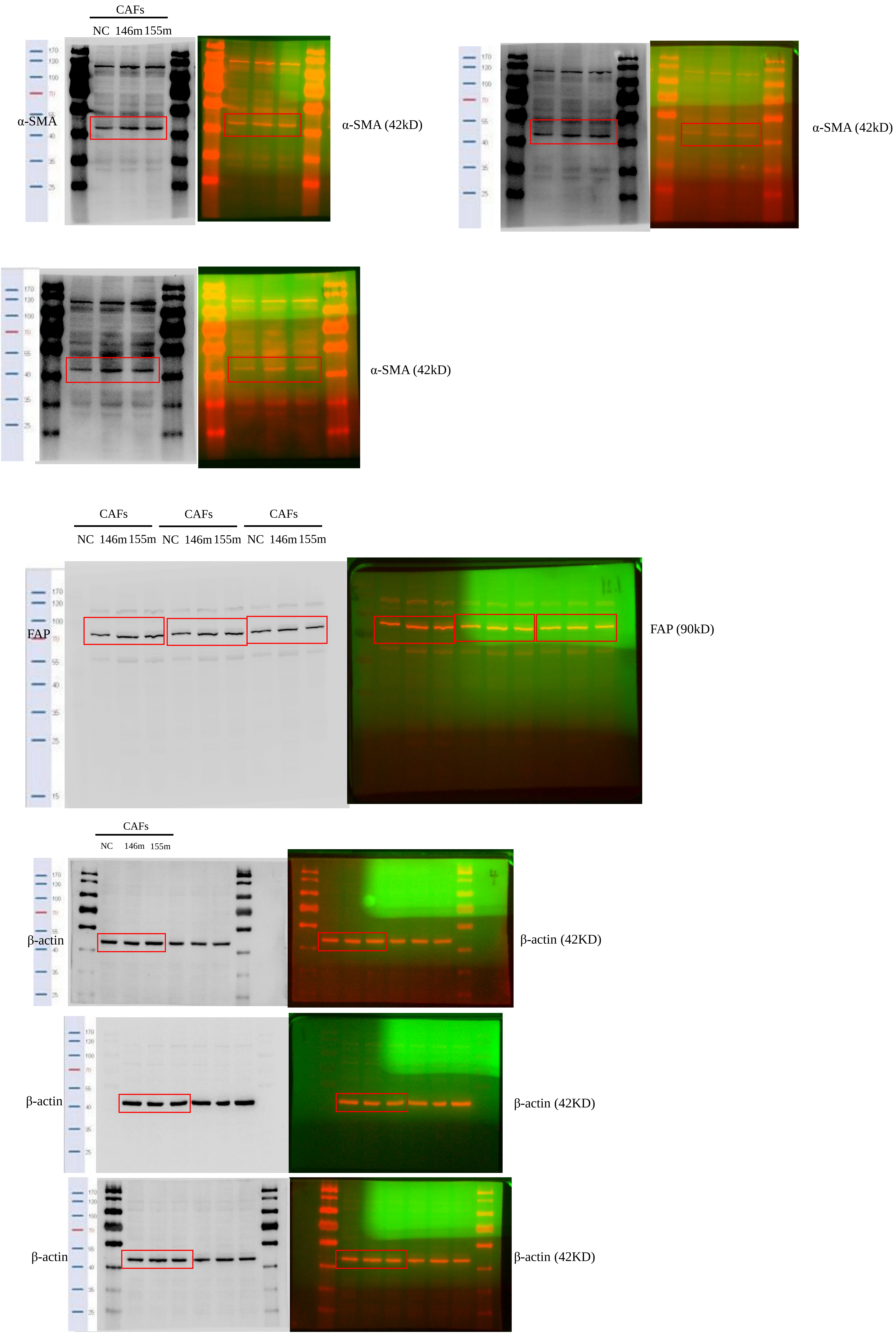
CAFs
NC 146m 155m
α-SMA
α-SMA (42kD)
α-SMA (42kD)
α-SMA (42kD)
CAFs
CAFs
CAFs
NC 146m 155m NC 146m 155m NC 146m 155m
FAP
FAP (90kD)
CAFs
NC 146m 155m
β-actin
β-actin (42KD)
β-actin
β-actin (42KD)
β-actin
β-actin (42KD)

Figure 5D

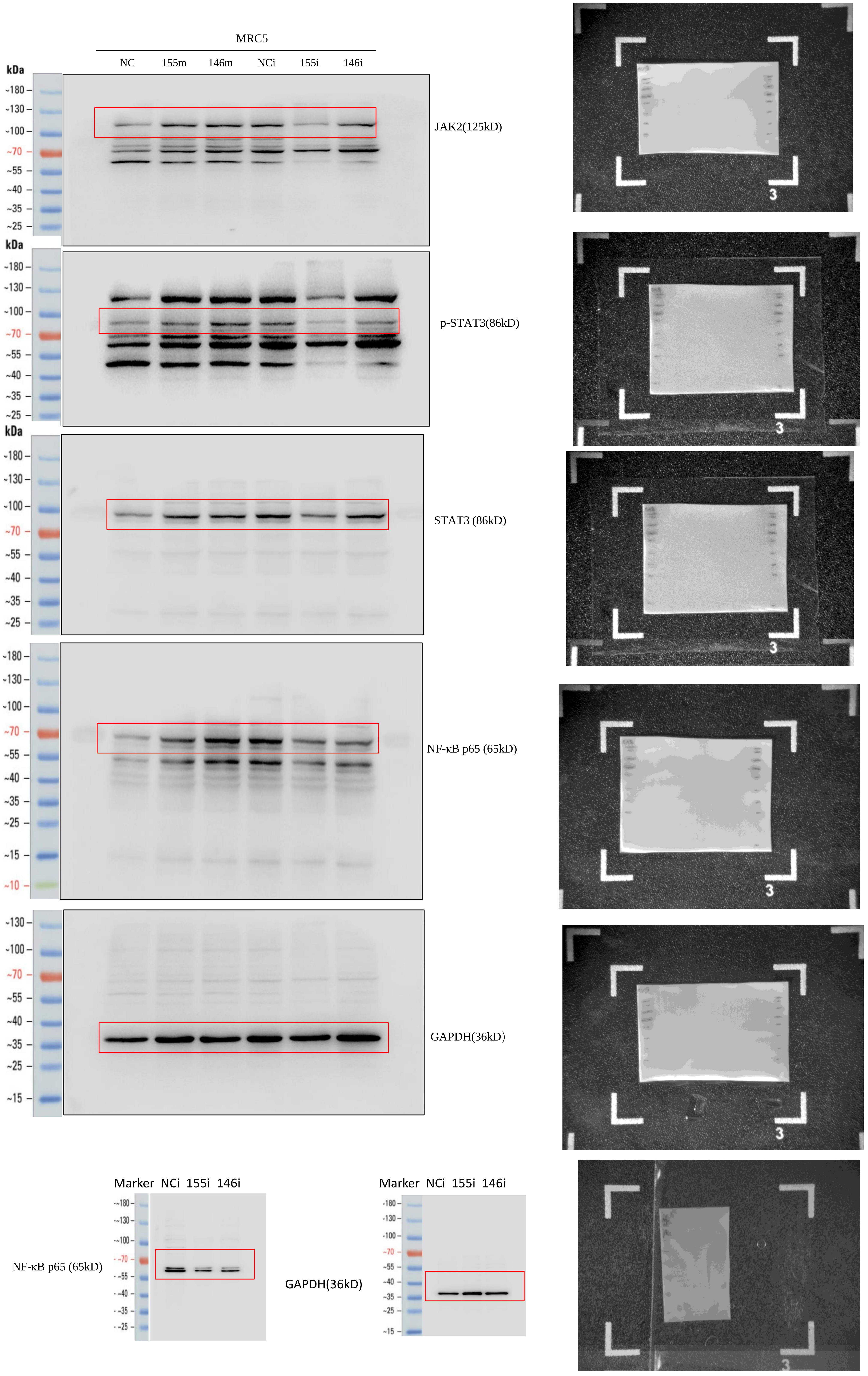

MRC5
NC
155m
146m
NCi
155i
146i
kDa
~180
~130
~100
~70
~55
~40
~35
~25
~15
~10
JAK2(125kD)
p-STAT3(86kD)
STAT3 (86kD)
NF-κB p65 (65kD)
GAPDH(36kD)
Marker NCi 155i 146i
NF-κB p65 (65kD)
Marker NCi 155i 146i
GAPDH(36kD)
3

Figure 5D

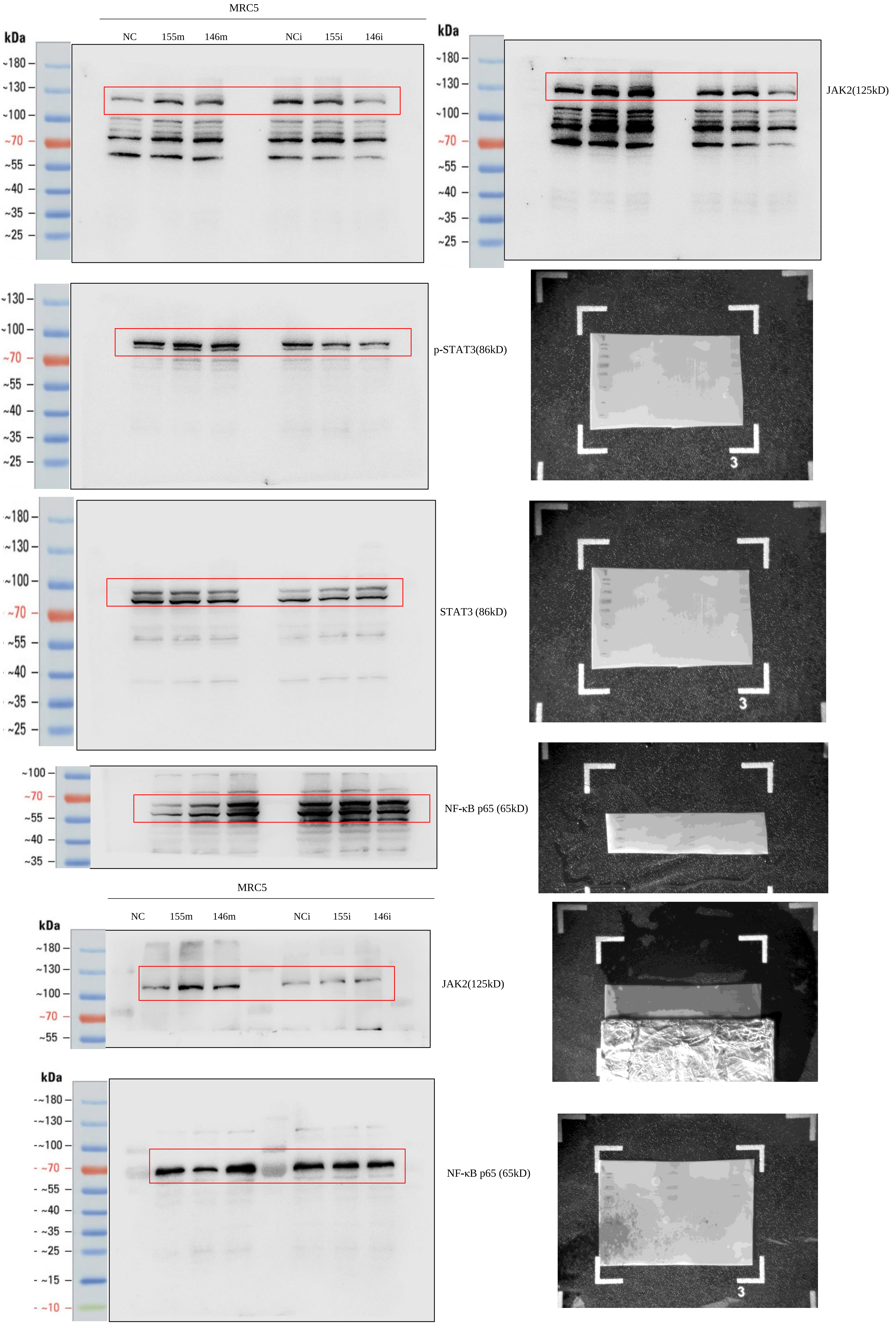

MRC5
NC
155m
146m
NCi
155i
146i
kDa
~180
~130
~100
~70
~55
~40
~35
~25
~15
~10
JAK2(125kD)
p-STAT3(86kD)
STAT3 (86kD)
NF-κB p65 (65kD)
3

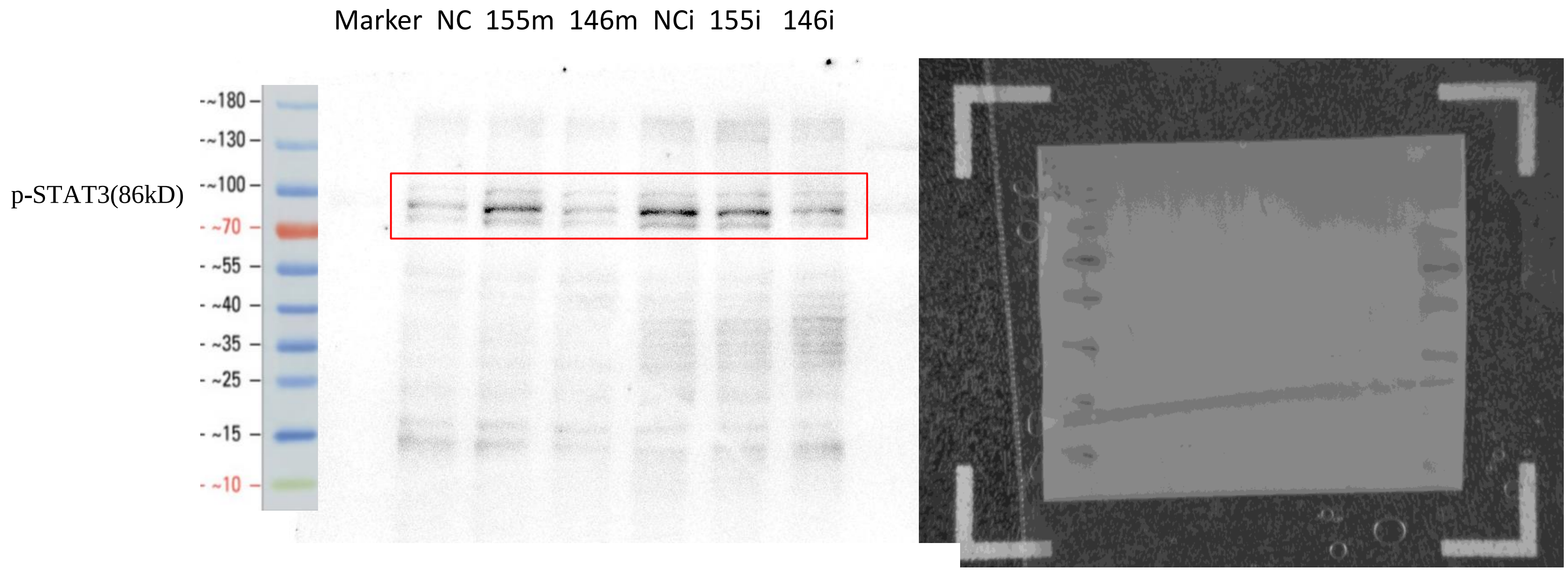
Marker NC 155m 146m NCi 155i 146i
p-STAT3(86kD)
~180
~130
~100
~70
~55
~40
~35
~25
~15
~10

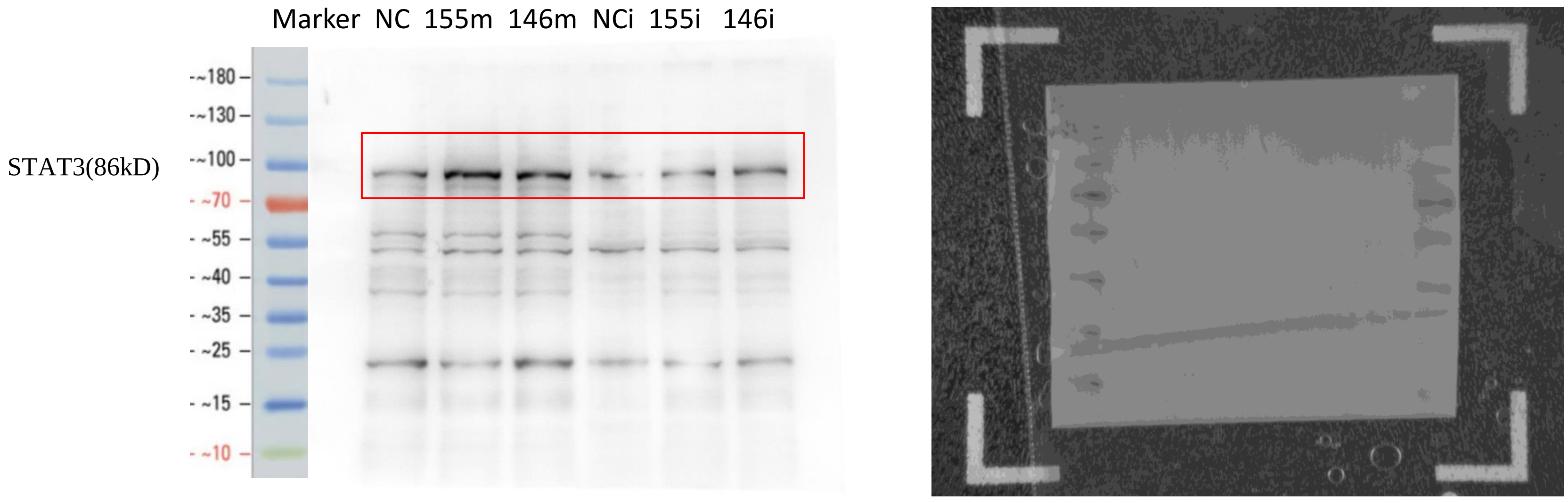
Marker NC 155m 146m NCi 155i 146i
STAT3(86kD)
~180
~130
~100
~70
~55
~40
~35
~25
~15
~10

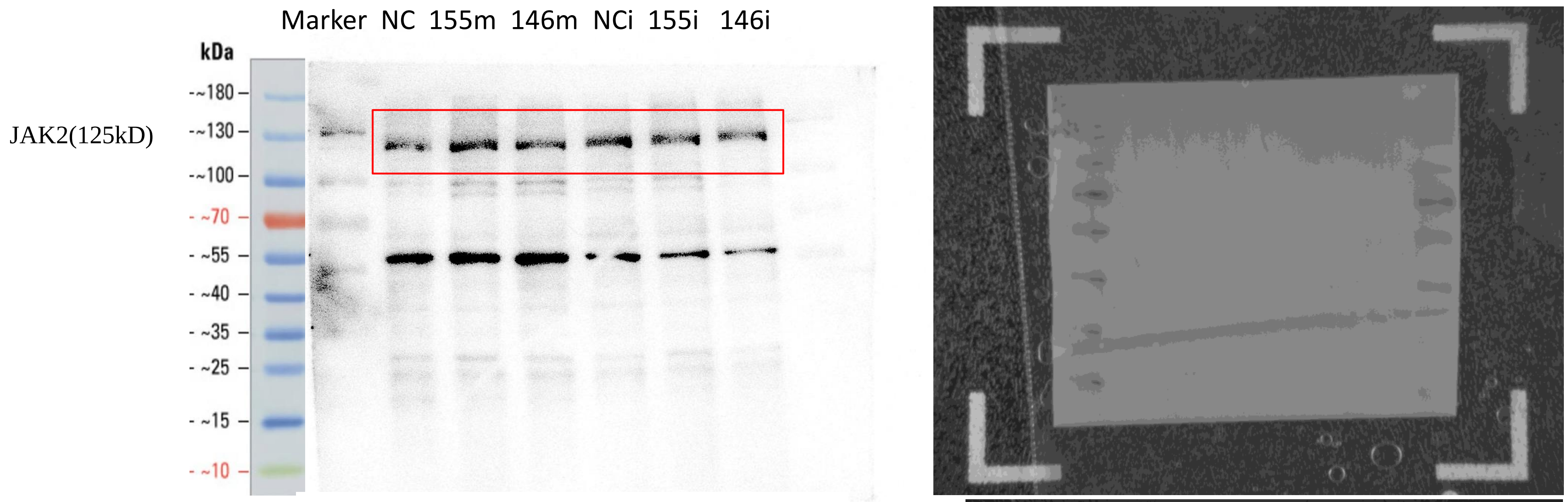
Marker NC 155m 146m NCi 155i 146i
kDa
JAK2(125kD)
~180
~130
~100
~70
~55
~40
~35
~25
~15
~10

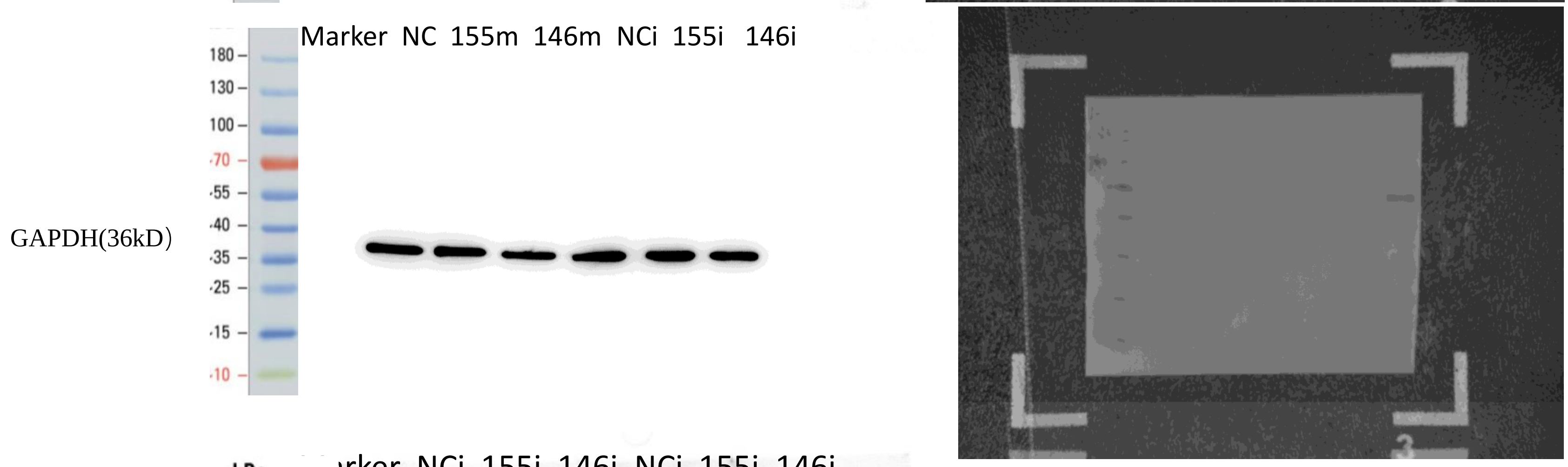
Marker NC 155m 146m NCi 155i 146i
GAPDH(36kD)
180
130
100
70
55
40
35
25
15
10

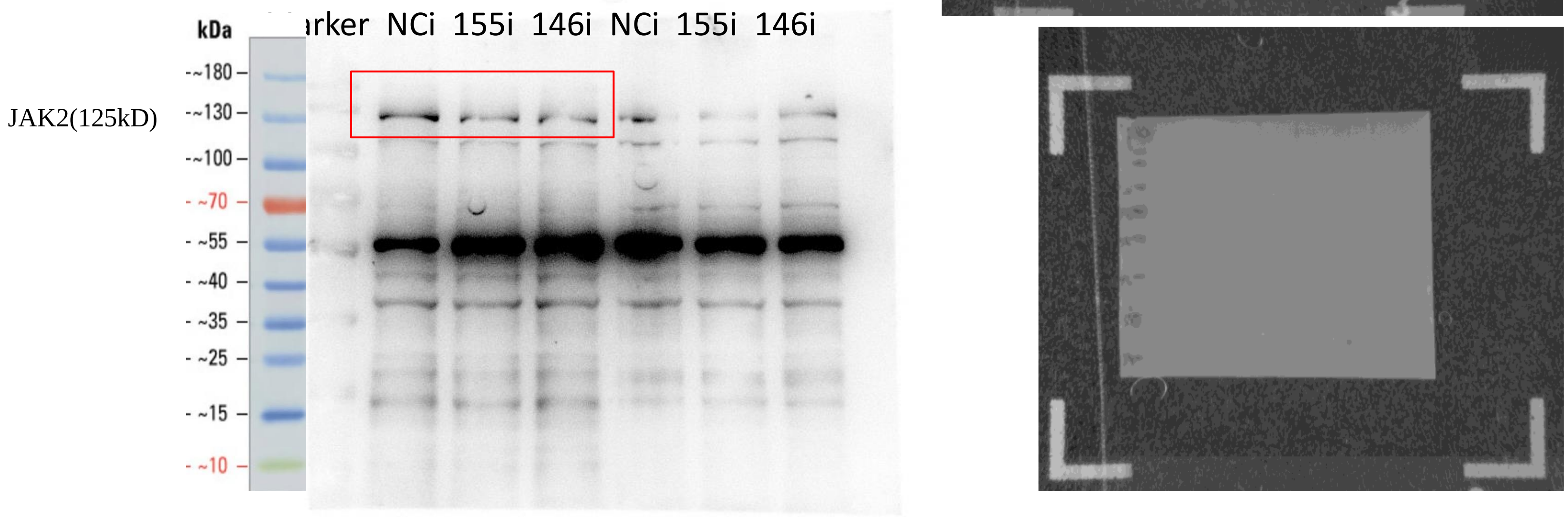
kDa
rker NCi 155i 146i NCi 155i 146i
JAK2(125kD)
~180
~130
~100
~70
~55
~40
~35
~25
~15
~10

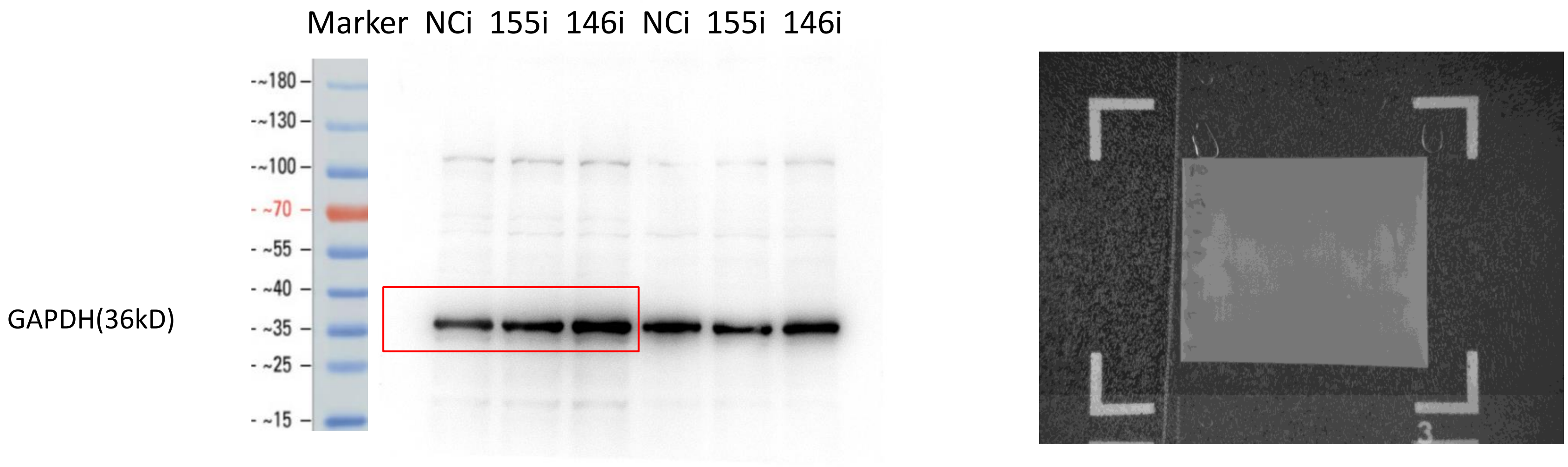
Marker NCi 155i 146i NCi 155i 146i
GAPDH(36kD)
~180
~130
~100
~70
~55
~40
~35
~25
~15

Figure 5E

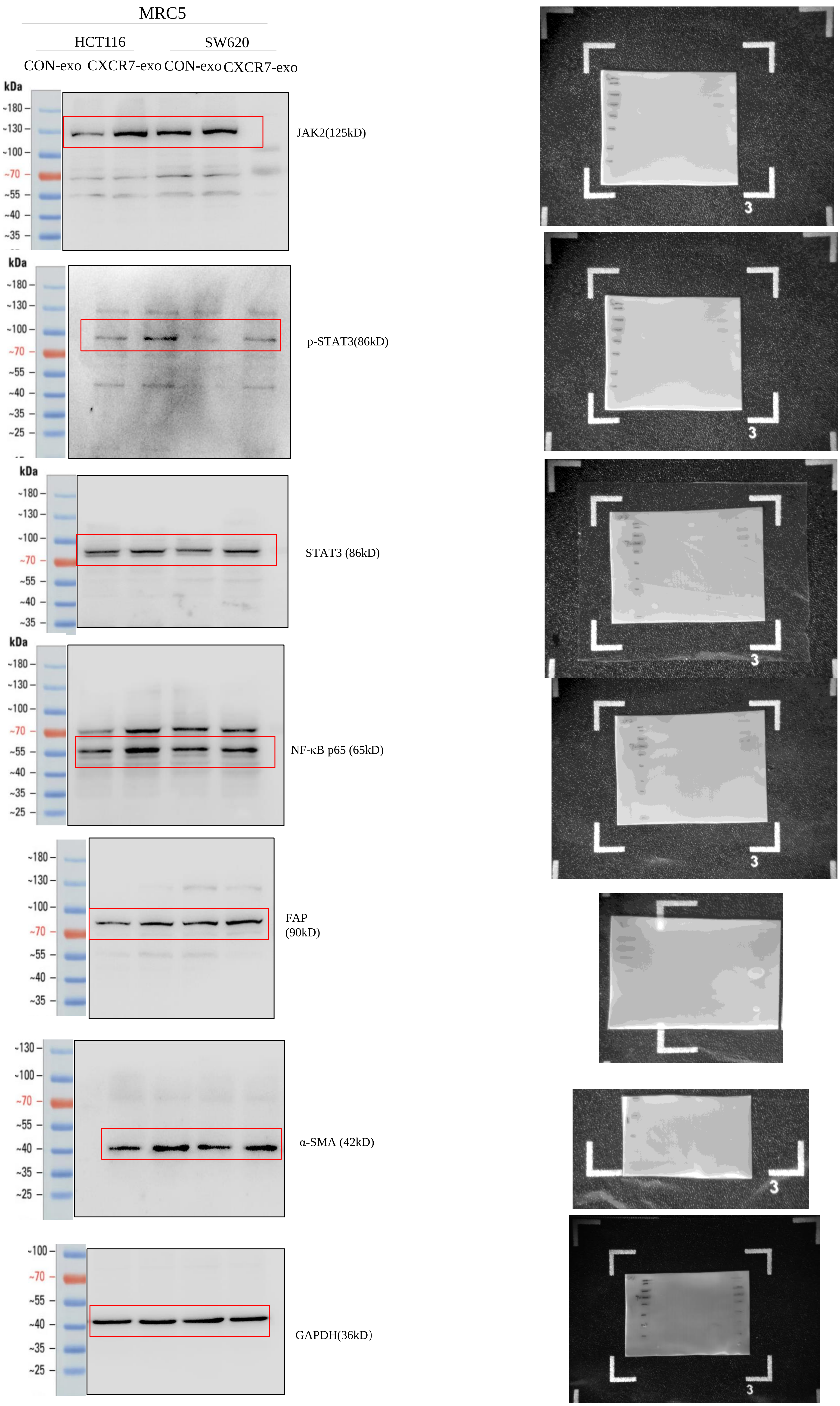
MRC5
HCT116
SW620
CON-exo
CXCR7-exo
CON-exo
CXCR7-exo
kDa
JAK2(125kD)
p-STAT3(86kD)
STAT3 (86kD)
NF-κB p65 (65kD)
FAP
(90kD)
α-SMA (42kD)
GAPDH(36kD)

Figure 5E

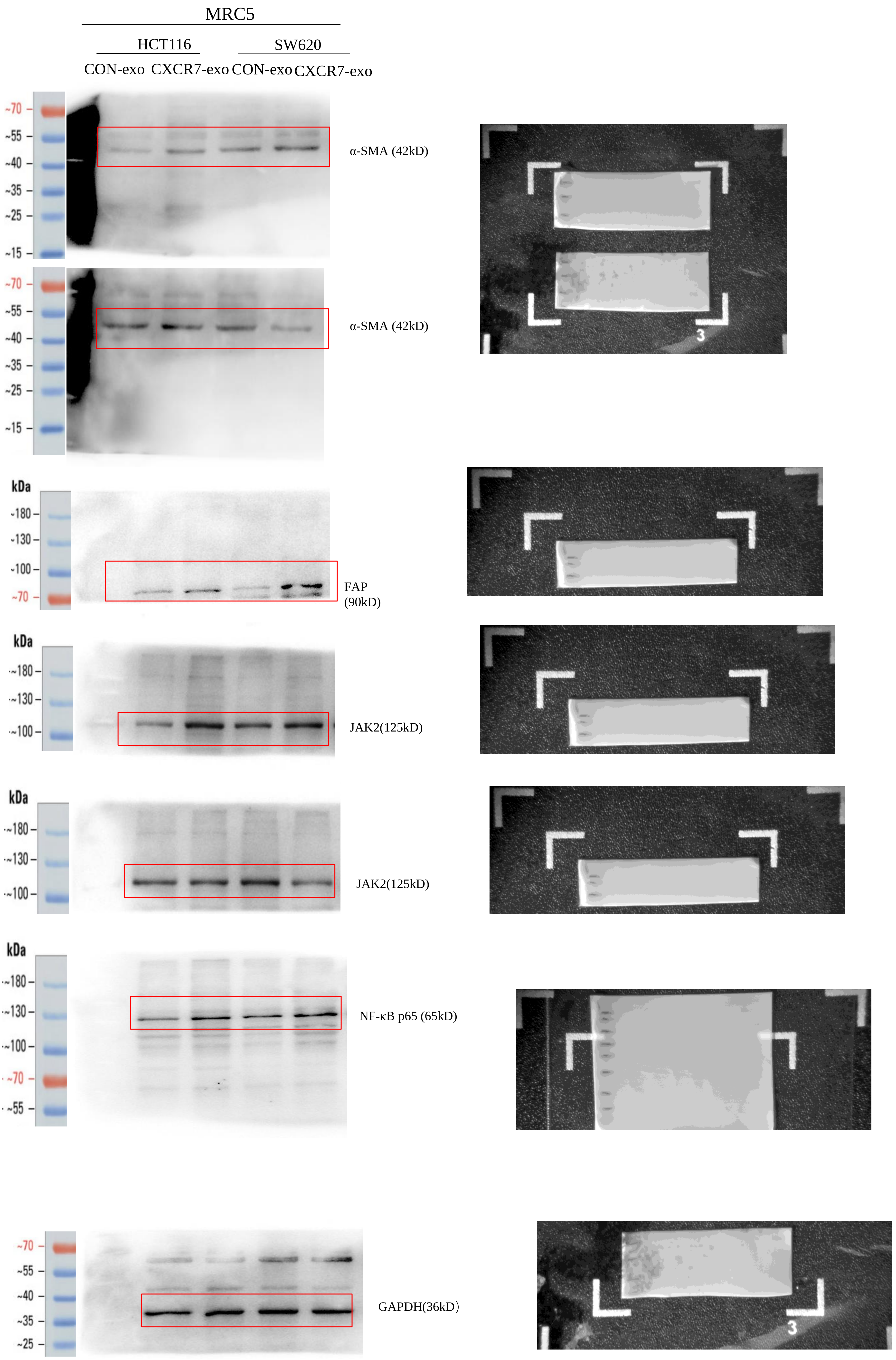
MRC5
HCT116
SW620
CON-exo
CXCR7-exo
CON-exo
CXCR7-exo
α-SMA (42kD)
α-SMA (42kD)
FAP (90kD)
JAK2(125kD)
JAK2(125kD)
NF-κB p65 (65kD)
GAPDH(36kD)
kDa
~180
~130
~100
~70
~55
~40
~35
~25
~15

Figure 5E

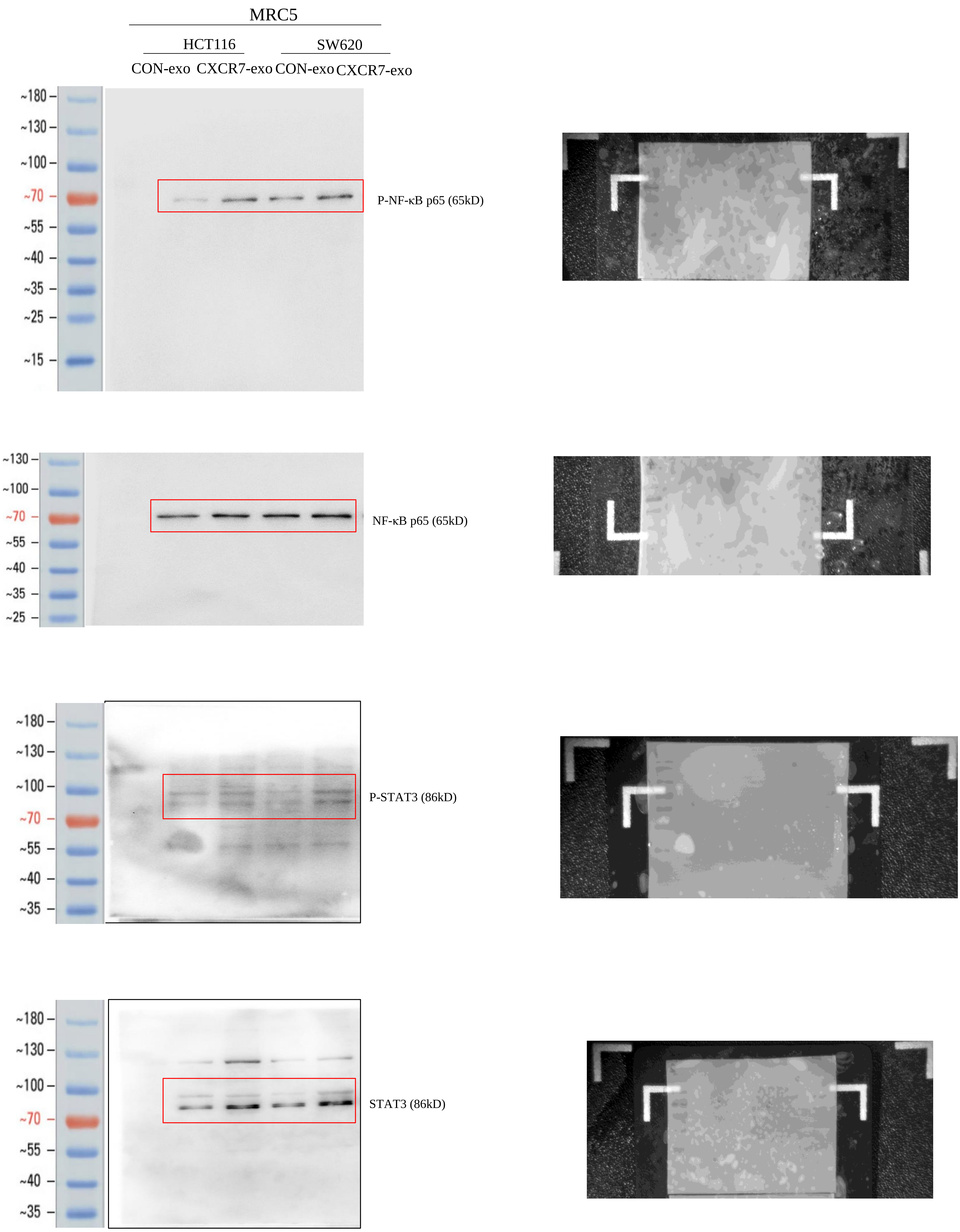
MRC5
HCT116
SW620
CON-exo
CXCR7-exo
CON-exo
CXCR7-exo
~180
~130
~100
~70
~55
~40
~35
~25
~15
P-NF-κB p65 (65kD)
NF-κB p65 (65kD)
P-STAT3 (86kD)
STAT3 (86kD)

Figure 5E

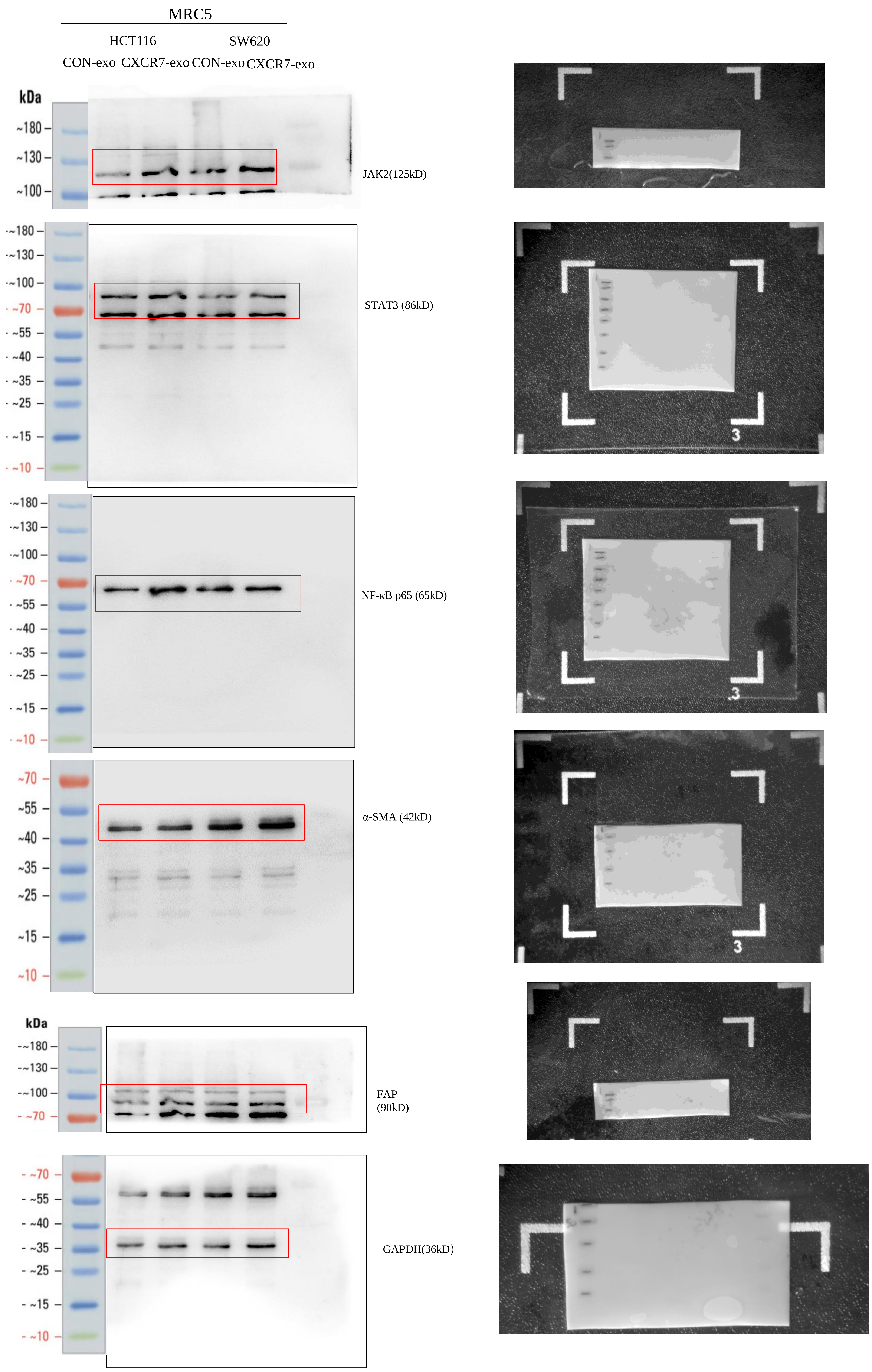
MRC5
HCT116
SW620
CON-exo
CXCR7-exo
CON-exo
CXCR7-exo
kDa
~180
~130
~100
~70
~55
~40
~35
~25
~15
~10
JAK2(125kD)
STAT3 (86kD)
NF-κB p65 (65kD)
α-SMA (42kD)
FAP
(90kD)
GAPDH(36kD)
3

Figure 5E

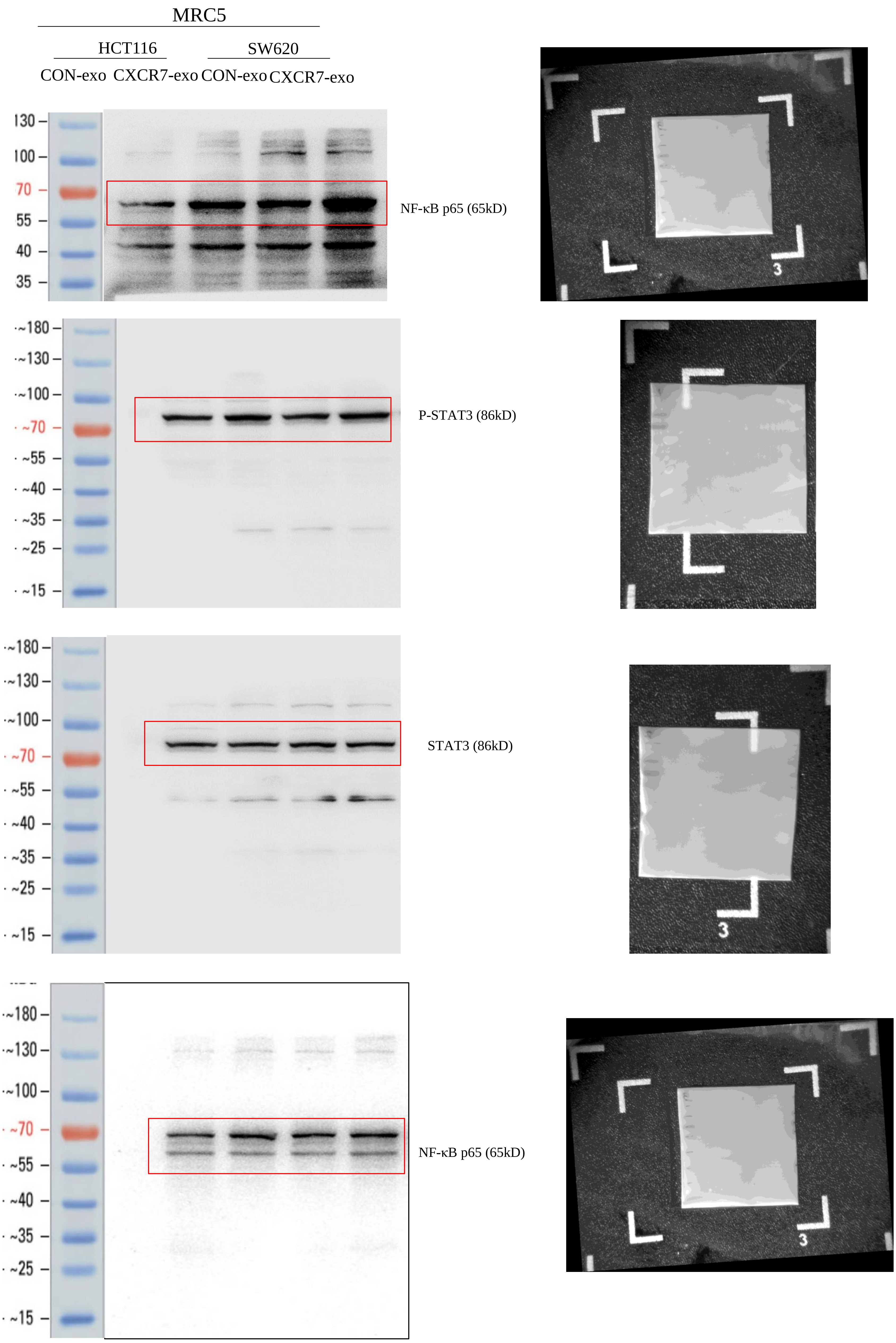

MRC5
HCT116
SW620
CON-exo
CXCR7-exo
CON-exo
CXCR7-exo
130
100
70
55
40
35
NF-κB p65 (65kD)
~180
~130
~100
~70
~55
~40
~35
~25
~15
P-STAT3 (86kD)
~180
~130
~100
~70
~55
~40
~35
~25
~15
STAT3 (86kD)
~180
~130
~100
~70
~55
~40
~35
~25
~15
NF-κB p65 (65kD)
3
3
3

Fig. 6C

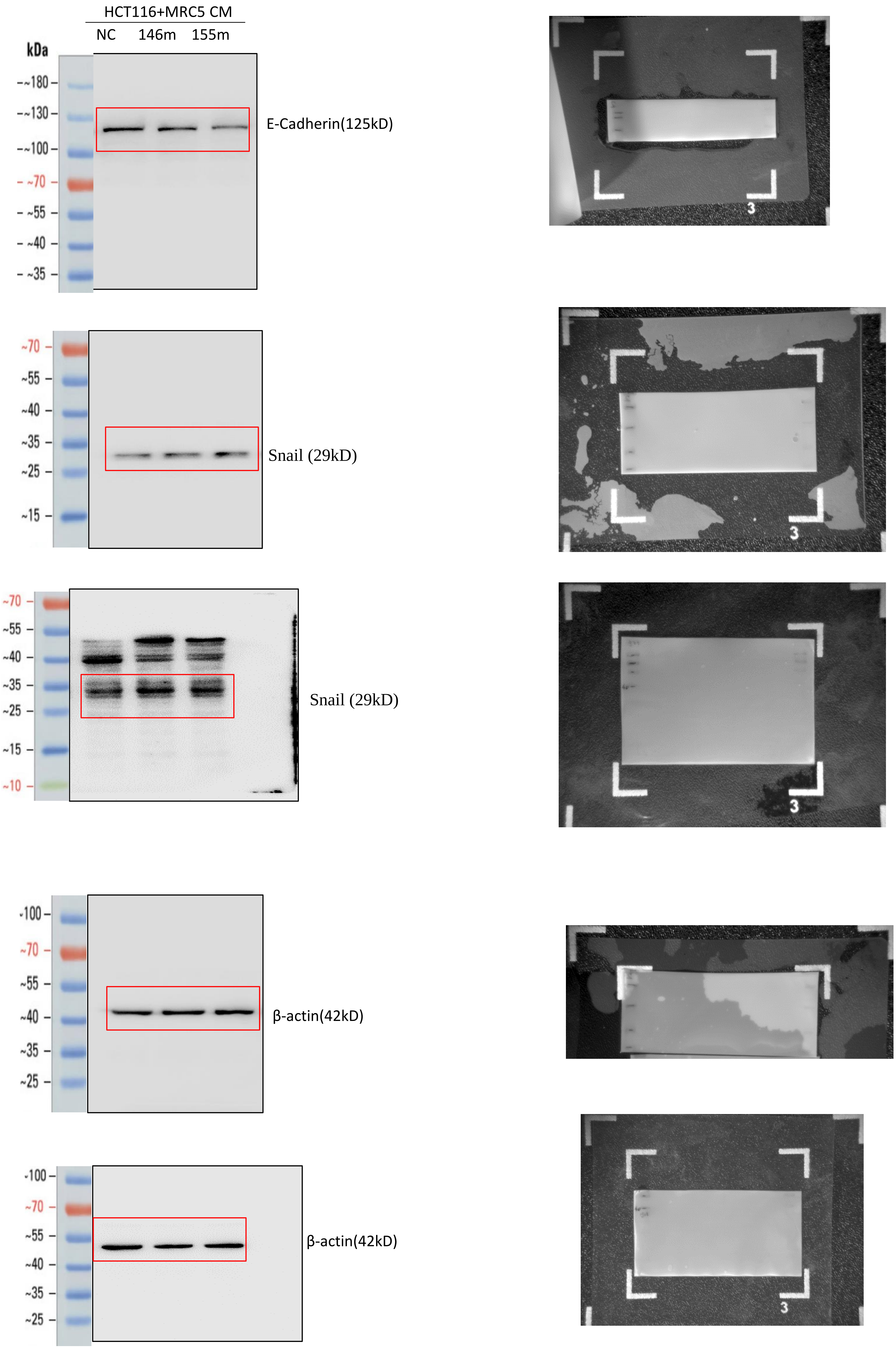

HCT116+MRC5 CM
NC
146m
155m
kDa
~180
~130
~100
~70
~55
~40
~35
~25
~15
~10
E-Cadherin(125kD)
Snail (29kD)
Snail (29kD)
β-actin(42kD)
β-actin(42kD)
3

Fig. 6C

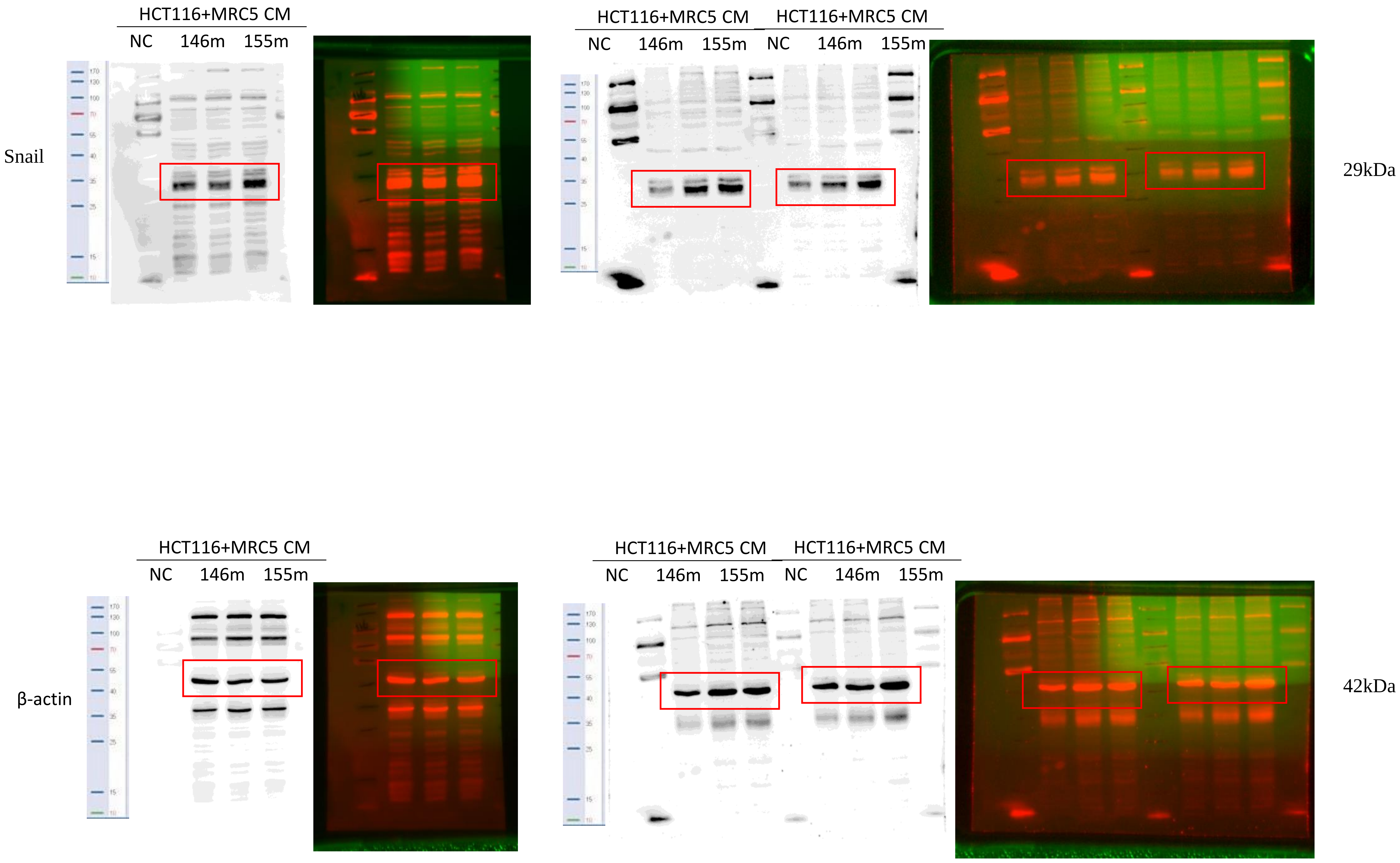
HCT116+MRC5 CM
NC 146m 155m
Snail
HCT116+MRC5 CM
NC 146m 155m
HCT116+MRC5 CM
NC 146m 155m
29kDa
HCT116+MRC5 CM
NC 146m 155m
β-actin
HCT116+MRC5 CM
NC 146m 155m
HCT116+MRC5 CM
NC 146m 155m
42kDa

Fig. 6C

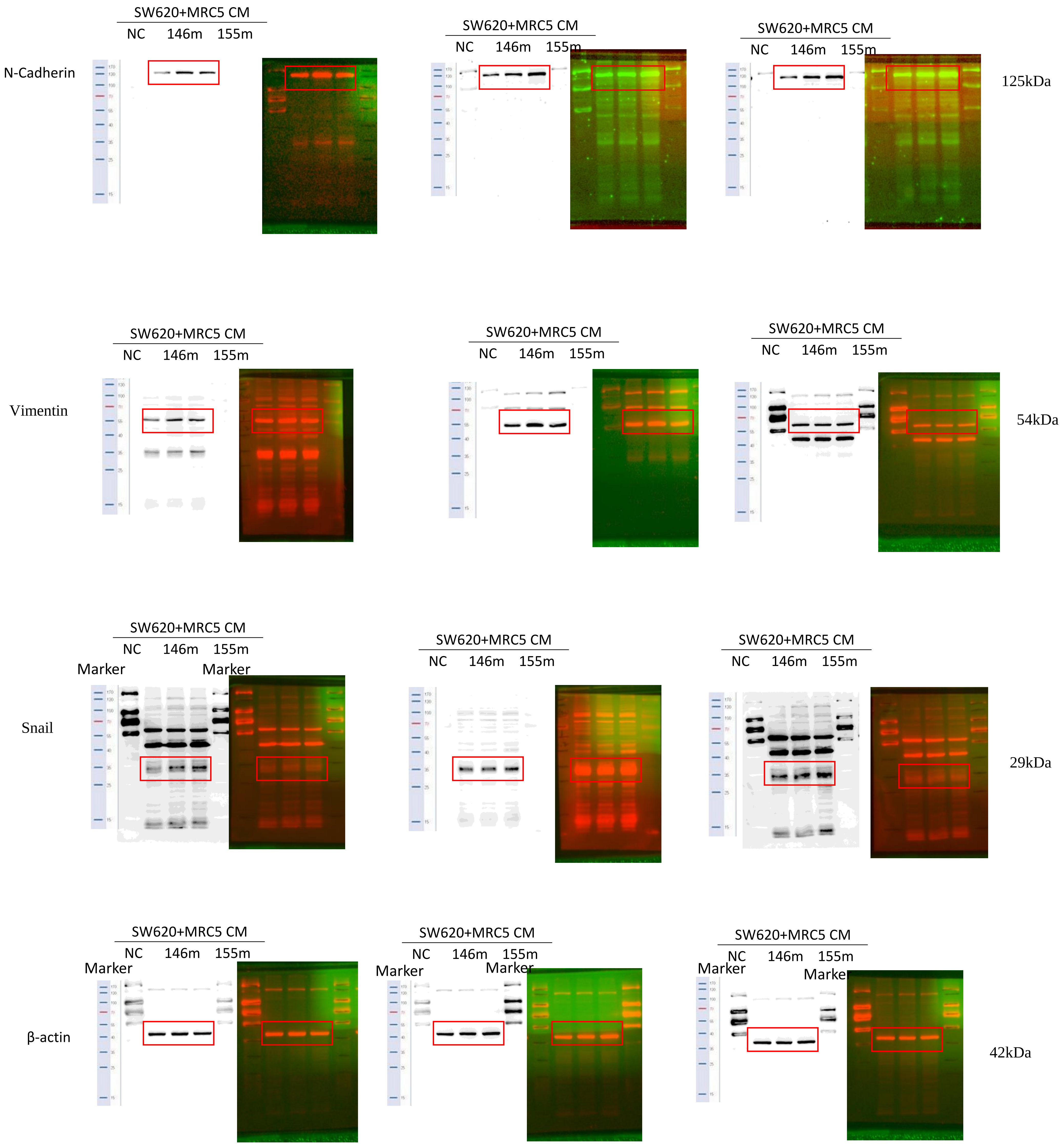
SW620+MRC5 CM
NC 146m 155m
N-Cadherin
125kDa
Vimentin
54kDa
Snail
Marker
29kDa
β-actin
42kDa

Figure 6D

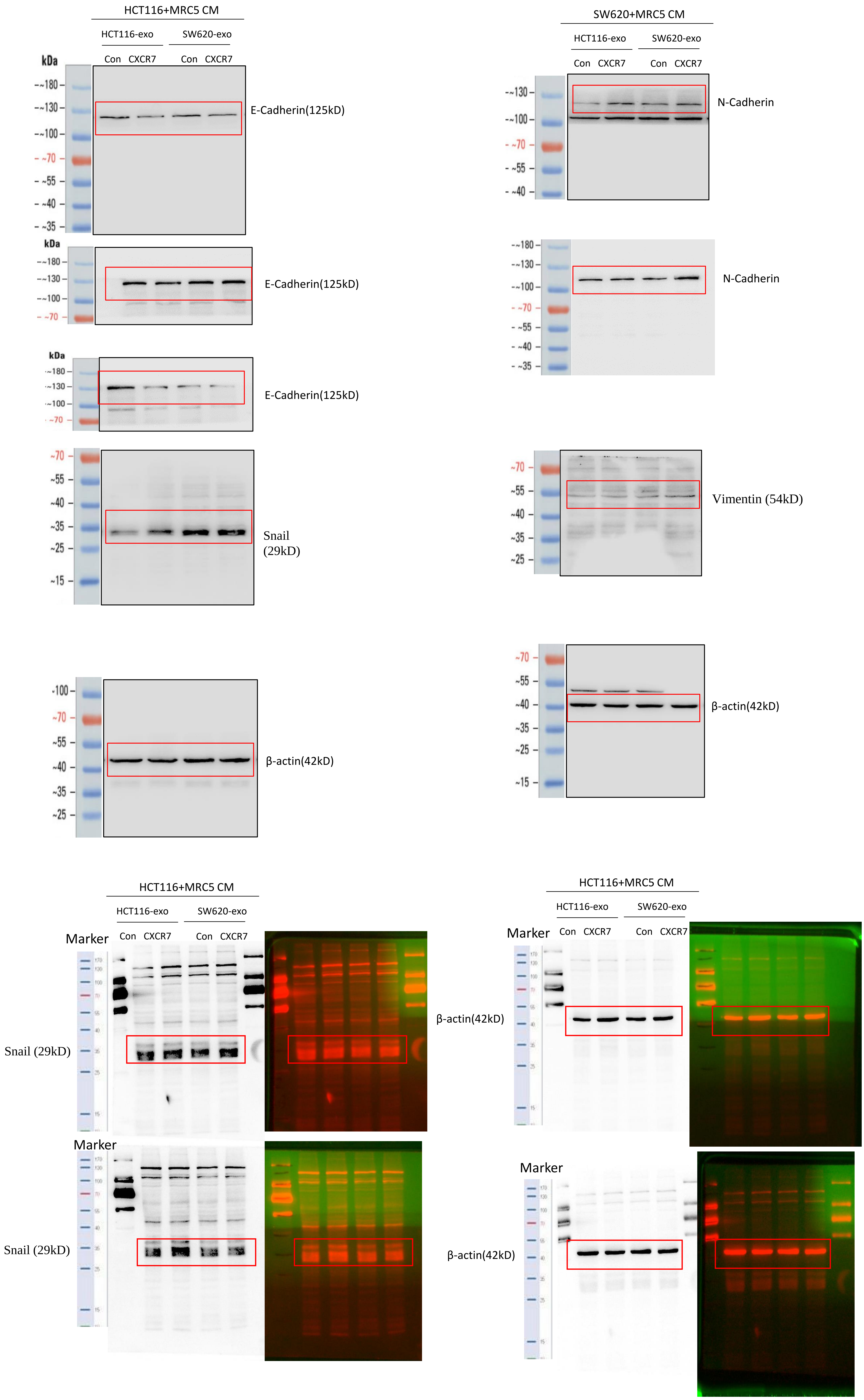
HCT116+MRC5 CM
HCT116-exo
SW620-exo
Con
CXCR7
Con
CXCR7
kDa
E-Cadherin(125kD)
E-Cadherin(125kD)
E-Cadherin(125kD)
Snail
(29kD)
β-actin(42kD)
SW620+MRC5 CM
HCT116-exo
SW620-exo
Con
CXCR7
Con
CXCR7
N-Cadherin
N-Cadherin
Vimentin (54kD)
β-actin(42kD)
HCT116+MRC5 CM
HCT116-exo
SW620-exo
Marker
Con
CXCR7
Con
CXCR7
Snail (29kD)
β-actin(42kD)
Marker
Snail (29kD)
HCT116+MRC5 CM
HCT116-exo
SW620-exo
Marker
Con
CXCR7
Con
CXCR7
Marker
β-actin(42kD)

Figure 6D

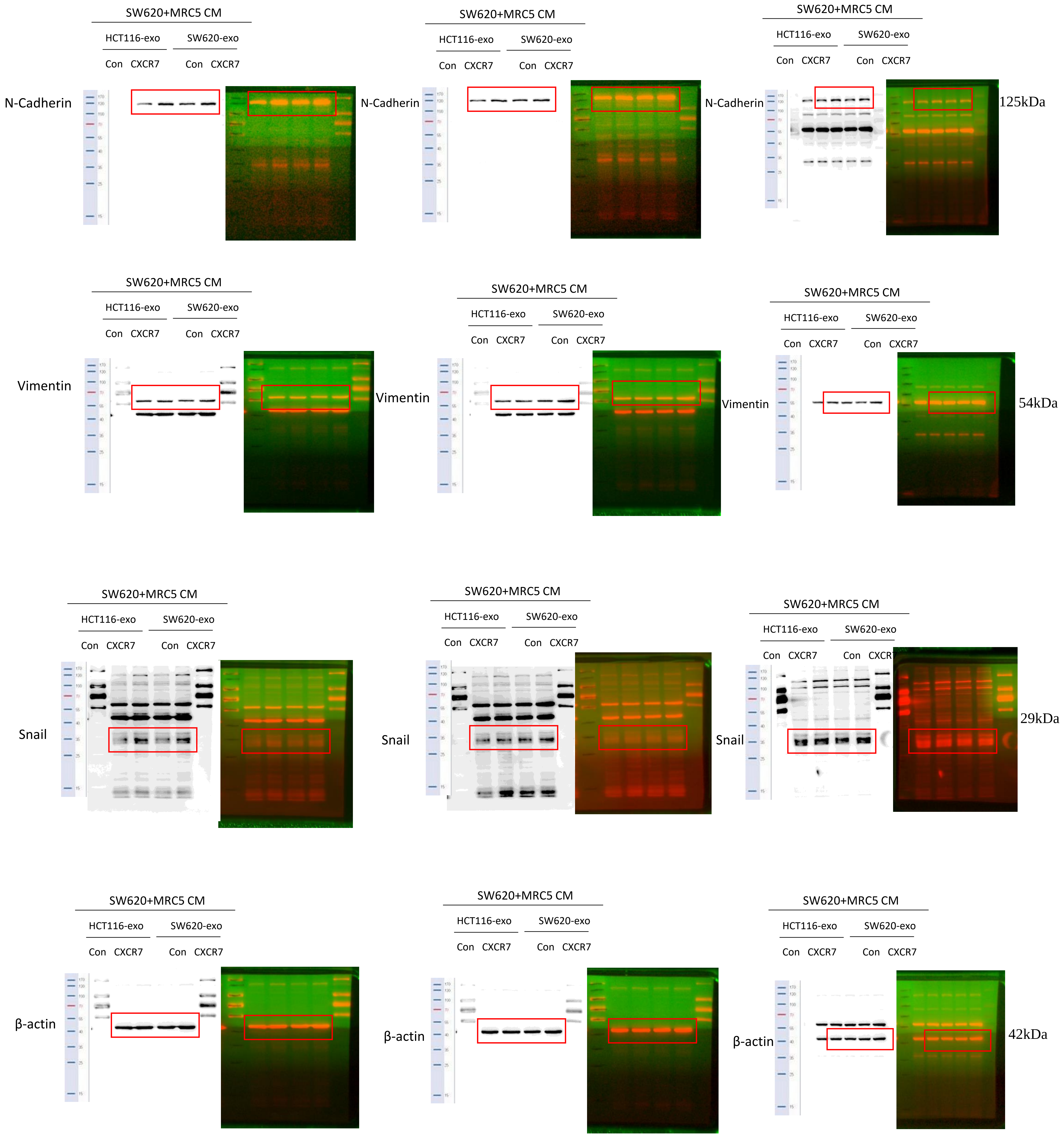

SW620+MRC5 CM
HCT116-exo
SW620-exo
Con CXCR7 Con CXCR7
N-Cadherin
125kDa
Vimentin
54kDa
Snail
29kDa
β-actin
42kDa

Figure 7C

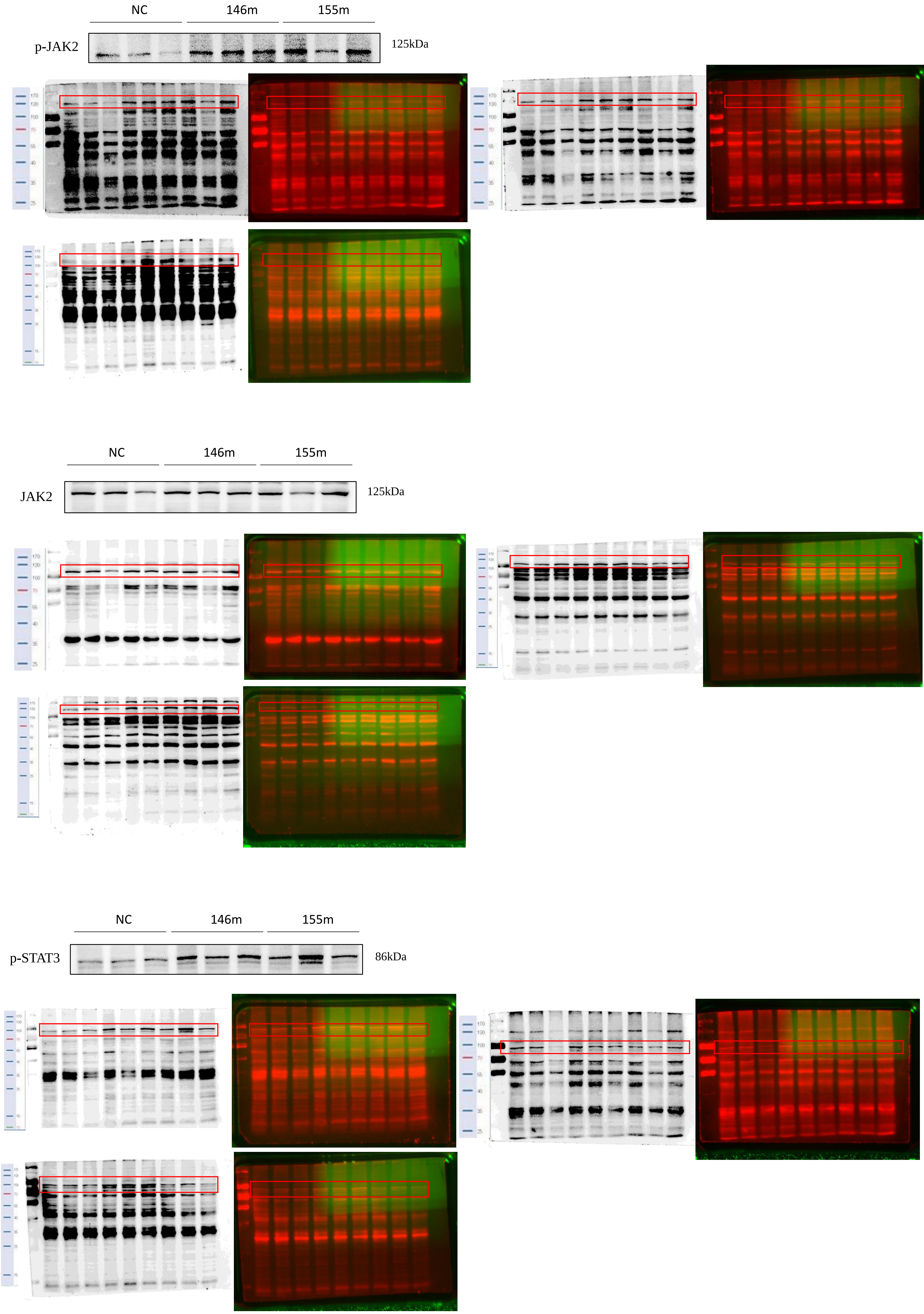
NC
146m
155m
p-JAK2
125kDa
NC
146m
155m
JAK2
125kDa
NC
146m
155m
p-STAT3
86kDa

Figure 7C

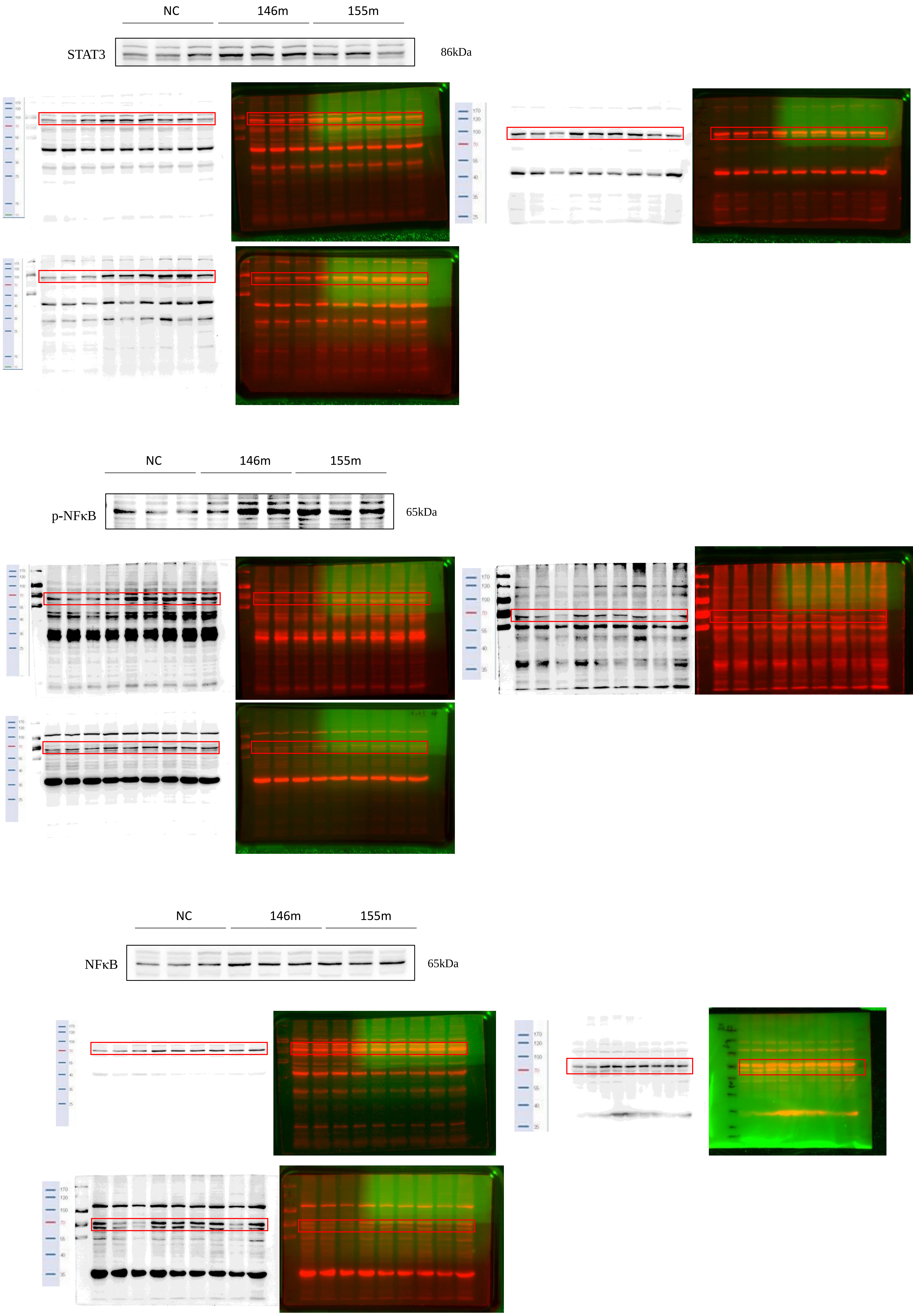
NC
146m
155m
STAT3
86kDa
NC
146m
155m
p-NFκB
65kDa
NC
146m
155m
NFκB
65kDa

Figure 7C

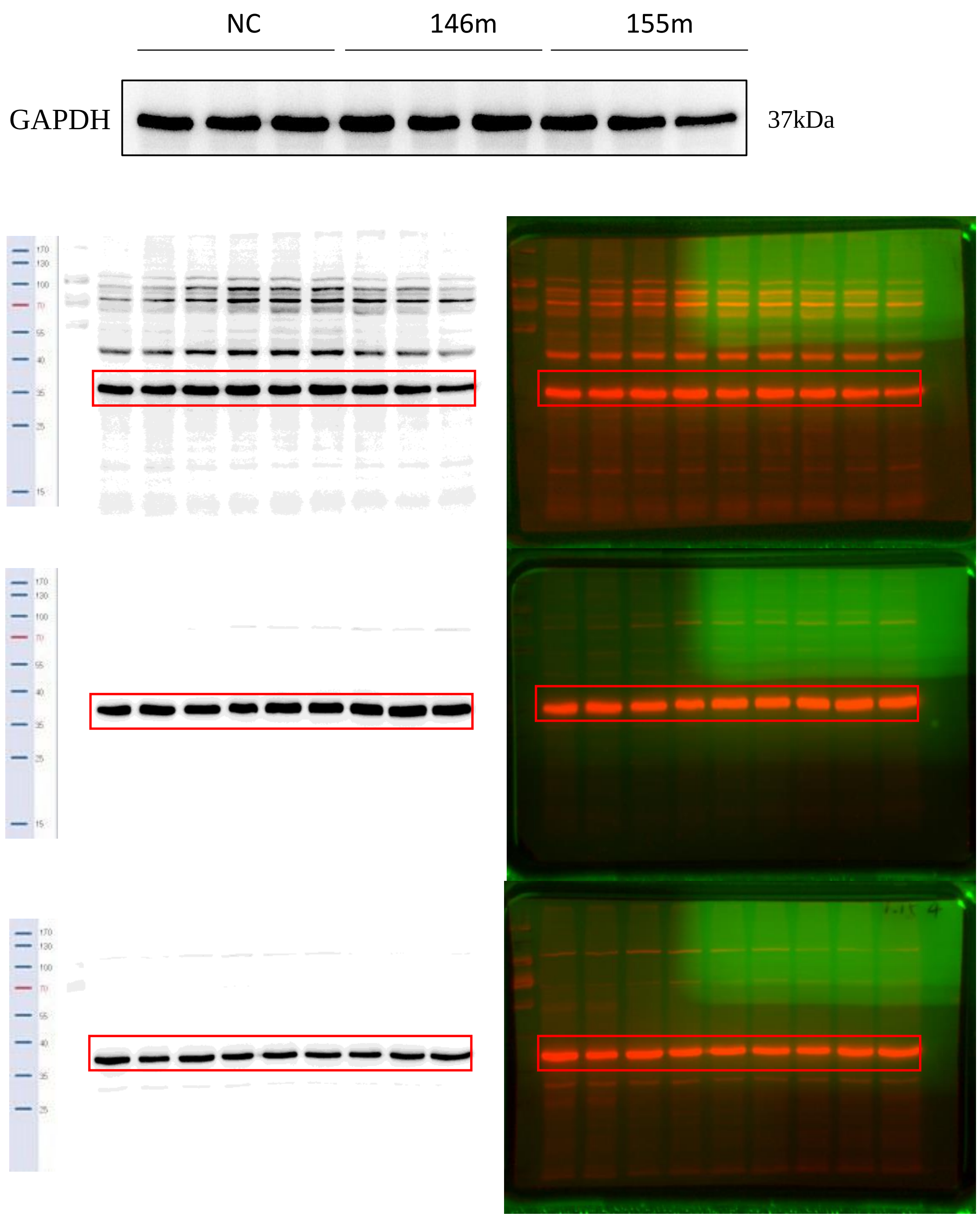

Figure 8C

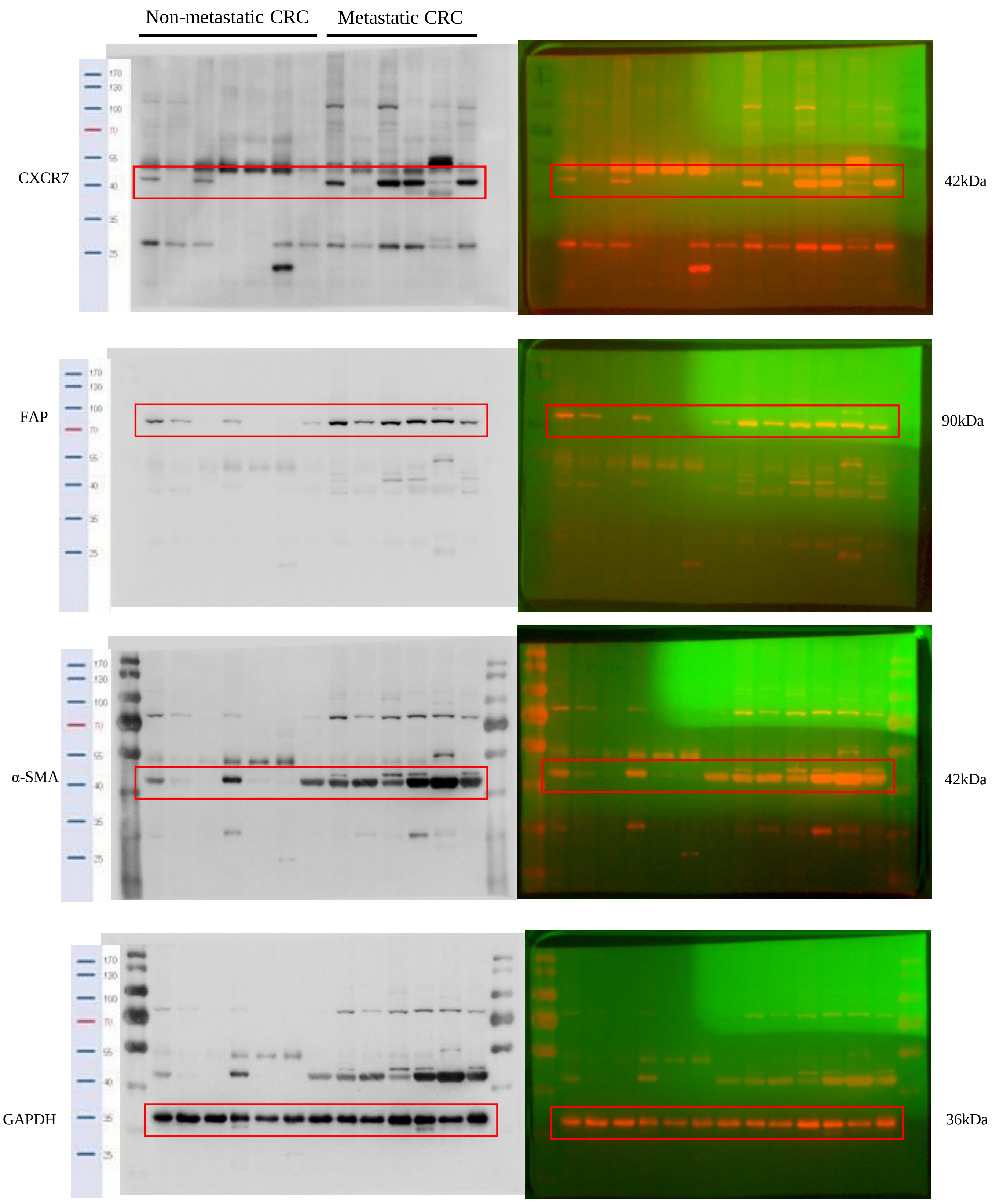
Non-metastatic CRC
Metastatic CRC
CXCR7
42kDa
FAP
90kDa
α-SMA
42kDa
GAPDH
36kDa

Fig. S5

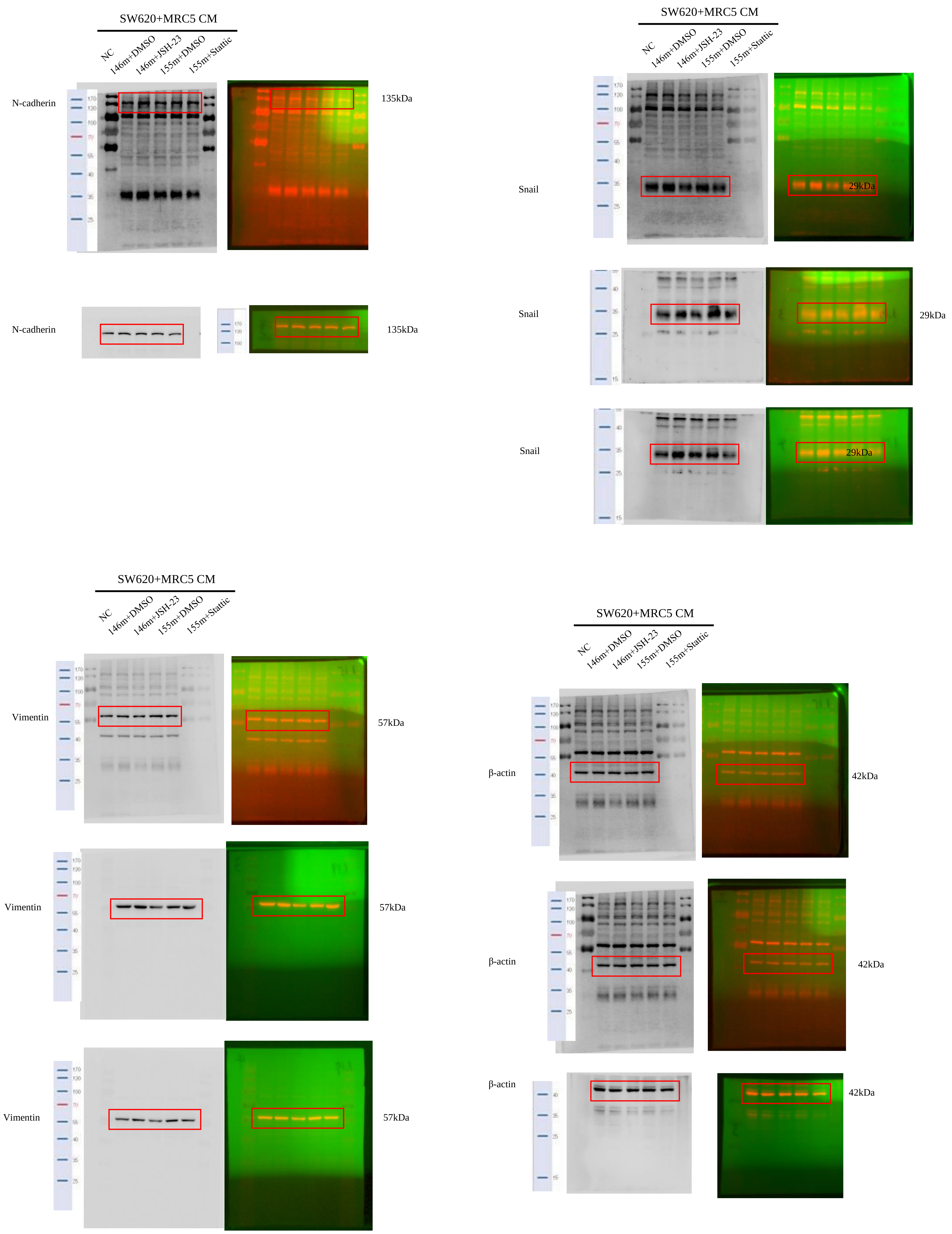

SW620+MRC5 CM
NC
146m+DMSO
146m+JSH-23
155m+DMSO
155m+Stattic
N-cadherin
135kDa
N-cadherin
135kDa
Snail
29kDa
Snail
29kDa
Snail
29kDa
Vimentin
57kDa
Vimentin
57kDa
Vimentin
57kDa
β-actin
42kDa
β-actin
42kDa
β-actin
42kDa
